# Machine learning for risk stratification of hypertensive disorders of pregnancy: Enhancing clinical efficiency in low-resource antenatal care in Tanzania

**DOI:** 10.1371/journal.pdig.0001468

**Published:** 2026-07-02

**Authors:** Isaac Lyatuu, Emmanuel P. Mwanga, Yusuph Kulindwa, Raymond Bandio, Alen Kinyina, Ntuli Kapologwe, Phineas Sospeter, Ahmad Makuwani, James Tumaini, Omari Sukari, Augustino Hellar

**Affiliations:** 1 Prime Health Initiative Tanzania (PHIT), Dar-es-Salaam, Tanzania; 2 Ifakara Health Institute (IHI), Dar-es-Salaam, Tanzania; 3 Ministry of Health (MoH), Dodoma, Tanzania; 4 President’s Office Regional Authority and Local Government (PORALG), Dodoma, Tanzania; 5 Regional Health Secretariat, Geita Region, Tanzania; Hadassah Academic College, ISRAEL

## Abstract

Maternal mortality in Tanzania remains a public health crisis, with Hypertensive Disorders of Pregnancy (HDP) causing 34% of direct obstetric deaths. In overburdened government clinics, high patient volumes and limited resources often restrict assessments to single-point blood pressure checks, leading to missed diagnoses. This study investigates the potential of machine learning (ML) to move beyond simple threshold detection toward automated risk stratification, aiming to optimize patient flow and prioritize clinical resources for high-risk individuals. We analyzed 337,027 routine records (2023–2024) from Tanzania’s Unified Community System (UCS). Data from multiple visits were aggregated into 187,438 unique client records. HDP was defined by standard clinical thresholds (BP ≥ 140/90 mmHg). We trained five ML models on a balanced subset and validated the top performer on an independent dataset of over 120,000 records to evaluate its utility as a triage tool. XGBoost was the best performing model, achieving 90.1% accuracy and an AUC of 0.95. The model maintained 100% sensitivity, successfully stratifying 12,603 clients into the high-risk category, including those potentially overlooked by traditional checks. While precision was 14% (representing 6.3 false positives per true case), this high-sensitivity screening approach ensures no at-risk client is missed, allowing providers to focus intensive assessment time where it is most needed. ML-driven risk stratification can transform congested ANC workflows by identifying high-risk clients before they escalate to critical states. By automating the initial triage, health facilities can improve operational efficiency and ensure limited specialist time is dedicated to the most vulnerable patients. We recommend that the Ministry of Health strengthens digital data integration to support the deployment of these stratification tools within routine primary care.

## Author summary

Maternal mortality remains a critical health crisis in Tanzania, where high blood pressure disorders during pregnancy account for over one-third of direct maternal deaths. In busy government clinics, healthcare workers face overwhelming patient volumes and shortages of resources, meaning prenatal checks are often reduced to quick blood pressure readings that can easily miss evolving complications. To address this challenge, our study explored how machine learning can automatically identify and sort high-risk patients, helping clinics better manage patient flow and prioritize limited resources. We analyzed over 337,000 routine health records from Tanzania’s unified health database to train and test several computer models. Our top-performing model achieved 90.1% accuracy and successfully identified every single high-risk patient, ensuring no vulnerable individual is missed. While this high-sensitivity approach flags some patients who ultimately do not develop complications, it acts as a highly effective safety net. By automating early risk detection, health facilities can dramatically improve operational efficiency and ensure that busy specialists spend their limited time on the patients who need them most.

## Introduction

Hypertensive Disorders of Pregnancy (HDP) affect an estimated 5–10% of pregnancies in Tanzania [1], contributing to approximately 34% of direct maternal deaths. To address this burden, there is an urgent need for tools that can move beyond passive monitoring toward active risk stratification.

Machine Learning (ML) techniques have recently gained significant attention to predicting maternal health outcomes. Established clinical risk prediction tools such as the fullPIERS and PREP-S models have demonstrated good predictive performance for adverse maternal outcomes in pre-eclampsia and are recommended as adjuncts to clinical assessment in several guidelines [2–7]. However, most existing studies rely on curated research datasets [6,8], that fail to capture data quality challenges inherent in routine Health Management Information Systems (HMIS), including missing values, measurement inconsistencies, and documentation gaps [9]. Recent systematic reviews highlight this parallel rise in ML applications [6,10–12], yet few models have been developed or validated using routine government data from low-resource settings.

Existing HDP models report strong performance with AUC ranging from 0.81 to 0.91 [2,3] and accuracy up to 84% [4]. However, these results typically stem from standardized environments with complete data. Furthermore, most published studies report high precision (often >70%) [4,5], which may not reflect the realities of imbalanced, real-world datasets.

In contrast, this study utilizes routine government Antenatal Care (ANC) data from over 120,000 clients to achieve comparable sensitivity and AUC. Our model results in a lower precision (14%), which is a direct consequence of using “noisy,” passively collected health system data.

In alignment with WHO recommendations advocating for early detection [13], ML-driven stratification offers significant promise for prioritizing care in overburdened systems. The Government of Tanzania (GoT) has already laid the groundwork through the Unified Community System (UCS) [14,15], a digital platform for remote data collection in low-resource settings. However, the potential of this data remains underutilized; many expectant mothers attend fewer than the recommended visits [16–18], and reliance on paper-based backups [18], often prevents the triangulation of data for population-level insights. At the same time, the deployment of ML-based decision-support tools in such contexts requires careful attention to ethical and implementation considerations, including data quality, representativeness, algorithmic bias, transparency of model predictions, and the need to ensure that automated systems augment rather than replace clinical judgment [19–21]. Addressing these challenges is particularly important in low- and middle-income countries where health information systems are evolving, and data completeness can vary substantially across facilities. This study aims to bridge this gap by demonstrating how routine UCS data can be leveraged to stratify maternal risk and improve clinical efficiency within real-world health system constraints.

## Study objectives

This study investigates the application of ML to enhance risk stratification for HDP in low-resource settings using routine ANC data. The specific objectives were:

To assess the quality and usability of routine ANC data from Tanzania’s UCS, specifically addressing the challenges of incomplete and “noisy” health records for machine learning applications.
To develop and train a machine learning model that integrates multiple maternal health indicators to provide automated risk stratification, moving beyond single-point blood pressure threshold detection.
To optimize and validate the model’s performance on an independent dataset, select a clinical decision threshold that prioritizes high sensitivity to ensure the tool functions effectively as a reliable early-warning screen for high-risk clients.
To evaluate the potential utility of this stratified approach in improving clinical efficiency and resource prioritization within congested, low-resource health facilities

To address these objectives, we conducted a retrospective cohort study using routine ANC data from Tanzania’s UCS, as described below.

## Methods

### Study design and participants

This retrospective cohort study utilized routine ANC data from Tanzania’s UCS to develop and validate a machine learning model for HDP risk stratification. The study population comprised all expectant mothers recorded in the UCS between 2020 and 2024 across 23 regions of Tanzania. These regions represent the current coverage of the UCS application, which is being phased into the remaining eight regions of the country.

### Data processing and feature engineering

Data were extracted from the UCS online portal and processed through a structured transformation pipeline. Initial cleaning involves converting data types, removing duplicate entries based on unique event identifiers, and correcting implausible physiological values. Using domain expertise, we addressed common digital entry errors (e.g., correcting “14.02” or “1402” to “140.2” mmHg) and removed extreme outliers exceeding three standard deviations from the mean.

### Data flattening strategy

We collapsed multiple visits into a single patient record due to extreme temporal sparsity (a median of one visit per client and only 43% of clients having two or more recorded visits). In the data collapse pipeline, we retained the last recorded value for categorical variables and the mean value for numerical variables. This “flattening” approach was critical for two reasons:

Generalizability: It prevented the exclusion of over 50% of the population, thereby reducing selection bias.
Clinical Realism: It mirrors the operational reality in Tanzania, where clinicians must often perform risk assessments based on a single point of contact.

#### Feature selection.

We employed a multi-stage hybrid approach to identify predictors. An initial candidate set was established by merging domain expertise with data availability constraints. This set was then statistically screened using Pearson correlation, Chi-square tests, and Mutual Information scores to capture both linear and non-linear associations. While Recursive Feature Elimination (RFE) identified a parsimonious subset of three features (systolic BP, diastolic BP, and syphilis), we utilized SHAP (Shapley Additive exPlanations) and clinical consultation to retain a more comprehensive profile. The final model includes proteinuria, BMI, blood glucose, and temperature, ensuring the tool captures a broader biological context relevant to HDP risk.

### Missing data handling

We employed listwise deletion (complete case analysis) for the final analytic dataset. We deliberately avoided imputation for two primary reasons:

**Non-Random Missingness:** In routine health systems, data are often “Missing Not At Random” (MNAR); for instance, diagnostic tests are more likely to be missing in resource-constrained facilities or among patients perceived as low-risk, which would introduce bias if imputed.
**Real-World Fidelity:** Imputation can create an “artificial precision” that masks the inherent uncertainty and data gaps typical of routine HMIS data.

After flattening, any client record missing any of the seven core features (systolic/diastolic BP, BMI, blood glucose, proteinuria, temperature, and syphilis) was excluded. Table 2 in the results section details the missingness rates of selected features prior to exclusion to ensure transparency regarding the original data quality.

**Table 2:** Missingness rates for key features in the original dataset before collapse (N = 337,027).

Feature	Missing Count	Missing Rate
blood_for_glucose	256,342	76%
rh_factor	243,315	72%
fetal_heart_rate	211,912	63%
fundal_height	211,906	63%
bmi	167,104	50%
temperature	167,101	50%
systolic	167,101	50%
diastolic	167,100	50%
weight	167,100	50%
syphilis	149,979	45%
blood_group	142,987	42%
protein_in_urine	100,438	30%
glucose_in_urine	100,436	30%
height	257	~ 0.0%
gest_age	47	~ 0.0%
client_id	–	0.0%
visit_number	–	0.0%

### Outcome definition

The primary outcome HDP was defined according to the Tanzania’s ANC national guideline as systolic blood pressure ≥140 mmHg and/or diastolic blood pressure ≥90 mmHg [22].

### Rationale for algorithmic labeling

In this study, HDP status was derived algorithmically from routine BP measurements rather than relying on documented clinical diagnoses. This decision was necessitated by severe underreporting and data quality concerns in the UCS dataset. Epidemiological estimates indicate that hypertensive disorders of pregnancy affect approximately 5–10% of pregnancies in Tanzania; however, fewer than 0.5% of health records contain a completed diagnosis field due to due to persistent documentation challenges [23]. Consultations with implementation partners identified three primary drivers for this gap:

**Data Entry Lag:** Clinical events are often not recorded in real-time, leading to omitted diagnoses during retrospective entry.
**System Fragmentation:** HDP cases are frequently managed in labor wards, but the lack of a floating unique patient identifier and existence of paper registers prevents linking delivery outcomes back to ANC records.
**Performance Bias:** Reluctance to document adverse outcomes due to facility-level performance metrics.

To address these limitations, we employed a rule-based outcome estimation informed by subject-matter experts. This approach classified cases based on clinical signs and onset indicators typical of HDP, providing a “silver standard” ground truth where a gold standard (confirmed clinical diagnosis) was unavailable.

### Addressing data leakage and model interpretation

We acknowledge that using BP measurements as both predictors and components of the algorithmic outcome introduces data leakage, which fundamentally shapes the interpretation of our results. Because the outcome is defined by the same BP thresholds that serve as primary predictors, the high AUC and sensitivity achieved do not represent the prediction of future disease. Instead, they demonstrate the model’s capacity for enhanced risk stratification: its ability to identify consistent patterns of BP elevation more reliably than a human clinician performing a single-visit threshold check in a “noisy” environment. This distinction is critical for clinical applications. The model functions as an advanced screening tool for BP-defined risk under real-world conditions of infrequent visits and measurement error. To mitigate the impact of leakage, we:

Incorporated a diverse set of predictive features beyond blood pressure (e.g., BMI, syphilis, proteinuria).
Validated the model on a fully independent dataset.
Conducted a discordant case analysis to demonstrate the model’s ability to “screen in” at-risk cases that might be missed by conventional, manual BP thresholding alone.

While these steps strengthen the model’s utility, the performance metrics must be interpreted strictly within the context of a BP-defined proximal outcome.

### ML classification

Several ML classification methods were evaluated to determine their suitability for risk stratification. We compared five widely used algorithms: K-Nearest Neighbor (KNN), Logistic Regression (LR), Support Vector Machine (SVM), Random Forest (RF), and Extreme Gradient Boosting (XGBoost). Rather than selecting a model based on raw accuracy alone (which can be misleading in imbalanced datasets), the optimal model was selected based on its ability to maintain high sensitivity (recall) while processing routine, noisy HMIS data.

### Clinical threshold selection for risk stratification

To ensure the model functions as a reliable safety screen, we moved away from default probability thresholds (0.5) or metrics like Youden’s Index that balance sensitivity and specificity equally. Instead, we intentionally calibrated the decision threshold to prioritize 100% sensitivity. While standard optimization (such as F1-score maximization) yielded a threshold of 0.995 with high precision but unacceptably low recall (2%), our approach prioritizes “screening in” all potential HDP cases. This ensures that in a congested facility, no at-risk client is missed, even at the cost of a higher false-positive rate (lower precision), which is clinically managed through standard follow-up BP confirmation.

#### Model calibration and Precision-Recall analysis.

The agreement between predicted probabilities and observed outcomes was assessed using Platt scaling to obtain calibrated probabilities. We evaluated calibration using: (1) calibration plots comparing predicted vs. observed risk across deciles; (2) the Brier score; and (3) calibration slope and intercept. Given the class imbalance (3.7% HDP prevalence), we prioritized Precision-Recall (PR) curves and Average Precision (AP) over ROC-AUC, as AP provides a more rigorous assessment of the model’s performance on the rare positive class [24].

#### Sampling strategy and class balancing.

The analytic dataset exhibited severe class imbalance (1,894 HDP cases vs. 48,680 controls). To address this, we employed random under-sampling of the majority class [25] to create a balanced training set (50/50 distribution). This approach was chosen over synthetic methods like SMOTE to ensure the model learned from authentic clinical patterns rather than generated data points, which can exacerbate noise in routine health records. Under-sampling was applied strictly to the training set; the validation set retained the natural 3.7% prevalence to ensure the performance metrics reflect real-world clinical utility.

#### Model training, testing, and validation.

The dataset was split into 90% for training/testing and 10% for internal validation. We utilized 5-fold cross-validation with hyperparameter tuning (e.g., learning rate and depth for XGBoost; regularization for LR). For final evaluation, an independent validation dataset (N = 120,232) from April–May 2024 was used. We performed a discordant case analysis (using t-tests and chi-square tests) to investigate instances where ML predictions differed from conventional threshold-based detection. Finally, SHAP (SHapley Additive exPlanations) values were calculated to provide model interpretability, identifying the key maternal health indicators driving the stratification.

### Reproducibility

All codes for data pre-processing, feature engineering, and model evaluation is documented on GitHub [26], to ensure transparency and reproducibility. After applying the data processing and modeling pipeline described above, we obtained the following results.

### Ethics statement

This study received ethical approval from the Institutional Review Board of the National Institute for Medical Research (NIMR), Tanzania, under approval number NIMR/HQ/R.8a/Vol.IX/4194 dated January 23 ^rd^, 2023. Informed verbal consent was obtained from all participants prior to conducting the interviews. Additional data were retrospectively sourced from records of routine antenatal care (ANC) clinic visits. All procedures were conducted in accordance with national ethical guidelines and regulations governing research involving human subjects.

## Results

### Descriptive results

**Fig 1:**
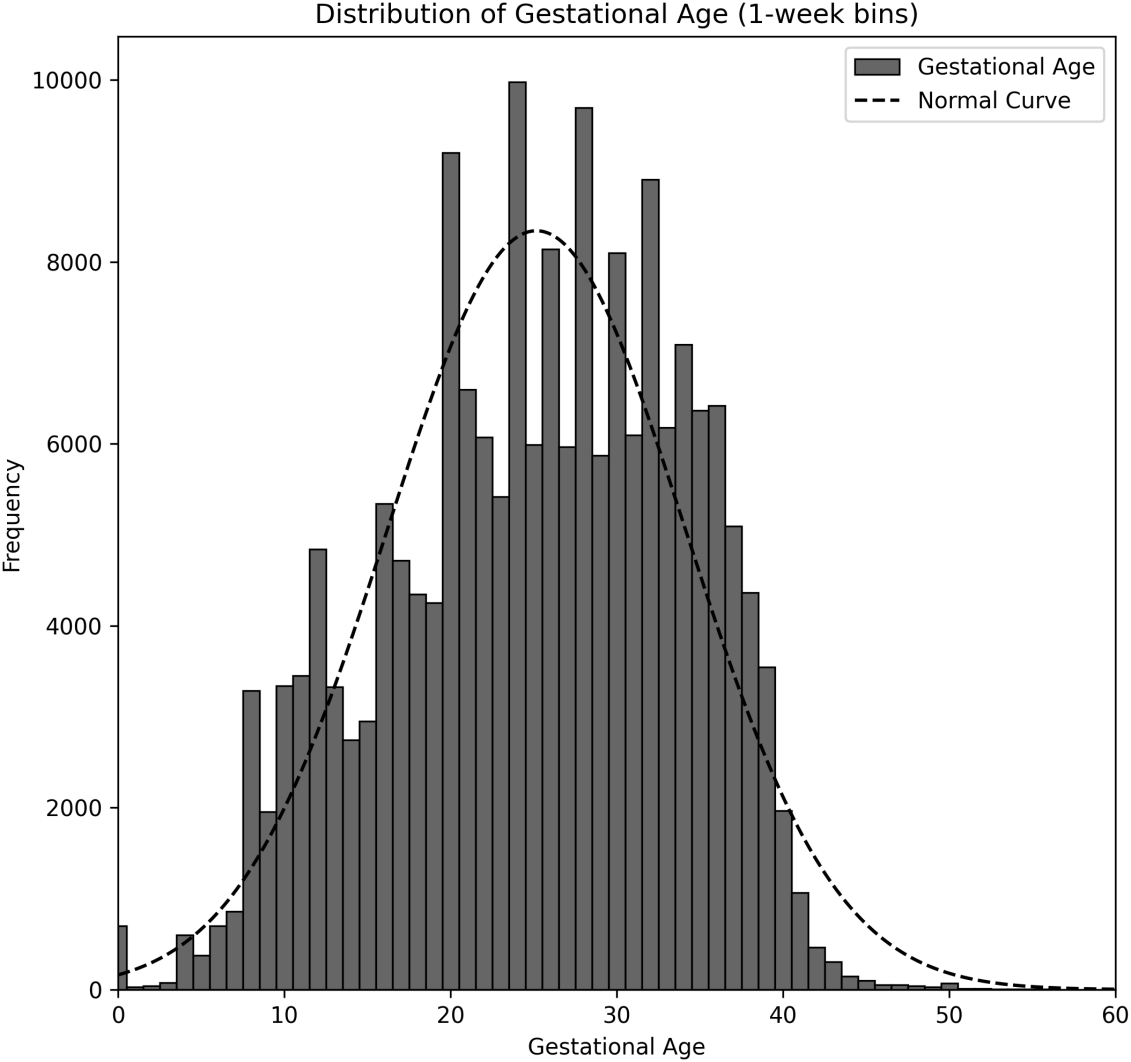
Frequency distribution by gestational age.

Between 2020 and 2024, a total of 337,027 ANC visits were recorded in the UCS. After collapsing these longitudinal entries into unique patient records, the final analytic dataset comprised 187,438 clients. The average gestational age at the time of visit was 24 weeks (Fig 1)

### Temporal data sparsity and visit frequency

The dataset is characterized by significant temporal sparsity (Table 1). The median number of ANC visits per client was 1 (IQR: 1–2). Only 43.4% (81,325/187,438) of clients had two or more recorded visits, while just 6.3% (11,795/187,438) achieved at least four visits. Critically, only 0.08% (157/187,438) of the population reached the WHO-recommended minimum of eight ANC visits. This high proportion of single-visit interactions (54% of total records) underscores the necessity of the “flattening” approach used for risk stratification.

**Table 1:** Frequency distribution of ANC clients by visit/contact number before and after collapse.

	ANC Clients (Before Collapse)			ANC Clients (After Collapse)		
Visit No.	Freq	Percent	Cum. Percent	Freq	Percent	Cum. Percent
1	182,313	54.09%	54%	80,773	43.09%	43%
2	104,886	31.12%	85%	72,448	38.65%	82%
3	33,487	9.94%	95%	22,368	11.93%	94%
4	11,606	3.44%	99%	8,247	4.40%	98%
5	3,494	1.04%	100%	2,609	1.39%	99%
6	915	0.27%	100%	732	0.39%	100%
7	202	0.06%	100%	157	0.08%	100%
8	49	0.01%	100%	33	0.02%	100%
9	17	0.01%	100%	14	0.01%	100%
10	3	0.00%	100%	2	0.00%	100%
11	1	0.00%	100%	1	0.00%	100%
*99	54	0.02%	100%	54	0.03%	100%
Total	337,027			187,438		

Note: *99 represents NULL or Missing.

#### Data quality and missingness.

Missingness rates varied significantly across clinical features (Table 2). Prior to data collapsing, variables such as height and gestational age were nearly complete. In contrast, diagnostic and laboratory features showed severe gaps: blood glucose (76% missing), Rh factor (72%), and fetal heart rate (63%) were the least documented. After collapsing visits into client-level records, a feature was only marked as missing if no value was recorded across any of that client’s available visits, providing the most complete physiological profile possible for each individual.

### Analysis of the excluded population

To assess potential selection bias, we compared the clinical characteristics of the included analytic sample (complete cases) with the excluded population (Tables 3 and 4).

**Table 3:** Comparison of numerical variables between excluded and included records.

Feature	Excluded (N)	Excluded (Mean SD)	Included (N)	Included (Mean SD)	Difference	P-Value	Test
systolic	136,864	114.8 (9.6)	50,574	115.3 (11.3)	0.5	<0.001	t-test
diastolic	136,864	69.5 (7.5)	50,574	70.2 (8.8)	0.7	<0.001	t-test

**Table 4:** Comparison of categorical variables between excluded and included records.

Feature	Category	% in dropped	% in kept	Difference.	P-Value	Test
protein_in_urine	None	17.84%	0.00%	0.178		
	negative	52.54%	95.04%	-0.425		
	positive	0.17%	0.44%	-0.003		
	test_not_conducted	29.46%	4.51%	0.249		
	Overall				<0.001	chi2
glucose_in_urine	None	17.83%	0.00%	0.178		
	negative	50.47%	95.08%	-0.446		
	positive	0.05%	0.14%	-0.001		
	test_not_conducted	31.65%	4.77%	0.269		
	Overall				<0.001	chi2
syphilis	None	7.12%	2.56%	0.046		
	negative	83.70%	96.10%	-0.124		
	positive	0.26%	0.21%	0.001		
	test_not_conducted	8.92%	1.13%	0.078		
	Overall				<0.001	chi2

#### Physiological and diagnostic comparisons.

Statistically significant but clinically minimal differences were observed in both systolic and diastolic blood pressure between the two groups (Table 3). However, the primary divergence was found in diagnostic coverage rather than physiological values. The excluded population was predominantly characterized by a systemic absence of laboratory data.

#### Diagnostic gaps (Table 4).

The disparity in testing was most pronounced for proteinuria and blood glucose:

- **Proteinuria:** 47.3% of the excluded records lacked results: either entirely missing (17.8%) or explicitly documented as “test not conducted” (29.5%) compared to only 4.5% in the included sample.
- **Blood Glucose:** Similarly, 49.4% of the excluded records lacked glucose data (17.8% missing and 31.6% “test not conducted”), whereas only 4.8% of the included sample faced these gaps.

While syphilis’ positivity rates were comparable between the groups (0.26% vs. 0.21%), the excluded population exhibited an eight-fold higher rate of “test not conducted” for syphilis (8.9% vs. 1.1%). These findings suggest that exclusions were driven primarily by resource constraints and facility-level testing gaps rather than differences in the underlying health status of the patients. This reinforces the study’s narrative that ML tools must be designed to function within the constraints of varying diagnostic availability

Tables 3 and 4 compare the 136,864 excluded records (73%) with the 50,574 included records. Excluded women had significantly higher rates of missing proteinuria (47.3% vs. 4.5%) and glucose testing (49.4% vs. 4.8%), suggesting systematic undertesting rather than random missingness.

## Feature selection and model development

### Bivariate analysis and statistical screening

**Fig 2:**
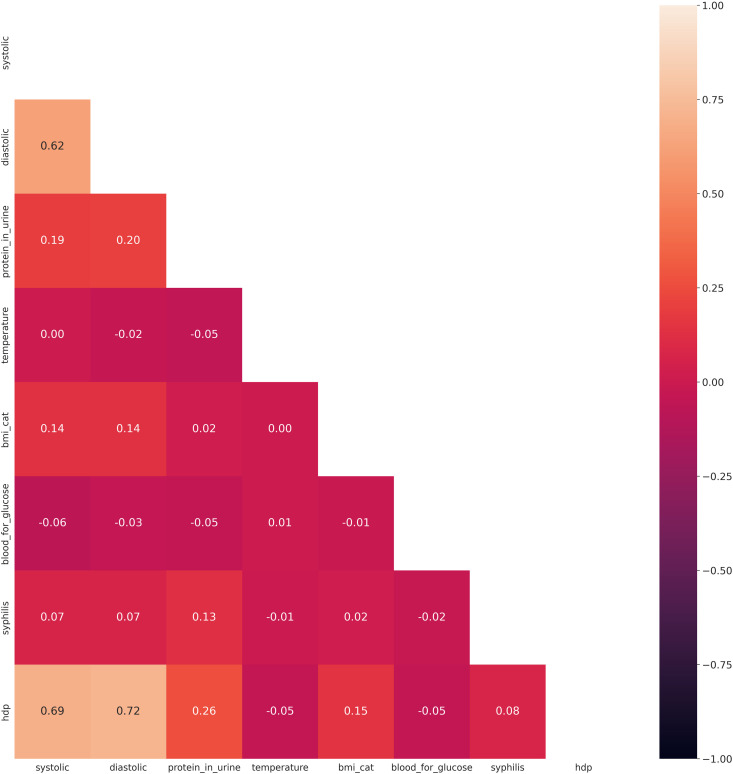
Heatmap showing correction of selected variables. Pearson correlation coefficients are displayed; linearity assumptions were visually confirmed through scatterplot inspection and supported by consistent Spearman’s rank correlations (see Methods).

Initial feature selection was guided by data availability and clinical expertise. Bivariate analysis using Pearson correlation showed strong positive associations between HDP and systolic blood pressure (r = 0.69, p < 0.05) and diastolic blood pressure (r = 0.72, p < 0.05) (Fig 2). Spearman’s rank correlation yielded consistent results (SBP: ρ = 0.67; DBP: ρ = 0.71), confirming the robustness of these primary predictors. Categorical screening via Chi-square tests identified proteinuria (χ² = 1377.02, Cramer’s V = 0.166) and BMI categories (χ² = 1087.67, Cramer’s V = 0.147) as the strongest non-continuous predictors. Mutual Information (MI) scores further confirmed that SBP and DBP were the most informative features, while temperature (MI = 0.008) and syphilis status (MI = 0.001) provided minimal individual predictive gains.

### Final analytic dataset construction

From the initial 187,438 client records, 136,864 (73%) were excluded due to the missingness in key features or physiological outliers (>3 SD), resulting in a final analytic dataset of 50,574 records. This sample maintained a natural HDP prevalence of 3.7% (1,894 cases). As detailed in the Methods, a random under-sampling procedure was applied to the training split to create a balanced set of 3,788 records. The final model incorporated seven clinically relevant predictors: SBP, DBP, BMI, blood glucose, proteinuria, temperature, and syphilis.

### Model selection and training

**Fig 3:**
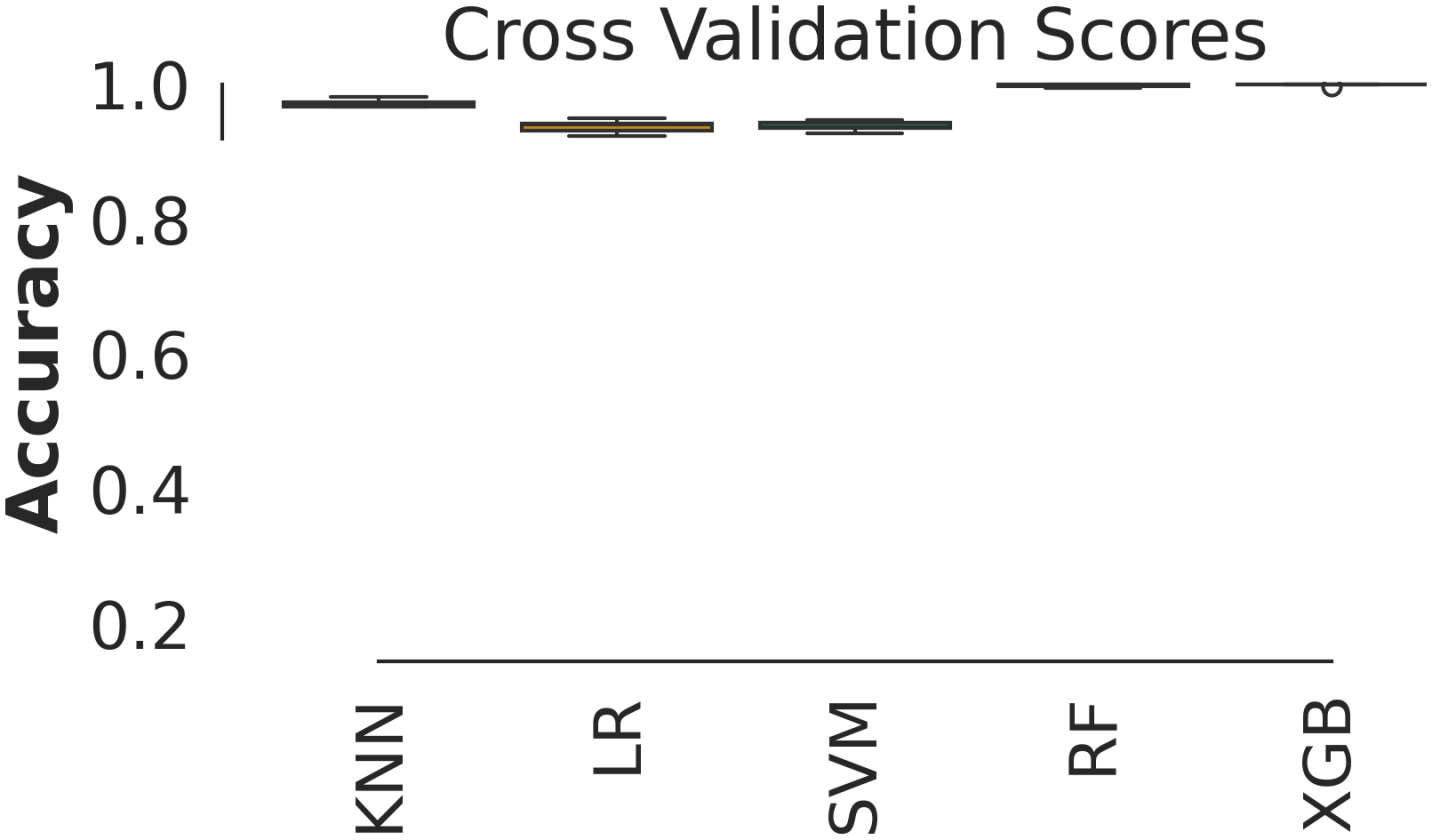
Cross validation results.

**Fig 4:**
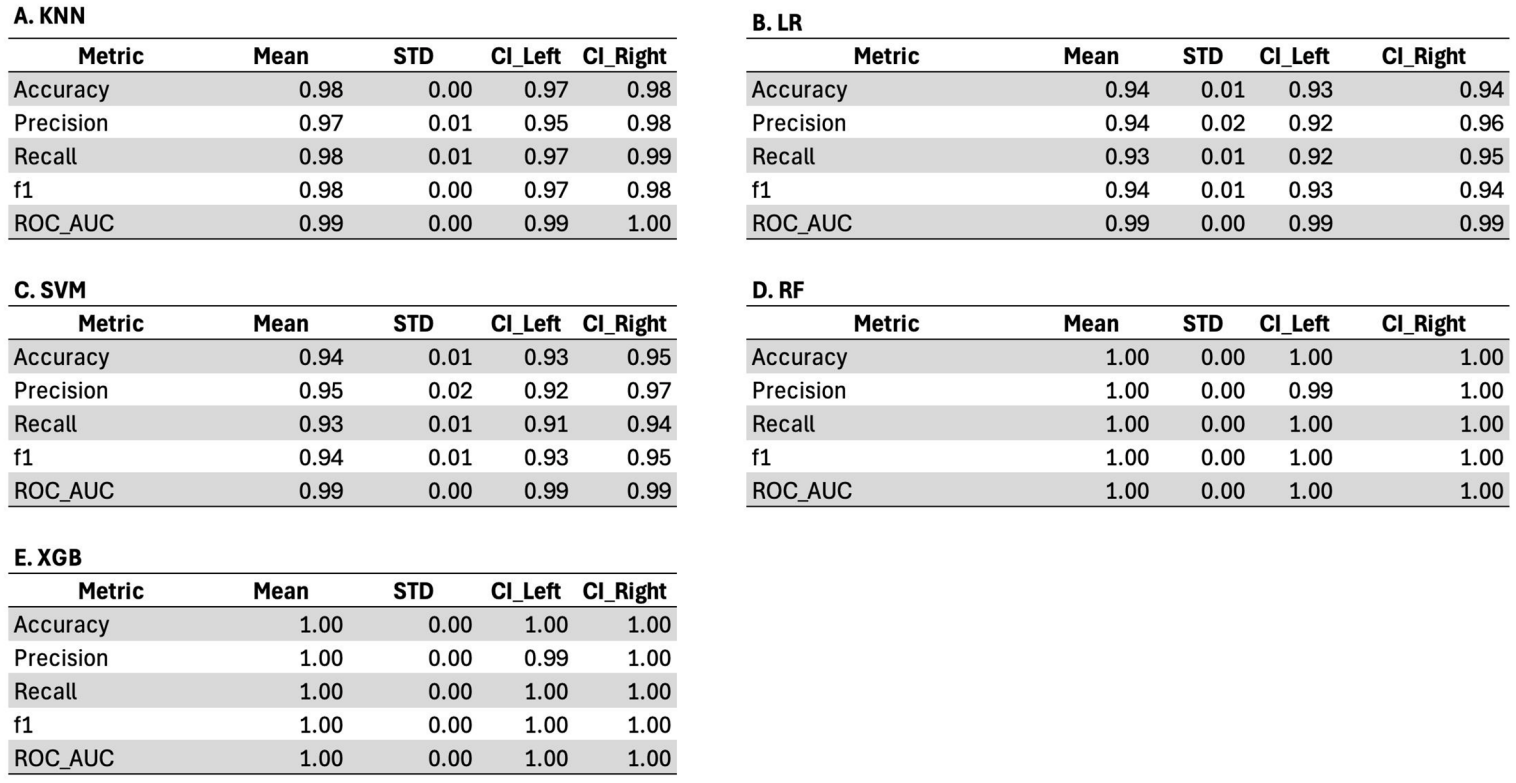
Classification report.

We evaluated five ML algorithms using 5-fold cross-validation on the balanced training set (Fig 3). All models demonstrated high accuracy, with Random Forest (99.79%) and XGBoost (99.91%) achieving near-perfect performance across all metrics (Accuracy, Precision, F1, and AUC = 1.00) with zero variance on the cleaned training data (Fig 4). Based on this superior performance and its known robustness in handling the non-linear complexities of routine health data, XGBoost was selected as the optimal model for final deployment and validation.

### Model interpretability via SHAP analysis

**Fig 5:**
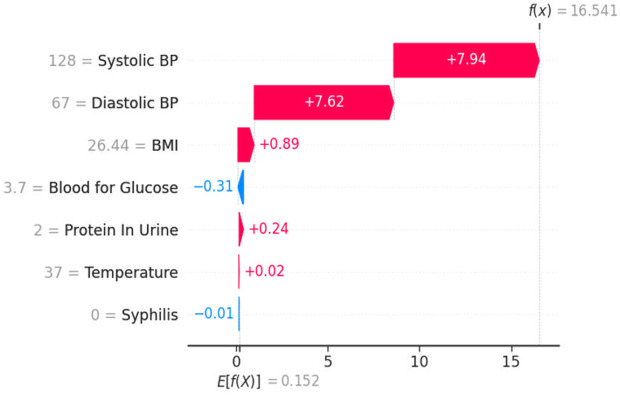
SHAP feature importance on model output.

To look beyond global performance and understand individual feature contributions, we employed SHAP (SHapley Additive exPlanations) (Fig 5). The base value (E[f(x)] = 0.152) represents the model’s expected output before accounting for specific client features.

- **Primary Drivers:** SBP and DBP dominated the model’s decision-making (mean SHAP +7.94 and +7.62, respectively), where higher values (indicated in red) significantly increased the predicted risk of HDP.
- **Secondary Predictors:** BMI (+0.89) and proteinuria (+0.24) provided modest but clinically meaningful contributions.
- **Systemic Utility:** While blood glucose, temperature, and syphilis status contributed minimally to the overall prediction, they were retained in the final model to ensure a comprehensive biological profile and to maintain the tool’s utility across diverse clinical presentations in the UCS system.

### Model validation and performance on real-world data

#### Independent validation performance.

**Fig 6:**
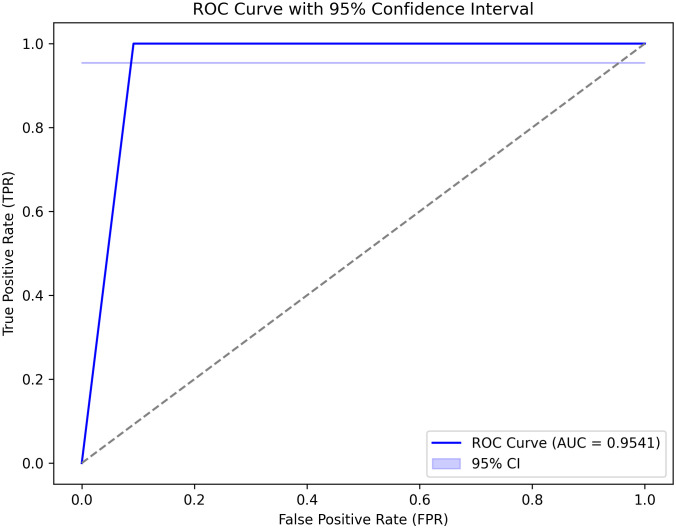
Receiver operating characteristics.

The XGBoost model was validated on an independent dataset of 120,232 new records (April–May 2024), maintaining a natural HDP prevalence of 1.4%. As shown in Table 5, the model achieved an overall accuracy of 90.95% (95% CI: 90.8%–91.1%) and an AUC of 0.95 (95% CI: 0.953–0.955), demonstrating exceptionally discriminating power (Fig 6). While cross-validation on balanced data yielded near-perfect precision, the transition to real-world data resulted in a precision of 0.14 (95% CI: 0.130–0.143) and an F1-score of 0.24. This reflects the high-volume, low-prevalence nature of routine health systems and our deliberate calibration toward patient safety.

**Table 5:** Performance metrics and classification report for HDP risk stratification (N = 120,232).

Metric/Class	Precision (95% CI)	Recall/Sens (95% CI)	F1-Score (95% CI)	Support (N)
No Risk	1 (-)	0.91 (0.906 - 0.910)*	0.95 (-)	118,507
Risk	0.14 (0.130 - 0.143)	1.00 (1.00 - 1.00)	0.24 (0.231 - 0.250)	1,725
Overall Model	AUC: 0.95 (0.953 - 0.955)	Accuracy: 0.91 (0.908 - 0.911)	PPR: 0.10 (0.103 - 0.107)	Total: 120,232

* Specificity is represented by the recall of the “No Risk” class.

#### Clinical threshold optimization and triage utility.

**Fig 7:**
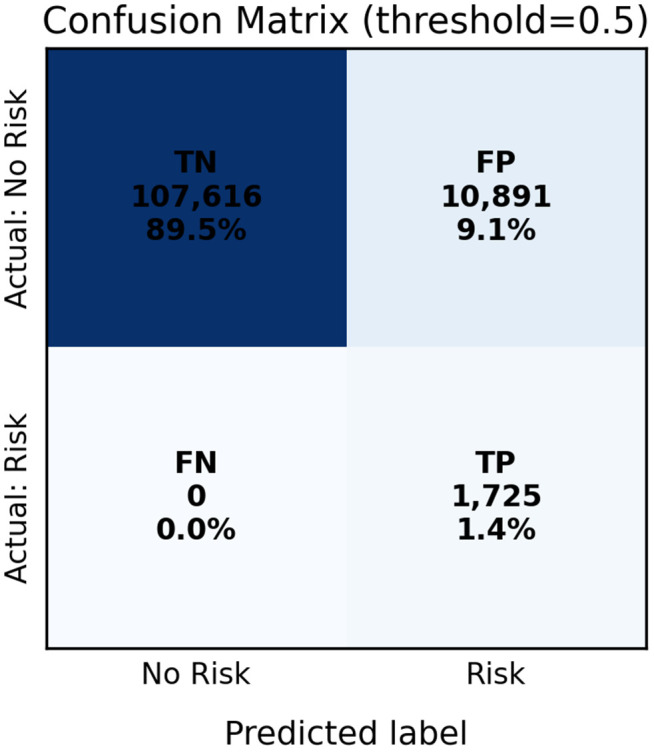
Confusion matrix.

Standard optimization (e.g., F1-score maximization) suggested a high probability threshold (0.995). However, this would have identified only 40 of 1,725 true HDP cases (2% sensitivity), which was deemed clinically unacceptable. By selecting a decision threshold that prioritizes 100% Sensitivity (Recall), the model successfully identified all 1,725 true HDP cases in the validation set (Fig 7). This strategy resulted in a Positive Predictive Rate (PPR) of 0.10, flagging a total of 12,615 records for clinical review. While this introduces a higher volume of false positives (Specificity: 0.91), it ensures that in resource-constrained clinics, the “screen-in” mechanism captures every potential case for manual blood pressure confirmation.

#### Precision-Recall and calibration analysis.

**Fig 8:**
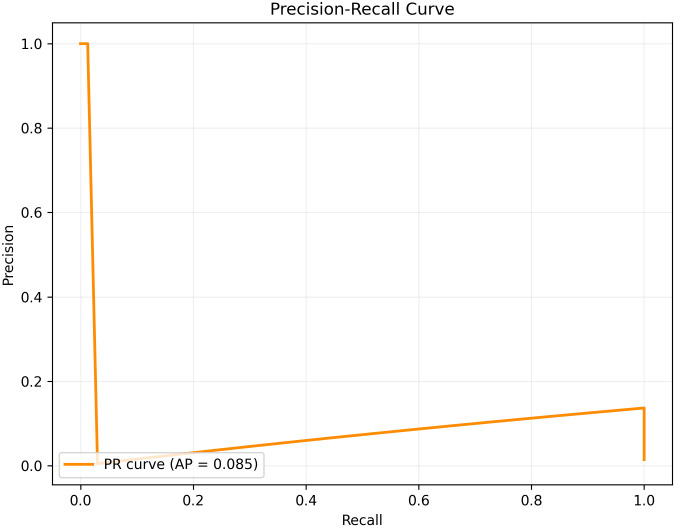
Precision recall curve.

**Fig 9:**
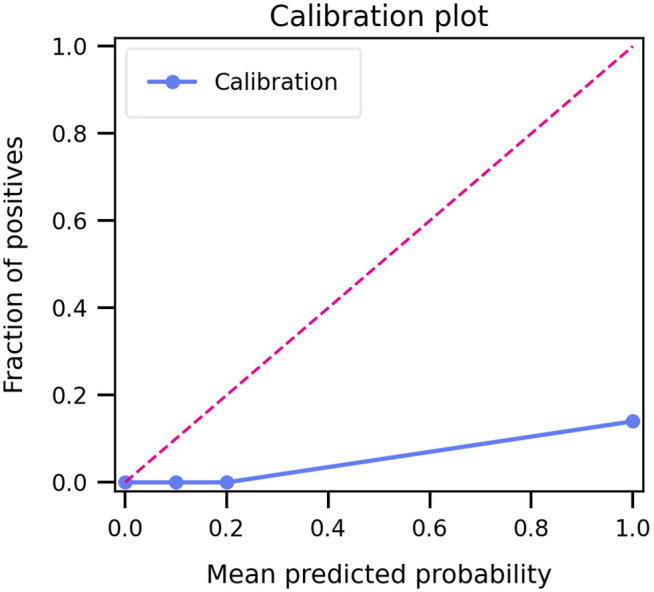
Calibration plot.

The Precision-Recall curve (Fig 8) yielded an Average Precision (AP) of 0.169, which significantly outperformed the no-skill baseline (0.014), indicating a 12-fold improvement in identifying risk cases compared to random chance in this specific validation cohort. The Calibration Plot (Fig 9) and a Brier score of 0.08 confirmed a systematic tendency to overpredict risk, an intentional byproduct of our “safety-first” thresholding designed to minimize the catastrophic risk of missed hypertensive disorders.

#### Discordant case analysis.

**Fig 10:**
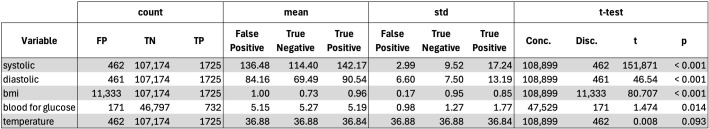
Analysis of discordant pairs (numeric variables).

To understand the nature of the 10,878 false positives, we compared them against true negative records (Fig 10). The analysis revealed that these “false positives” were not random errors; they were clients with significantly elevated physiological markers compared to the healthy population:

- Blood Pressure: False positives had a mean SBP of 136.5 mmHg and DBP of 84.2 mmHg, compared to 114.4/69.5 mmHg in true negatives (p < 0.001).
- Clinical Features: BMI, proteinuria, and syphilis status also showed significant associations (p < 0.001) with being flagged as “at-risk.”

This suggests that the model is capable in identifying a “sub-clinical” high-risk group, i.e., women who do not yet meet the 140/90 mmHg diagnosis threshold but exhibit physiological profiles that warrant closer clinical monitoring.

## Discussion

ML offers a practical path from single-point blood pressure (BP) thresholding toward automated, data-driven risk stratification in overburdened antenatal care (ANC) settings. Using 337,027 routine UCS records aggregated into 187,438 client-level records, our study demonstrates that an XGBoost model can achieve strong discrimination for HDP (AUC = 0.95) while maintaining maximal sensitivity (100%) on independent validation thereby operationalizing the screening priority of “do not miss” high-risk clients in a context where HDP accounts for ~34% of direct obstetric deaths in Tanzania [2].

### Data quality and missingness

Routine ANC data in low-resource settings remain imperfect: paper-based collection, fragmented recording practices, and limited diagnostic testing lead to systematic gaps [11,12,27,28]. In our cohort, missingness was not random: nearly half of excluded women lacked entire classes of clinical tests (proteinuria, glucose), reflecting either test unavailability or clinically appropriate prioritization of urgent care (e.g., late presenters, active labor). This selection mechanism means the most acutely vulnerable women may be underrepresented in training data, producing selection bias and potential inequities in model performance. Improving front-line digitization (tablets, embedded forms) and workforce training would both increase data completeness and improve model generalizability, although cost and maintenance barriers remain [29].

### Performance trade-offs and operational implications

XGBoost produced 90.1% accuracy with AUC 0.95 on validation, stratifying 12,603 clients as high-risk; 1,725 (14%) of these met conventional BP-based HDP criteria. The deliberate choice to preserve 100% sensitivity yields a precision of 14% (≈6.3 false positives per true positive). This high-sensitivity design aligns with clinical aims in Tanzania but imposes operational costs: an estimated 6.3 false positive per true postivie in the validation sample, potentially translating to dozens of extra workups per clinician per day in busy clinics [16,23]. Each false positive consumes clinician time, may provoke patient anxiety, and risks alert fatigue with downstream consequences for care quality.

### Model validity vs. real-world performance

The divergence between near-perfect development cross-validation and realistic validation performance highlights a familiar translation issue: extensively preprocessed, balanced training sets inflate apparent performance relative to noisy routine data [9,27,30]. Calibration analysis showed systematic overprediction in the validation context; the Brier score (0.08) must be interpreted against low disease prevalence and class imbalance. Thus, the model is best suited for relative risk ranking (triage) rather than precise absolute risk estimates for individual decision-making.

### Clinical interpretability and potential added value

SBP and DBP were dominant predictors, consistent with clinical knowledge and explaining the rapid early ROC gains. Similar ML studies have shown that SBP and DBP metrics are among the most influential predictors in models of hypertension and cardiovascular risk [31]. Yet the model also integrated other routinely recorded variables (proteinuria, syphilis status, etc.), producing discordant cases where elevated-but-subthreshold BP combined with ancillary signals led to high-risk classification. ML models are well suited to detect such nonlinear interactions between variables that may not be captured by traditional threshold-based clinical rules [32]. These discordant cases may represent subclinical or emergent risk states that single-point BP thresholds miss, but prospective outcome-anchored validation is required to determine whether they reflect true risk or data noise.

### Implementation strategies to mitigate false-positive burden

To translate a high-sensitivity model into workable practice, several pragmatic strategies are recommended:

- Tiered risk outputs (low/moderate/high) to prioritize immediate review for the highest-risk subgroup.
- Local workflow integration: same-day nurse recheck, in-clinic BP re-measurement protocols, or low-threshold confirmatory checks rather than automatic specialist referral.
- Use flags to trigger targeted counseling/self-monitoring and expedited follow-up rather than default high-resource pathways.
- Continuous local recalibration and monitoring as more labelled outcomes accrue to improve precision.
- Hybrid workflows combining automated triage with brief in-clinic confirmatory steps to reduce unnecessary downstream referrals.

Each mitigation requires prospective piloting (time-motion, cost-effectiveness, and acceptability studies) to quantify whether the net clinical benefit of increased sensitivity outweighs operational costs in routine Tanzanian ANC.

## Limitations

Key limitations include selection bias from non-random missingness excluding clinically important subgroups; reliance on routine BP recordings rather than standardized outcome adjudication; limited temporal depth that prevented sequential/time-series modeling; and the operational uncertainty of the false-positive workload estimates, which must be validated in real-world pilots. Heterogeneity in facility infrastructure and staffing across Tanzania will also affect implementation feasibility.

## Conclusion and recommendation

This work provides proof-of-concept that routine UCS ANC data can power ML-driven triage for HDP with near-zero missed cases, potentially improving prioritization in congested clinics and directing scarce specialist time to the most vulnerable patients. However, the substantial false-positive rate requires implementation approaches that prioritize graded triage, workflow embedding, low-cost confirmatory checks, and prospective recalibration. We recommend Ministry of Health investment in stronger digital data integration, targeted data-quality interventions, and phased pilot deployments to evaluate clinical impact, resource implications, and equity before scale-up dnext steps

**Key messages**

**What is already known on this topic**

Maternal mortality in low‑resource settings is driven by hypertensive disorders of pregnancy, yet routine ANC often relies on single blood pressure checks that miss high‑risk cases. Effective risk stratification tools are urgently needed to guide timely interventions.

**What this study adds**

Using routine ANC data from Tanzania, this study shows that ML can deliver automated risk stratification with 100% sensitivity, ensuring no high‑risk client is missed. The model functions as a practical screening tool to prioritize care in overburdened clinics.

**How this study might affect research, practice, or policy**

ML‑driven risk stratification could streamline ANC workflows by directing limited specialist time to the most vulnerable patients. Strengthening digital health infrastructure and evaluating real‑world implementation are critical next steps for policy and practice.
