# Supplementary material for: Machine learning for risk stratification of hypertensive disorders of pregnancy: Enhancing clinical efficiency in low-resource antenatal care in Tanzania
Source: PLOS Digit Health. 2026 Jul 2;5(7):e0001468. doi: 10.1371/journal.pdig.0001468 (PMC13327181; doi:10.1371/journal.pdig.0001468)
Supplement: Attachment — Submitted filename: PDIG-D-25-00485R1_reviewer_JULIAN_BORGES.pdf [file pdig.0001468.s004.pdf]

Title: *Harnessing Digital Health and Machine Learning for Early Detection of Hypertensive Disorders of Pregnancy: Insights from Low-Resource Settings, Tanzania*

PDIG-D-25-00485R1_reviewer_JULIAN_BORGES

Summary

The authors present a retrospective study applying machine learning (ML) to antenatal care (ANC) data collected from Tanzania's Unified Community System (UCS) to predict hypertensive disorders of pregnancy (HDP). They evaluate several classifiers, selecting XGBoost for deployment, reporting an AUC of 0.95 and 90.95% accuracy on unseen data (n >120,000). The goal is to demonstrate how digital health data can support early detection of HDP in low-resource environments.

Both prior reviewers agree that the paper demonstrates relevance, ambition, and technical effort, but also exhibits substantial limitations in clinical labeling, model evaluation, temporal data usage, and real-world implementation claims. This final review consolidates those points, offering a full technical evaluation.

Major Issues

1. Use of Proxy Outcome Definition (HDP) Without Clinical Validation
   The outcome label for HDP is derived solely from systolic and diastolic blood pressure thresholds (≥140/90 mmHg), without confirmation from diagnosis fields, medication data, or clinician annotations. This label construction is acknowledged as a proxy but remains fundamentally weak for building clinically trustworthy prediction models.
   Impact: This creates a risk of label noise, undermines model evaluation, and raises concerns about internal validity.
   Recommendation: While data limitations are acknowledged, the paper must reframe its scope from "early detection of HDP" to "classification of blood pressure risk states," and clearly state the limits of proxy labeling in both methods and conclusions.
2. Severe Precision Deficit (14%) With Limited Operational Mitigation
   Both reviews noted that the model exhibits a 14% precision—indicating that most positive predictions are false positives. The authors attempt to justify this through high recall and potential for early warning, but there is no quantitative or operational analysis of the downstream clinical impact of such a high false positive burden in resource-constrained settings.
   Impact: This challenges the model's practical deployability and user trust.
   Recommendation: Authors must include a risk-benefit discussion, propose specific triaging protocols, or demonstrate via simulation how false positives would be handled without overwhelming staff or infrastructure.

3. Flattening of Temporal Data is a Structural Limitation
   The decision to aggregate longitudinal ANC visit data into static per-patient feature vectors eliminates critical gestational progression patterns, which are central to HDP pathophysiology. This limitation is acknowledged but not sufficiently addressed.
   Impact: This structurally limits predictive accuracy and masks important trends such as rising BP trajectories or changes in proteinuria over time.
   Recommendation: Authors should either experiment with temporally-aware models or provide structured temporal feature engineering (e.g., BP slopes, deltas), and fully articulate the implications of the current model's static nature.
4. Lack of External or Prospective Validation
   While the model is tested on a large test cohort (n = 120,232), no prospective, geographic, or temporal external validation is performed. Furthermore, predictions are not linked to actual delivery or maternal outcomes.
   Impact: Limits generalizability and weakens claims about readiness for deployment or policy use.
   Recommendation: The manuscript should temper claims regarding deployment and emphasize the need for a formal prospective validation study. A pilot phase or follow-up outcome analysis would significantly strengthen future versions.
5. Omission of Missing Data and Data Quality Protocols
   There is no description of missing data handling, which is critical in LMIC digital health datasets. Features such as glucose, protein in urine, and syphilis status are often missing or inconsistently recorded.
   Impact: Compromises the transparency, reproducibility, and robustness of the model.
   Recommendation: Authors must report missingness rates per feature, describe imputation or exclusion methods, and assess model sensitivity to data completeness.

Minor Issues

- Model threshold selection and calibration are not discussed. Threshold optimization (e.g., Youden Index, F1-maximization) under class imbalance should be detailed.
- Feature definitions are ambiguous in several places (e.g., "ANC risk flag," "partner HIV status"); authors should provide a data dictionary or glossary.
- Use of ROC curves is insufficient under high class imbalance; precision-recall curves should be included.
- SHAP plots are informative but need clearer axis labels and clinical interpretation guidance.
- Terminological ambiguity between "HDP" and "gestational hypertension" must be resolved; either clarify or limit the scope of what the model is predicting.

Figures and Tables

Figures are well constructed but need improvements in labeling. A confusion matrix and precision-recall curve should be included. Tables summarizing missing data rates and class distribution across training, validation, and test sets are missing and should be provided.

Methods

Structurally adequate but lacking in key details:

- No account of data cleaning or outlier handling
- No missing data methodology
- No description of feature engineering or variable encoding
- Sampling strategy for balancing classes is not defined

Study Design

Appropriate as a retrospective feasibility study. However, the title and framing imply implementation readiness, which the current evidence does not support. A prospective or hybrid study design would be a more suitable next step.

Any Experiments Missing?

- External or temporal validation experiment
- Sensitivity analysis of label definitions
- Subgroup analysis by gestational age or risk tier
- Threshold tuning experiment

Statistics

Baseline metrics are acceptable, but calibration (e.g., Brier score, reliability diagrams) is not assessed. Confidence intervals for key metrics (AUC, precision, recall) are missing.

Results and Data: Support Conclusions?

Partially. The technical performance is adequate, but the clinical conclusions and implementation claims outpace the underlying evidence.

All Data Accessible?

No data or code sharing statement is present. Authors must clarify availability of de-identified data and model artifacts for reproducibility.

References

Sufficient for background and methodology. Authors may consider referencing clinical HDP prediction tools (e.g., fullPIERS, PREP-S) and ethical implications of ML in maternal health.

Title

Accurate but overstated. Given the limitations in clinical outcome validation, a more accurate title would replace “Early Detection of Hypertensive Disorders of Pregnancy” with “Machine Learning Risk Classification of Elevated Blood Pressure in ANC Records.”
