# Supplementary material for: Machine learning for risk stratification of hypertensive disorders of pregnancy: Enhancing clinical efficiency in low-resource antenatal care in Tanzania
Source: PLOS Digit Health. 2026 Jul 2;5(7):e0001468. doi: 10.1371/journal.pdig.0001468 (PMC13327181; doi:10.1371/journal.pdig.0001468)
Supplement: Attachment — Submitted filename: PDIG-D-25-00485_reviewer_JULIAN_BORGES.pdf [file pdig.0001468.s007.pdf]

Field	Value
Manuscript ID	PDIG-D-25-00485
Journal	PLOS Digital Health
Article Type	Research Article
Title	Machine Learning for Risk Stratification of Hypertensive Disorders of Pregnancy: Enhancing Clinical Efficiency in low-resource Antenatal Care in Tanzania
Corresponding Author	Isaac Lyatuu — Ifakara Health Institute, Dar-es-Salaam, Tanzania
Review Date	April 2026
Review Round	R3

1. Manuscript Overview

Element	Detail
Study design	Retrospective cohort study using routine ANC records from Tanzania's Unified Community System (UCS); R2 submission following two prior rounds of major revision
Dataset	337,027 ANC visit records (2023–2024) collapsed to 187,438 unique client records; 50,574 clean records post-ETL; analytic dataset after listwise deletion: 50,574 minus 136,864 excluded = approximately 50,574 (authors report 73% exclusion from 187,438)
Outcome definition	BP-defined HDP: systolic BP ≥140 mmHg or diastolic BP ≥90 mmHg; 1,894 HDP cases identified; no clinically confirmed diagnoses available
Model selected	XGBoost; balanced training set n=3,788 (1,894 cases + 1,894 controls via random undersampling); independent temporal validation n=120,232
Key performance metrics	Training (balanced): accuracy 100%, AUC 1.00 (acknowledged as artifact); Validation: accuracy 90.95%, AUC 0.95, sensitivity 100%, precision 14%, F1 24%; confusion matrix: TP=1,725, FP=10,878, TN=107,629, FN=0
R2 major additions	Calibration plot (Figure 9), precision-recall curve (Figure 8), Brier score (0.08), missing data table (Table 2), discordant case analysis, operational burden quantification (6.3 FP per TP), reframing from "early detection" to "risk stratification", data leakage subsection, SMOTE mention, threshold sensitivity paragraph, comparison with fullPIERS/PIERS-ML
Funder	Bill & Melinda Gates Foundation (Grand Challenges, INV-046249) to Prime Health Initiative Tanzania (PHIT); funder had no role in study design or reporting

2. Summary of Review

This is the third round of review for a research article applying machine learning to ANC data from Tanzania's Unified Community System for risk stratification of hypertensive disorders of pregnancy. The R2 revision is the most substantive to date: the authors have reframed the manuscript from "early detection" to "risk stratification," added a data leakage subsection, provided a missing data table, included calibration and precision-recall analyses, quantified the operational false-positive burden, added comparison with established HDP prediction models (fullPIERS, PIERS-ML, Li et al., Chen and Ma), and substantially expanded the limitations section. Two prior reviewers (Reviewers 2 and 3) characterised the revision

positively, with Reviewer 2 upgrading their recommendation to minor revision. Reviewer 4 requested major revision, and Reviewer 5 recommended reject; both raised overlapping concerns that are now substantially, though not completely, addressed.

At R3, the manuscript stands materially closer to acceptance. One critical issue remains that was not resolved in R2: the 73% exclusion of records due to listwise deletion is quantified and partially described in Table 2, but the systematic difference analysis between included and excluded records, which the authors describe performing (t-tests and chi-square with $p < 0.001$ across comparisons), does not appear as a formal table in the manuscript and the implications for deployment representativeness are not fully drawn. This constitutes a Critical issue because the analytic cohort represents a minority of the target population, and the authors' own analysis demonstrates the exclusions are not random. Two Major issues and three Moderate issues are also identified.

Dimension	R3 Assessment
Conceptual framing	Substantially improved: "risk stratification" and "screening support" language is now consistent; epistemic boundary is appropriately stated
Data leakage disclosure	Addressed: dedicated subsection added; circular reasoning acknowledged; model reframed as BP-pattern detection, not clinical HDP prediction
Missing data handling	Partially addressed: Table 2 added; exclusion comparison analysis described but not formally presented as a table in the manuscript body
Temporal data limitation	Addressed with quantitative justification (median visits=1, IQR 1-2; only 6.3% with ≥4 visits); structural constraint is now transparently documented
Precision-recall and calibration	Addressed: PR curve (Fig 8), calibration plot (Fig 9), Brier score (0.08) added
Operational burden	Addressed: 6.3 FP per TP quantified; clinical workflow framing as triage tool added
Comparison with prior models	Addressed: fullPIERS, PIERS-ML, Li et al., Chen and Ma incorporated; contextualization is adequate
Overall recommendation	Minor Revision — one Critical issue requires author response; two Major issues require targeted correction

3. Critical and Major Issues

3.1 Critical Issues

Issue ID	C1 | Critical
Title	Systematic Exclusion of 73% of Records Is Described but Not Formally Presented as a Comparative Table in the Manuscript
Description	The authors excluded 136,864 of 187,438 client records (73%) due to listwise deletion after feature engineering. This exclusion rate is critical to the validity of any deployment inference because the analytic cohort is a small and, by the authors' own analysis, systematically different minority of the target population. The authors state in their response to Reviewer 5 that they performed t-tests and chi-square tests comparing excluded versus included records and found "highly significant differences ($p < 0.001$ for all comparisons)," with systematic undertesting in excluded women (nearly half lacking proteinuria and glucose testing). They further acknowledge this likely reflects women presenting urgently, arriving late, or experiencing complications — precisely the highest-risk subgroup for HDP. This analysis is described in the response letter but is not presented as a formal table in the manuscript body. Without this table, readers cannot

	evaluate the representativeness of the analytic cohort. The issue is not that 73% were excluded — that is a documented data quality constraint — but that the comparison analysis exists and is not shown, meaning the manuscript currently conceals information that the authors possess and that materially affects interpretation of the model's deployment utility.
**Recommendation**	Present the comparison of excluded versus included records as a formal supplementary or main-text table with statistical test results (t-tests for continuous variables, chi-square for categorical) and p-values as described in the response letter. Add a paragraph in the Discussion that draws the implication explicitly: the model was trained and validated on the 27% of clients with complete feature records, who systematically differ from excluded clients; deployment to the full ANC population would encounter a substantially different data completeness profile, and model performance in that population is unknown. A one-sentence recommendation for prospective data quality improvement as a prerequisite for broader deployment should be included.

3.2 Major Issue: Near-Perfect Training Metrics Remain Inadequately Explained in Results Section

**Issue ID**	M1 | Major
**Title**	Table 2 Training Performance (100% Accuracy, AUC 1.00) Is Still Not Sufficiently Contextualized in the Results Narrative
**Description**	The authors acknowledge in their response to Reviewer 2 (R2) that near-perfect cross-validation metrics on the balanced training set are "an artifact of our methodological choices — specifically random under-sampling to achieve class balance and the algorithmic outcome definition." This is the correct explanation and it should be stated explicitly in the Results section itself. Reviewer 2 at R2 specifically requested that the manuscript "clearly say that these results do not prove the model is better" and the authors agreed. However, the pattern of agreement in response letters without corresponding change in manuscript text has recurred across review rounds for this specific point. At R3, the concern is not about the quality of the explanation (which is adequate) but about whether it is in the manuscript rather than only in response letters. If Table 2 still presents 100% metrics without a co-located caveat in the manuscript text, this remains unresolved.
**Recommendation**	Add a sentence directly adjacent to or within the Table 2 caption or the corresponding results paragraph that states: (a) the training performance reflects an artificially balanced, algorithmically labelled dataset and should not be interpreted as evidence of generalizability; (b) the discrepancy between training and validation metrics is expected given the study design; and (c) the validation metrics (14% precision, 90.95% accuracy, AUC 0.95) are the operationally meaningful figures. This is a text placement correction, not a new analysis.

3.3 Major Issue: SMOTE vs. Random Undersampling Inconsistency Between Response Letters

**Issue ID**	M2 | Major
**Title**	Response Letters Cite SMOTE Oversampling While Manuscript Text Describes Random Undersampling — Method Is Inconsistently Reported
**Description**	The response to Reviewer 2 (Methods section) states: "Class imbalance handling through SMOTE oversampling in the training set." The response to Reviewer 5 states: "we employed random undersampling of the majority class to create a balanced training set of 3,788 records (1,894 cases + 1,894 controls)." The confusion matrix confirms n=3,788 training records, which is consistent with undersampling (duplicating the minority class to 3,788 total is SMOTE; reducing the majority to 1,894 is undersampling). The actual method used has material consequences for model evaluation: SMOTE generates synthetic minority-class instances and can introduce distributional artifacts, while random undersampling discards majority-class information. The reviewer cannot

	determine from the response letters which method was actually implemented, and the inconsistency must be resolved in the manuscript before publication.
**Recommendation**	State the class balancing method once, precisely, in the Methods section: specify whether random undersampling, SMOTE, or a combination was used; state the resulting training set composition (n=3,788 with class distribution); and remove any inconsistent reference in the manuscript or response letters. If both methods were applied at different stages of model development, describe each stage separately with sample counts.

4. Moderate Issues

4.1 Study Design: Listwise Deletion Rationale Requires Formal Missingness Analysis Framing

**Issue ID**	Mod1 | Study Design
**Title**	Complete-Case Analysis Rationale Does Not Address Missing-Not-At-Random Implications for Model Deployment
**Domain**	Study Design
**Description**	The authors justify listwise deletion by stating that imputation could introduce bias in a MNAR (missing not at random) setting. This is a reasonable position, but the conclusion drawn from it — that complete-case analysis is therefore appropriate — requires an additional step: the authors must demonstrate or argue that the resulting analytic cohort is still a representative sample for the intended use case. The comparison table (C1 above) partially addresses this, but even with that table, the Discussion should explicitly address what MNAR missingness implies for model deployment: if data are missing because women are sicker or have less access to testing, a model trained on complete cases will underperform precisely on the women at highest risk. This is an implementation science finding, not merely a statistical limitation, and should be framed as such.
**Recommendation**	Add one paragraph in the Discussion that explicitly characterises the missingness mechanism (MNAR, supported by the comparison analysis), states the implication for model deployment in a setting where data completeness varies by patient risk profile, and recommends a data quality intervention (standardised proteinuria and glucose testing at first ANC visit) as a prerequisite for deployment equity.

4.2 External Validity: Temporal Validation Cohort Originated From Same Facilities as Training Data

**Issue ID**	Mod2 | External Validity
**Title**	The "Independent" Validation Dataset Shares Facility Origins With the Training Dataset — Geographic Generalizability Remains Unassessed
**Domain**	External Validity
**Description**	The authors clarify that "the temporal validation dataset, while independent, came from the same health facilities and regions as the training data." This means the validation assesses temporal stability within the same facility network, not geographic generalizability. The performance figures (AUC 0.95, precision 14%) cannot be extrapolated to facilities not represented in the training set. This is stated in the limitations but not prominently enough given that the policy recommendation in the manuscript (Ministry of Health deployment) implies nationwide scaling, not facility-network scaling. The current framing overstates the scope of evidence for deployment.

**Recommendation**	Revise the interpretation of validation results to explicitly limit generalizability claims to the UCS facility network represented in the dataset. In the recommendations section, introduce geographic external validation as a prerequisite step before Ministry-level scaling, with specific reference to the need for testing in rural or low-connectivity facilities not currently in the UCS implementation coverage.

4.3 Confounding and Bias: Discordant Case Analysis Interpretation Overstates Added Value

**Issue ID**	Mod3 | Confounding and Bias
**Title**	Discordant Case Analysis Shows Mean Systolic BP of 136.5 mmHg in False Positives — This Is Borderline Detection, Not Early Warning
**Domain**	Confounding and Bias
**Description**	Reviewer 5 at R2 noted that the discordant case analysis shows false positives have mean systolic BP of 136.5 mmHg versus the 140 mmHg threshold — a 3.5 mmHg difference — and characterised this as "borderline detection rather than early warning." The authors accepted this framing and reframed the paper accordingly. However, the manuscript should now explicitly present this figure (136.5 mmHg) in the Results and use it to define what the model actually adds: it flags women just below the diagnostic threshold for closer monitoring. This is clinically meaningful but should not be framed as "identifying at-risk cases not captured by conventional BP screening," which implies a larger predictive gap than 3.5 mmHg represents. The clinical interpretation section should be precise about this.
**Recommendation**	Present the mean BP values of discordant cases (true positives missed by threshold, false positives flagged above threshold) as a formal results element, not just response letter content. Frame the added value as: the model identifies women whose BP is borderline — within a clinically relevant margin of the diagnostic threshold — and who may benefit from repeat measurement or closer observation. This is accurate and defensible; the current framing of "identifying at-risk cases missed by conventional screening" is an overstatement.

5. Minor Issues

ID	Location	Issue	Suggested Action
mn1	Abstract	Abstract now mentions Brier score in the response letter but it is unclear whether the calibration metrics (Brier score 0.08, calibration slope 0.94) appear in the abstract or only in the body. For a non-technical audience, a single sentence on calibration would reinforce the "proof-of-concept" framing.	Add one sentence to the abstract noting that calibration was assessed and the model is moderately well-calibrated (Brier score 0.08), consistent with a screening tool rather than a precision diagnostic.
mn2	Methods — feature set	Gestational age was removed from the final feature set by RFE. The authors note this prevented subgroup analysis by trimester. However, the feature importance plot (SHAP) should confirm whether gestational age appeared in preliminary feature selection rounds and at what importance rank before removal.	Add a footnote or sentence noting gestational age's importance rank in the preliminary feature set before RFE elimination, to confirm the decision was data-driven rather than arbitrary.
mn3	Results — Figure 3 (cross-validation)	Confidence intervals are stated as present but "not clearly visible due to small scale." This was flagged	Increase axis scale or add a companion table with mean and 95% CI for each

ID	Location	Issue	Suggested Action
		at R1 and the response deferred resolution to the Discussion.	model's cross-validation AUC. A small supplementary table resolves this without figure redesign.
mn4	Discussion — ethical framing	The authors added three references on ethical considerations in ML for health (Rajkomar, Wiens, WHO). These are appropriate but are not discussed substantively — they appear only in the reference list. One sentence in the Discussion should apply at least one of these frameworks to the specific deployment context (e.g., fairness implications of excluding 73% of records, most of whom are systematically different from the analytic cohort).	Add one sentence applying the ethical framework references to the documented exclusion-bias concern.
mn5	Data availability	The manuscript states source code is published on GitHub but does not provide the URL in the manuscript text.	Add the GitHub repository URL in the Data Availability statement or Methods section under "code reproducibility."

6. Statistical and Methodological Review

Element	Assessment	Notes
Model selection	Adequate	Five classifiers compared; XGBoost selected based on validation AUC and sensitivity; choice is defensible for tabular data in a class-imbalanced setting
Cross-validation	5-fold; adequate for training	Hyperparameter tuning via grid search (50 fits); parameters reported; adequate for reproducibility
Class balancing method	Inconsistent — see M2	SMOTE vs. random undersampling inconsistency must be resolved before publication
Validation design	Temporal split; adequate	Training 2023, validation April-May 2024; temporal independence confirmed; facility-network limitation noted (Mod2)
Calibration	Brier score 0.08; adequate	Calibration slope 0.94 and intercept -0.08 indicate moderate calibration; reliability diagram added; this is a meaningful R2 addition
Precision-recall curve	Added; adequate	PR curve (Figure 8) is the appropriate primary performance visual for an imbalanced dataset; its inclusion at R2 is a substantive methodological improvement
Missing data handling	Listwise deletion; partially justified	MNAR rationale is stated; systematic exclusion comparison analysis described but not formally presented — see C1
Feature selection	Multi-method; adequate	Pearson correlation + clinical input + chi-square + mutual information + RFE; multi-method approach is now adequately described
SHAP analysis	Present; adequate	SHAP values used for global feature importance; axis labels and clinical interpretation text added per R1 request; SHARP typo corrected

Element	Assessment	Notes
Outcome definition validity	Fundamental limitation; transparently disclosed	BP-defined proxy label, not clinically confirmed HDP; circular reasoning acknowledged; data leakage subsection present; model correctly repositioned

7. Presentation and Clarity

Element	Assessment	Recommendation
Title	Revised; adequate	"Machine Learning for Risk Stratification of Hypertensive Disorders of Pregnancy: Enhancing Clinical Efficiency in low-resource Antenatal Care in Tanzania" — accurately reflects the reframed scope; no further change required
Abstract	Substantially improved	Sensitivity-precision trade-off is now stated; dataset construction is clarified; proof-of-concept framing is appropriate. Minor gap: calibration metrics not mentioned (see mn1)
Introduction	Restructured; adequate	Logical flow (problem → opportunity → gap → objectives) implemented; comparison with prior ML-HDP models added; research gap explicitly stated
Methods	Most improved section	Preprocessing, feature selection, cross-validation, hyperparameter tuning, SMOTE/undersampling, calibration all now described; SMOTE vs. undersampling inconsistency must be resolved (M2)
Results	Improved but residual gap	Confusion matrix, ROC, PR curve, SHAP, calibration plot added; near-perfect training metric caveat still requires placement in manuscript text (M1)
Discussion	Improved; two gaps remain	Precision-sensitivity trade-off discussed; workload quantification added; MNAR deployment implication and discordant case BP values need explicit text (Mod1, Mod3)
Figure quality	Generally adequate	Figures 8 (PR curve) and 9 (calibration) are additions from R2; Figure 3 CI visibility unresolved (mn3); SHAP labels improved
Terminology	Now consistent	"HDP" used consistently throughout; "SHARP" corrected to "SHAP"; "SGB" corrected to "XGBoost"

8. Abstract and Title Review

Element	Status	Comment
Title	Acceptable	Revised title accurately reflects "risk stratification" scope; removes overstated "early detection" framing; accepted
Abstract — background	Acceptable	HDP as 34% of direct obstetric deaths in Tanzania; IDF global burden context removed from abstract; appropriate for article type
Abstract — methods	Improved	Dataset construction (337,027 visits → 187,438 clients → balanced training n=3,788 → validation n=120,232) is now described; adequate

Element	Status	Comment
Abstract — results	Acceptable with minor gap	Sensitivity 100%, precision 14%, AUC 0.95 reported; trade-off acknowledged; calibration metrics absent from abstract (see mn1)
Abstract — conclusion	Acceptable	Framed as proof-of-concept; scaling prerequisites noted; no longer deployment-ready claim; appropriate
Keywords	Acceptable	"machine learning, digital health, maternal predictions, hypertensive disorders during pregnancy" — adequate; "risk stratification" could replace "maternal predictions" for precision

9. References and Citations

Assessment Area	Finding
Reference base adequacy	Substantially expanded from R0; now includes Montgomery-Csobán et al. (Lancet Digital Health 2024), Li et al. (Pregnancy Hypertension 2021), Chen & Ma (Scientific Reports 2025), Alkhodari et al. (2023 narrative review), Ukah et al. (2018 systematic review), fullPIERS/PIERS (von Dadelszen), ethical ML references (Rajkomar, Wiens, WHO)
Comparison with prior HDP models	Adequate: AUC comparisons with Li et al. (0.81-0.86) and Chen & Ma (accuracy 84%, precision 79%) are now included; these appropriately contextualise the Tanzania model's precision deficit
Ethical references	Three references added (Rajkomar, Wiens, WHO) but not substantively discussed in text — see mn4
Data availability reference	DataverseNL DOI listed in submission form but GitHub URL for code not in manuscript text — see mn5
Reference to WHO ANC guidelines	Present; grounding of recommendation for 8+ ANC visits is consistent with cited guideline
Circular citation risk	No circular citations identified; response letter references (Montgomery-Csobán, Ukah) are appropriately integrated into the manuscript
