# Supplementary material for: Machine learning for risk stratification of hypertensive disorders of pregnancy: Enhancing clinical efficiency in low-resource antenatal care in Tanzania
Source: PLOS Digit Health. 2026 Jul 2;5(7):e0001468. doi: 10.1371/journal.pdig.0001468 (PMC13327181; doi:10.1371/journal.pdig.0001468)
Supplement: Attachment — Submitted filename: Manuscript Review.docx [file pdig.0001468.s001.docx]

**REVIEW OF HARNESSING DIGITAL HEALTH AND MACHINE LEARNING FOR EARLY DETECTION OF HYPERTENSIVE DISORDERS OF PREGNANCY: INSIGHTS FROM LOW-RESOURCE SETTINGS, TANZANIA**

**Abstract**

This abstract takes on a critical issue, Tanzania’s persistently high maternal mortality, and does so in a way that feels timely and innovative. Using digital antenatal care records and machine learning to predict hypertensive disorders of pregnancy is a strong framing, and the sheer scale of the dataset (300,000 visits) immediately makes the work stand out. Highlighting the comparison of multiple models before choosing XGBoost also signals methodological care, which is a plus for readers.

At the same time, the way the results are presented feels a little unbalanced. The abstract emphasizes the high accuracy and AUC, but the precision (14%) and F1-score (24%) tell a very different story. Those numbers suggest the model isn’t yet reliable enough for clinical use, and that point is downplayed. A quick acknowledgment that the model favors sensitivity at the cost of precision would help set more realistic expectations about its utility in a low-resource clinical environment.

There’s also some confusion in the dataset description that could use improvement. The authors say they analyzed 300,000 records, but then note only 3,788 were in the training dataset with a 50/50 distribution. That sounds like some balancing or sub-sampling was done, but it isn’t explained. Even a short clarification in the abstract would prevent readers from questioning whether the results are generalizable. Similarly, mentioning how missing values were handled, or whether repeated visits from the same women were included, would add a lot of credibility.

Lastly, the conclusion leans a bit too optimistic. It’s fair to say that AI can support earlier detection and potentially reduce maternal mortality, but the abstract should frame these results as proof-of-concept rather than ready-to-deploy. The mention of data quality is on point, but it could go further, scaling models like this will also require stronger infrastructure, training, and policy support. Balancing optimism with realism would make the abstract much stronger and more trustworthy.

**Introduction**

The introduction does a good job of framing the study within the broader context of machine learning applications in maternal health. The authors clearly highlight that ML has been used to predict conditions such as pre-eclampsia, gestational diabetes, and preterm labor, and they link these efforts to the growing availability of digital health data. The reference to WHO recommendations on early detection is particularly useful, since it grounds the study in internationally recognized priorities for improving maternal health outcomes. Overall, the opening establishes that the topic is both relevant and timely.

At the same time, the introduction feels too expansive and could be made more focused. The section on antenatal care reporting provides helpful background but it reads more like a health systems overview than part of a research introduction. It would be stronger if it were condensed and framed in terms of why gaps in ANC reporting matter for early detection of hypertensive disorders of pregnancy. The same is true for the long description of the Unified Community System. While the details are interesting, not all of them are necessary in the introduction. Some information could be moved to the Methods section, while the introduction could highlight more directly how the UCS enables new possibilities for predictive modeling in Tanzania.

The study objectives are well defined but they appear too late in the introduction. Presenting them earlier, right after describing the maternal health burden and the potential role of ML, would give readers a clearer sense of direction. As written, the objectives risk being overshadowed by long background sections. A sharper structure would move from problem (maternal mortality and inadequate ANC data), to opportunity (ML models applied to routine digital data), to the specific research goals. This would keep readers oriented and make the originality of the study stand out more clearly.

Finally, the introduction would be stronger if it more explicitly identified the research gap this study is addressing. The authors mention that ML has been applied to maternal health outcomes, but it is not clear what has been missing from past studies that this paper uniquely contributes. Is it the scale of the dataset, the Tanzanian context, or the integration with a national digital health system? Stating this gap directly would make the novelty of the work more visible. Similarly, acknowledging early on that routine data may have quality issues would strike a more balanced tone and prepare readers for the practical challenges that the study addresses.

**Methods**

The Methods section provides a good overview of the study design, dataset, and analytic approach. The use of a retrospective cohort design with routine antenatal care data is appropriate given the study objectives, and the description of the Unified Community System adds valuable context on how the data was collected. The attention to data cleaning and preprocessing, including the conversion of variable formats, duplicate removal, and thoughtful aggregation of longitudinal records into cross-sectional format, shows care in preparing the dataset for analysis. Involving obstetricians and public health experts in feature selection is also a strength, as it helps ensure clinical relevance.

At the same time, several aspects of the methods would benefit from additional clarity and detail. For example, the decision to collapse repeated ANC visits into a single record per client may simplify the modeling process, but it also risks losing important temporal information that could influence hypertensive risk. A brief justification for this choice, or at least an acknowledgment of the trade-off, would make the approach more transparent. Similarly, while the authors note that Pearson correlation was used to explore the relationship between predictors and the outcome, correlation may not be the best measure for categorical or non-linear relationships. Providing more detail on why this method was chosen, and whether other feature selection techniques were considered, would strengthen confidence in the modeling pipeline.

The description of the outcome definition is a critical part of this study, and the authors are right to highlight the challenges with underreporting of hypertensive disorders in the dataset. However, the reliance on a single threshold-based calculation to define cases may oversimplify clinical reality. Blood pressure measurements in routine ANC settings can be variable due to equipment quality, staff training, or measurement error. A discussion of how these potential inaccuracies might affect case definition, or whether multiple readings per patient were considered, would make the outcome measure more robust.

Finally, the modeling approach is described in broad strokes but lacks important details for reproducibility. For instance, it is not clear whether cross-validation was used during model training, what hyperparameter tuning was performed, or whether class imbalance was addressed explicitly. Reporting accuracy alone is not sufficient, since accuracy can be inflated in imbalanced datasets; the inclusion of precision, recall, F1-score, and calibration metrics would make the evaluation more complete. The section on model application is a useful addition, showing how the model was tested on new UCS data, but it could go further by explaining how the model’s predictions compared to traditional case definitions and what implications this has for real-world adoption.

**Results**

The Results section is thorough and presents a wide range of analyses, from descriptive statistics through model validation and feature importance. The descriptive findings are helpful, especially the breakdown of ANC visits before and after collapsing records into individual clients. This makes it clear how much information is lost when visits are aggregated and highlights an important contextual point for interpreting the models. The histogram of gestational age and the correlation heatmap add useful visualization, showing both the distribution of visits and the strength of associations with hypertensive disorders of pregnancy.

At the same time, there are several areas that would benefit from refinement. The correlation results are described as significant, but Pearson correlation is a limited tool for understanding complex relationships between predictors and outcomes, especially when variables are non-linear or categorical. More robust feature selection methods could be mentioned, or at least an acknowledgment that correlation is a preliminary measure. In addition, the decision to balance the dataset by downsampling or resampling to achieve equal risk and no-risk groups should be described in more detail, as this step has a major influence on model performance and interpretability.

The model training and validation results appear strong at first glance, with very high cross-validation scores across all classifiers. However, the near-perfect accuracy shown in Table 2 (100 percent across precision, recall, and F1-score in the small test dataset) raises concerns about overfitting. This becomes evident when the model is applied to new UCS data, where performance drops considerably, especially in precision (14 percent) and F1-score (24 percent). These results deserve a more critical discussion in the Results section itself, not just later in the paper. Presenting calibration curves, confusion matrices, or a breakdown of false positives versus false negatives would make the performance evaluation more transparent.

The feature importance plot is a helpful addition, as it aligns with clinical expectations that systolic and diastolic blood pressure are the strongest predictors. However, the figure could be described in a way that ties more directly to clinical practice. For example, explaining that BMI and proteinuria contribute modestly, while syphilis status and temperature contribute little, would help readers see which features add incremental value beyond standard hypertension screening.

Overall, the Results section demonstrates impressive effort in data analysis and visualization, but it could be improved by providing more nuance around the limitations of the modeling approach. Clearer acknowledgment of the gap between training performance and real-world validation would make the results more credible and prepare the ground for a balanced discussion.

**Discussion**

The discussion section does a good job of connecting the study results to the bigger picture of maternal health in low-resource settings. The authors clearly explain the challenges of poor data quality, lack of digital tools, and limited trained staff, while also pointing out that Tanzania’s investment in the UCS system is creating opportunities for digital health. It is also a strength that the authors show how the ML model identified predictors like BMI and protein in urine, which are often overlooked in routine care. This makes the case that ML can add value beyond just blood pressure readings.

However, the discussion tends to emphasize the positive findings more than the limitations. The model reached high accuracy but had very low precision, meaning it predicted a large number of women as “at risk” when they were not. This is an important issue because in a health system with limited resources, too many false positives could overwhelm staff and equipment. The authors should discuss this trade-off more directly and explain whether the model in its current form would really help health workers, or if it first needs further refinement.

The limitations section is well written but could be clearer. The authors explain that HDP was not directly recorded in the UCS and that blood pressure values were used instead. This is an important point, but it should also be noted that blood pressure in routine ANC can sometimes be recorded inaccurately, which could affect results. The lack of follow-up data is another key issue, because without clinical confirmation, it is difficult to know how reliable the predictions are outside the dataset.

The recommendations and key messages are helpful but read a bit broad. They could be made stronger by giving more concrete steps, such as improving digital training for health workers, making ANC data entry more standardized, or piloting the ML model in a few facilities before thinking about scaling up. These kinds of practical next steps would make the recommendations more useful to policymakers and health system leaders.

Overall, the discussion is clear and well-structured, but it needs to be more balanced. The authors should talk more openly about the limits of the model, especially the low precision, and give sharper, more realistic recommendations. This would make the paper stronger and show both the promise and the challenges of applying ML to maternal health in Tanzania.

**Conclusion**

This paper takes on an important problem and shows how machine learning could be used to predict high blood pressure in pregnancy using routine health data in Tanzania. The study makes good use of a large dataset, tests several models, and connects the work to real-world health challenges, which makes it both timely and relevant.

At the same time, the paper needs more clarity and balance. The results show very high accuracy but very low precision, which limits how useful the model would be in practice. The methods also need to be explained in more detail so readers can better understand and trust the findings. If the authors address these issues and provide more realistic recommendations for how the model could be used in low-resource settings, this study could make a strong contribution to maternal health research.

Thank you.
