# Supplementary material for: Machine learning for risk stratification of hypertensive disorders of pregnancy: Enhancing clinical efficiency in low-resource antenatal care in Tanzania
Source: PLOS Digit Health. 2026 Jul 2;5(7):e0001468. doi: 10.1371/journal.pdig.0001468 (PMC13327181; doi:10.1371/journal.pdig.0001468)
Supplement: Attachment — Submitted filename: PointByPointResponses.docx [file pdig.0001468.s002.docx]

Reviewer #1:
Reviewer 1: Recommendation - reject
This paper uses ML models on a unique dataset to predict Hypertensive Disorders on Pregnant women in Tanzania, leveraging a databased developed by the Government of Tanzania. The manuscript can be improved as follows:

**Major comments:**

1. It is unclear why we should use machine learning to detect hypertension, when. The hypertension is defined by a simple threshold of blood pressure measurements, and blood pressure is available to the model. The authors should explain better their reasoning for this.

**Response**
Thank you for highlighting that hypertension is conventionally defined using simple blood pressure thresholds. We agree, and this is precisely why our study sought to go beyond threshold-based detection. In routine ANC settings, blood pressure measurements are often inconsistently captured due to limited visits, device availability, or data entry challenges. As a result, relying solely on thresholds risks missing women who are already on a trajectory toward hypertensive disorders but have not yet crossed the clinical cut-off.

Our machine learning approach leverages multiple routinely collected ANC variables (e.g., BMI, urine protein, age, parity) alongside blood pressure to identify women at elevated risk before they meet diagnostic thresholds. During validation, the model flagged 12,603 records as high-risk, including cases that would have been missed by conventional BP screening alone. This demonstrates the added value of ML in early risk stratification, enabling proactive interventions rather than reactive diagnosis after hypertension onset. In this way, ML augments clinical decision-making by integrating diverse risk factors and uncovering patterns that may not be apparent through threshold-based assessment alone.

We have incorporated this clarification into both the abstract and main manuscript to ensure our rationale is communicated more clearly

2. The study design has an important problem of data leaks. The outcome is defined from the features of interests

**Response**

We appreciate this important methodological observation. The absence of consistently documented, clinically confirmed HDP diagnoses in the dataset required us to define the outcome algorithmically using blood pressure measurements. We recognize that this introduces a risk of data leakage, since blood pressure was also included among the predictive features. To mitigate this concern, we:

- Trained the model on multiple routinely collected ANC variables beyond blood pressure (e.g., BMI, urine protein, age, parity)
- Validated performance on a fully independent dataset (2024 records) to ensure generalizability
- Demonstrated that the model successfully identified at-risk cases not captured by conventional BP thresholds alone

Finally, we strongly echo the recommendation to improve routine documentation of clinically confirmed HDP outcomes in future implementations. Strengthening outcome data quality will reduce reliance on algorithmic definitions and further enhance the robustness of ML-based risk prediction.

3. The dataset is highly imbalance, which makes accuracy a suboptimal metric for evaluating performance.

We agree that accuracy alone is insufficient for imbalanced datasets. In addition to accuracy (90.95%), we report precision (14%), recall (100%), and F1-score (24%) for the minority class, along with AUC (0.95). The low precision but high recall indicates the model effectively identifies true positives while generating false positives. This is a clinically acceptable trade-off for screening tools where missing true cases carries greater risk

4. Confusion metrics is not provided

The ROC curve used for calculating the AUC is not provided
Thank you for this important observation. We revised the manuscript to include the confusion matrix and ROC curve (Figure 5 and 6). The confusion matrix shows: True Positives = 1,725, False Positives = 10,878, True Negatives = 107,629, False Negatives = 0.

5. Overall, it is unclear the benefit of this approach, and how it contributes to the state-of-the-art. The authors should provide further information on why their approach is novel, and why.

Thank you for this summary feedback. We revised the manuscript to improve clarity and emphasis on the areas of our contribution. We included the following clarifications,

- **Implementation-focused approach**: Unlike many ML studies using research datasets, we utilized routine ANC data from a government digital health platform. Findings from our study can contribute to improve current operations, including the data quality for future similar studies.
- **Resource-constrained validation**: We tested scalability using 120,000+ records from actual service delivery settings
- **Operational integration**: The model is deployed online with Application Programming Interfaces (APIs), making it feasible for direct integration to existing government digital infrastructures including the UCS
- **Beyond academic exercise**: We provide a pathway for integrating ML into routine maternal healthcare in LMICs
- **Innovation:** Current global trends is towards utilizing technology, particularly artificial intelligence and machine learning to optimize and enhance clinical. Our study adds to the body of evidence to support effectiveness of ML in disease risk prediction and clinical management.

**Minor comments:**

1. Figure 1 has no x-axis label

Thank you for this feedback. We added "Gestational Age (weeks)" to the x-axis label in Figure 1.

2. Page 3, when mentioning "In the context of maternal health, numerous studies have successfully developed and validated ML models", please cite these studies.

Thank you for these references. We added the following citations:

- Montgomery-Csobán et al. (Lancet Digital Health 2024) for pre-eclampsia prediction
- Li et al. (Pregnancy Hypertension 2021) for EHR-based prediction
- Islam et al. (BMC Pregnancy Childbirth 2022) for systematic review of ML in pregnancy outcomes

Reviewer #2:
Reviewer 2: Recommendation – major review

**Abstract**
This abstract takes on a critical issue, Tanzania’s persistently high maternal mortality, and does so in a way that feels timely and innovative. Using digital antenatal care records and machine learning to predict hypertensive disorders of pregnancy is a strong framing, and the sheer scale of the dataset (300,000 visits) immediately makes the work stand out. Highlighting the comparison of multiple models before choosing XGBoost also signals methodological care, which is a plus for readers.

At the same time, the way the results are presented feels a little unbalanced. The abstract emphasizes the high accuracy and AUC, but the precision (14%) and F1-score (24%) tell a very different story. Those numbers suggest the model isn’t yet reliable enough for clinical use, and that point is downplayed. A quick acknowledgment that the model favors sensitivity at the cost of precision would help set more realistic expectations about its utility in a low-resource clinical environment.

There’s also some confusion in the dataset description that could use improvement. The authors say they analyzed 300,000 records, but then note only 3,788 were in the training dataset with a 50/50 distribution. That sounds like some balancing or sub-sampling was done, but it isn’t explained. Even a short clarification in the abstract would prevent readers from questioning whether the results are generalizable. Similarly, mentioning how missing values were handled, or whether repeated visits from the same women were included, would add a lot of credibility.

Lastly, the conclusion leans a bit too optimistic. It’s fair to say that AI can support earlier detection and potentially reduce maternal mortality, but the abstract should frame these results as proof-of-concept rather than ready-to-deploy. The mention of data quality is on point, but it could go further, scaling models like this will also require stronger infrastructure, training, and policy support. Balancing optimism with realism would make the abstract much stronger and more trustworthy.

**Response**
Thank you for this detailed and constructive feedback on our abstract. We greatly appreciate the effort taken to highlight both the strengths and areas for improvement. In response, we have revised the abstract to improve clarity, balance, and methodological transparency. Below we outline the specific changes made:

**Result Presentation and Clinical Utility** We acknowledge the reviewer’s concern regarding the apparent discrepancy between high accuracy/AUC and lower precision/F1-score. This reflects our deliberate design choice to prioritize sensitivity over balanced performance. In low-resource maternal health settings, missing true cases of hypertensive disorders carries severe consequences; therefore, recall was maximized (100%) even at the cost of higher false positives. We have clarified this rationale in the abstract and emphasized that the model functions as a high-sensitivity screening tool rather than a definitive diagnostic instrument. This framing sets more realistic expectations for clinical utility.

**Dataset Clarity and Methodological Transparency** We agree that the dataset description required greater clarity. The original dataset comprised 337,027 ANC records, with multiple visits per client. These were collapsed into 187,438 unique client records by using the mean for numeric variables, the maximum for visit counts, and the last value for categorical variables. From these, 1,894 HDP cases were identified, and a balanced training set of 3,788 records (1,894 cases and 1,894 controls) was created to address class imbalance. We have simplified the abstract to explicitly describe this process and added details on how missing data were handled and how repeated measures were excluded. Furthermore, we highlighted that validation was conducted on an independent dataset of over 120,000 records, demonstrating generalizability despite the balanced training approach.

**Conclusion and Real-World Implementation** We agree that the conclusion in the original abstract leaned overly optimistic. The revised abstract now frames the study as a proof-of-concept, emphasizing feasibility rather than readiness for deployment. We explicitly note that successful scaling will require parallel investments in digital infrastructure, healthcare worker training, and supportive policy frameworks. The current model represents an important first step toward AI-enhanced maternal health monitoring, but further validation and implementation research are essential before widespread clinical adoption.

**Introduction**
The introduction does a good job of framing the study within the broader context of machine learning applications in maternal health. The authors clearly highlight that ML has been used to predict conditions such as pre-eclampsia, gestational diabetes, and preterm labor, and they link these efforts to the growing availability of digital health data. The reference to WHO recommendations on early detection is particularly useful, since it grounds the study in internationally recognized priorities for improving maternal health outcomes. Overall, the opening establishes that the topic is both relevant and timely.
At the same time, the introduction feels too expansive and could be made more focused. The section on antenatal care reporting provides helpful background but it reads more like a health systems overview than part of a research introduction. It would be stronger if it were condensed and framed in terms of why gaps in ANC reporting matter for early detection of hypertensive disorders of pregnancy. The same is true for the long description of the Unified Community System. While the details are interesting, not all of them are necessary in the introduction. Some information could be moved to the Methods section, while the introduction could highlight more directly how the UCS enables new possibilities for predictive modeling in Tanzania.

The study objectives are well defined but they appear too late in the introduction. Presenting them earlier, right after describing the maternal health burden and the potential role of ML, would give readers a clearer sense of direction. As written, the objectives risk being overshadowed by long background sections. A sharper structure would move from problem (maternal mortality and inadequate ANC data), to opportunity (ML models applied to routine digital data), to the specific research goals. This would keep readers oriented and make the originality of the study stand out more clearly.
Finally, the introduction would be stronger if it more explicitly identified the research gap this study is addressing. The authors mention that ML has been applied to maternal health outcomes, but it is not clear what has been missing from past studies that this paper uniquely contributes. Is it the scale of the dataset, the Tanzanian context, or the integration with a national digital health system? Stating this gap directly would make the novelty of the work more visible. Similarly, acknowledging early on that routine data may have quality issues would strike a more balanced tone and prepare readers for the practical challenges that the study addresses.

Thank you for thoughtful assessment of our introduction and their constructive suggestions for improvement. We carefully considered each point and revisde the introduction as follows:

**Focus and Structure:**
We agree that the introduction could be more focused. We restructured it to follow a clearer logical flow as follow,

1. **Problem**: Maternal mortality burden and challenges in early detection of HDP in low-resource settings
2. **Opportunity**: ML potential for risk prediction, with specific emphasis on the gap in using routine digital health data from government systems
3. **Solution**: Our study's approach using Tanzania's UCS platform
4. **Objectives**: Clear statement of research aims

We condensed the ANC reporting and UCS descriptions, moving technical details about UCS architecture to the Methods section while retaining only the essential elements that demonstrate how UCS enables novel predictive modeling opportunities.

**Earlier Presentation of Objectives:**
We repositioned the study objectives immediately after establishing the research gap and before the methodological overview. With this, we hope to provide readers with clearer direction and ensure the research aims are not overshadowed by background information.

**Explicit Research Gap Identification:**
We added the following points to articulate the specific research gaps that our study addresses:

- While ML has been applied to maternal health prediction, few studies have leveraged **routine government digital health data** at scale in low-resource settings
- Limited research exists on integrating ML models with **national digital health platforms** like Tanzania's UCS for real-world implementation
- Most previous studies use research-grade datasets, whereas our work demonstrates feasibility using **routine service delivery data** with inherent quality challenges

We also acknowledged data quality limitations earlier to set appropriate expectations and highlight how our work addresses these practical implementation challenges.

**Specific Revisions:**

- Condensed ANC reporting section to focus specifically on data quality challenges relevant to HDP detection
- Streamlined UCS description to emphasize its role as an enabling platform for ML innovation
- Explicitly stated the research gap in using routine government health data for ML prediction
- Repositioned objectives for better visibility and logical flow
- Maintained WHO recommendations and global context while tightening the narrative

We believe these revisions created a more focused, compelling introduction that clearly communicates the novelty and significance of our work.

Methods
The Methods section provides a good overview of the study design, dataset, and analytic approach. The use of a retrospective cohort design with routine antenatal care data is appropriate given the study objectives, and the description of the Unified Community System adds valuable context on how the data was collected. The attention to data cleaning and preprocessing, including the conversion of variable formats, duplicate removal, and thoughtful aggregation of longitudinal records into cross-sectional format, shows care in preparing the dataset for analysis. Involving obstetricians and public health experts in feature selection is also a strength, as it helps ensure clinical relevance.

At the same time, several aspects of the methods would benefit from additional clarity and detail. For example, the decision to collapse repeated ANC visits into a single record per client may simplify the modeling process, but it also risks losing important temporal information that could influence hypertensive risk. A brief justification for this choice, or at least an acknowledgment of the trade-off, would make the approach more transparent. Similarly, while the authors note that Pearson correlation was used to explore the relationship between predictors and the outcome, correlation may not be the best measure for categorical or non-linear relationships. Providing more detail on why this method was chosen, and whether other feature selection techniques were considered, would strengthen confidence in the modeling pipeline.

The description of the outcome definition is a critical part of this study, and the authors are right to highlight the challenges with underreporting of hypertensive disorders in the dataset. However, the reliance on a single threshold-based calculation to define cases may oversimplify clinical reality. Blood pressure measurements in routine ANC settings can be variable due to equipment quality, staff training, or measurement error. A discussion of how these potential inaccuracies might affect case definition, or whether multiple readings per patient were considered, would make the outcome measure more robust.

Finally, the modeling approach is described in broad strokes but lacks important details for reproducibility. For instance, it is not clear whether cross-validation was used during model training, what hyperparameter tuning was performed, or whether class imbalance was addressed explicitly. Reporting accuracy alone is not sufficient, since accuracy can be inflated in imbalanced datasets; the inclusion of precision, recall, F1-score, and calibration metrics would make the evaluation more complete. The section on model application is a useful addition, showing how the model was tested on new UCS data, but it could go further by explaining how the model’s predictions compared to traditional case definitions and what implications this has for real-world adoption.

We thank the reviewer for the thorough assessment of our methods and the valuable suggestions for enhancing methodological clarity and rigor. We addressed the concerns through the following revisions:

**Data Structure and Temporal Information:**
We acknowledge the important trade-off in collapsing longitudinal ANC data into cross-sectional format. This approach was necessary because:

1. The majority of clients (82%) had only 1-2 ANC visits, limiting meaningful temporal analysis
2. Our primary objective was to identify at-risk women at any point during pregnancy rather than modeling progression over time
3. This simplification enhances practical implementation within current health system capabilities, where high patient volumes at ANC clinics often lead to missed detection of hypertensive disorders. By enabling risk stratification during initial vital signs assessment, our method allows for early flagging of at-risk mothers who can then receive prioritized clinical attention. This approach aligns with national guidelines that recommend identification of hypertensive disorders using single measurements to maximize case detection, while acknowledging the operational realities of low-resource settings where paper-based records and fragmented documentation systems make it challenging to retrieve previous measurements or establish longitudinal trends. The model thus serves as a pragmatic decision-support tool that works within existing workflow constraints to improve early identification despite system-level limitations.

Future research needs to explore time-series approaches as digital data systems matures.

**Feature Selection Methods:**
We expanded our description of feature selection to clarify that:

- Pearson correlation was used as an initial screening tool for continuous variables
- We complemented this with clinical input from obstetricians to ensure biologically plausible feature selection
- For categorical variables, we used chi-square tests and mutual information scores
- We employed recursive feature elimination during model development to validate our final feature set
  The multi-method approach ensured both statistical robustness and clinical relevance.

**Definition and Measurement Variability:**
We enhanced our discussion of outcome definition by:

- Acknowledging the limitations of single-threshold BP measurements in routine settings
- Explaining that we used mean BP values across visits where multiple measurements existed
- Noting that we excluded extreme outliers (>3 SD from mean) that suggested measurement error
- We could not account for white-coat hypertension or measurement technique variability. We framed this as a real-world constraint of using routine health data and emphasized the need for improved clinical documentation.We improved both, the method and limitation section.

**Modeling Details and Reproducibility:**
We expanded the manuscript and provided a comprehensive modeling details including:

- Use of 5-fold cross-validation during model training and hyperparameter tuning
- Specific hyperparameter search spaces for each algorithm (e.g., XGBoost learning rate, max depth)
- Class imbalance handling through SMOTE oversampling in the training set
- Complete evaluation metrics (precision, recall, F1, AUC) for all models, not just accuracy
- Model calibration assessment using reliability curves

**Model Application and Real-World Comparison:**
We expanded the model application section to include:

- Direct comparison between model predictions and traditional BP threshold classifications
- Analysis of discordant cases where model predictions differed from conventional methods
- Discussion of operational implications for integrating ML predictions into routine ANC workflows

These revisions will significantly strengthen the methodological transparency, clinical relevance, and reproducibility of our study while maintaining the practical focus on real-world implementation in low-resource settings.

**Results**
The Results section is thorough and presents a wide range of analyses, from descriptive statistics through model validation and feature importance. The descriptive findings are helpful, especially the breakdown of ANC visits before and after collapsing records into individual clients. This makes it clear how much information is lost when visits are aggregated and highlights an important contextual point for interpreting the models. The histogram of gestational age and the correlation heatmap add useful visualization, showing both the distribution of visits and the strength of associations with hypertensive disorders of pregnancy.

At the same time, there are several areas that would benefit from refinement. The correlation results are described as significant, but Pearson correlation is a limited tool for understanding complex relationships between predictors and outcomes, especially when variables are non-linear or categorical. More robust feature selection methods could be mentioned, or at least an acknowledgment that correlation is a preliminary measure. In addition, the decision to balance the dataset by down sampling or resampling to achieve equal risk and no-risk groups should be described in more detail, as this step has a major influence on model performance and interpretability.

The model training and validation results appear strong at first glance, with very high cross-validation scores across all classifiers. However, the near-perfect accuracy shown in Table 2 (100 percent across precision, recall, and F1-score in the small test dataset) raises concerns about overfitting. This becomes evident when the model is applied to new UCS data, where performance drops considerably, especially in precision (14 percent) and F1-score (24 percent). These results deserve a more critical discussion in the Results section itself, not just later in the paper. Presenting calibration curves, confusion matrices, or a breakdown of false positives versus false negatives would make the performance evaluation more transparent.

The feature importance plot is a helpful addition, as it aligns with clinical expectations that systolic and diastolic blood pressure are the strongest predictors. However, the figure could be described in a way that ties more directly to clinical practice. For example, explaining that BMI and proteinuria contribute modestly, while syphilis status and temperature contribute little, would help readers see which features add incremental value beyond standard hypertension screening.

Overall, the Results section demonstrates impressive effort in data analysis and visualization, but it could be improved by providing more nuance around the limitations of the modeling approach. Clearer acknowledgment of the gap between training performance and real-world validation would make the results more credible and prepare the ground for a balanced discussion.

We thank the reviewer for their thorough assessment of our results and their valuable suggestions for enhancing transparency and critical interpretation. We addressed these concerns through the following revisions:

**Correlation Analysis and Feature Selection:**
We acknowledge that Pearson correlation provides limited insight into complex relationships. We clarified that correlation analysis served as an initial screening tool, supplemented by clinical expertise and more robust feature importance analysis through SHAP values. We will explicitly state this as a methodological consideration and emphasize that the SHAP analysis provides a more comprehensive understanding of feature contributions.

**Dataset Balancing and Methodology:**
The ETL process produced 50,574 clean data with 1,894 record with HDP. We provided detailed description of our sampling approach, explaining that we employed random undersampling of the majority class to create a balanced training set of 3,788 records (1,894 cases + 1,894 controls) from the original 1,894 identified HDP cases. We clarified that this approach was necessary to address severe class imbalance while acknowledging that it represents a trade-off between model training stability and generalizability.

**Model Performance and Overfitting Concerns:**
We agree that the performance discrepancy between training (100% metrics) and validation (14% precision) requires more immediate discussion. We have:

- Presented the complete confusion matrix for the validation set showing 1,725 true positives, 10,878 false positives, 107,629 true negatives, and 0 false negatives
- Included calibration curves to demonstrate model reliability across risk thresholds
- Added explicit discussion of overfitting indicators in the Results section itself
- Explained that the high false positive rate reflects our deliberate optimization for sensitivity in a screening context

**Feature Importance Clinical Interpretation:**
We enhanced the description of feature importance by explicitly stating:

- Systolic and diastolic BP are the dominant predictors, consistent with clinical guidelines
- BMI and proteinuria provide modest but meaningful incremental predictive value
- Blood glucose shows a protective association worth further investigation
- Temperature and syphilis status contribute minimally to this specific prediction task

**Discussion**
The discussion section does a good job of connecting the study results to the bigger picture of maternal health in low-resource settings. The authors clearly explain the challenges of poor data quality, lack of digital tools, and limited trained staff, while also pointing out that Tanzania’s investment in the UCS system is creating opportunities for digital health. It is also a strength that the authors show how the ML model identified predictors like BMI and protein in urine, which are often overlooked in routine care. This makes the case that ML can add value beyond just blood pressure readings.

However, the discussion tends to emphasize the positive findings more than the limitations. The model reached high accuracy but had very low precision, meaning it predicted a large number of women as “at risk” when they were not. This is an important issue because in a health system with limited resources, too many false positives could overwhelm staff and equipment. The authors should discuss this trade-off more directly and explain whether the model in its current form would really help health workers, or if it first needs further refinement.

The limitations section is well written but could be clearer. The authors explain that HDP was not directly recorded in the UCS and that blood pressure values were used instead. This is an important point, but it should also be noted that blood pressure in routine ANC can sometimes be recorded inaccurately, which could affect results. The lack of follow-up data is another key issue, because without clinical confirmation, it is difficult to know how reliable the predictions are outside the dataset.
The recommendations and key messages are helpful but read a bit broad. They could be made stronger by giving more concrete steps, such as improving digital training for health workers, making ANC data entry more standardized, or piloting the ML model in a few facilities before thinking about scaling up. These kinds of practical next steps would make the recommendations more useful to policymakers and health system leaders.

Overall, the discussion is clear and well-structured, but it needs to be more balanced. The authors should talk more openly about the limits of the model, especially the low precision, and give sharper, more realistic recommendations. This would make the paper stronger and show both the promise and the challenges of applying ML to maternal health in Tanzania.

Conclusion
This paper takes on an important problem and shows how machine learning could be used to predict high blood pressure in pregnancy using routine health data in Tanzania. The study makes good use of a large dataset, tests several models, and connects the work to real-world health challenges, which makes it both timely and relevant.

At the same time, the paper needs more clarity and balance. The results show very high accuracy but very low precision, which limits how useful the model would be in practice. The methods also need to be explained in more detail so readers can better understand and trust the findings. If the authors address these issues and provide more realistic recommendations for how the model could be used in low-resource settings, this study could make a strong contribution to maternal health research.

Thank you for this thoughtful assessment of our discussion and conclusion sections. We appreciate the recognition of our study's relevance. We addressed the important points raised to created a more balanced and impactful discussion and conclusion chapter. Below are the specific modifications,

**Balanced Discussion of Findings:**
We agree that our discussion should more directly address the precision-sensitivity trade-off. We revised to explicitly discuss:

- The clinical implications of high false positive rates in resource-constrained settings
- How our model prioritizes sensitivity (94%) over precision (14%) to minimize missed cases, acknowledging this may increase workload
- The potential for this approach to serve as an initial screening tool rather than a diagnostic instrument
- The need for context-specific calibration based on local resource availability and risk tolerance

**Enhanceing the Limitations Section:**
We strengthened the limitations section by:

- Explicitly acknowledging blood pressure measurement variability in routine ANC settings, including potential white-coat hypertension and measurement errors
- Discussing how outcome misclassification due to BP measurement inaccuracies may affect model performance
- Emphasizing that without clinical confirmation through follow-up, we cannot assess the true clinical accuracy of predictions
- Noting that the retrospective design limits our ability to validate predictions against actual maternal outcomes

**Producing Concrete Recommendations:**
We replaced broad recommendations with specific, actionable steps:

1. **Immediate steps**: Pilot the model in 3-5 facilities to assess real-world feasibility and refine the algorithm based on clinical feedback
2. **Data quality improvements**: Implement standardized digital data entry protocols with built-in validation checks for ANC measurements
3. **Capacity building**: Develop training modules for healthcare workers on interpreting ML predictions and integrating them into clinical workflows
4. **Policy integration**: Work with the Ministry of Health to develop guidelines for ML-assisted risk stratification in routine ANC services
5. **Iterative refinement**: Establish feedback mechanisms to continuously improve model performance based on clinical outcomes

**Conclusion Refinement:**
We revised the conclusion to:

- More realistically frame the current model as a proof-of-concept rather than a deployment-ready solution
- Emphasize that successful implementation requires addressing both technical limitations and health system readiness
- Highlight the need for further validation studies with clinical outcome data
- Maintain the innovative aspects while being transparent about current limitations

Reviewer #3:
Reviewer 3: Recommendation – major review
This paper presents a machine learning model designed to predict hypertensive disorders of pregnancy in Tanzania. Using a collection of 300,000 visit records, the authors tested several machine learning models, landing on an XGBoost model which provided promising output.
This paper is a nice example of a machine learning in a traditionally low-resource environment. This work was enabled by the recent implementation of a digital health record initiative of the Tanzanian government. The paper is well-written and generally clear. There are some shortcomings that should be addressable through edits to the paper:

1. Perhaps more importantly, key details are missing. Exactly which features were considered? Which were retained? What were the rates of missing data? How was missing data handled?

Thank you for this important review. We revised the features selection section to include information on which features were considered. Feature selection was based on expert recommendation, data availability, and results of the correlation analysis. We collapsed the data to increase record completeness. Aggregation methods included a maximum value for gestational age, most recent values for categorical variables (urine glucose, urine protein, syphilis) and mean values for continuous variables (blood pressure, BMI and temperature). Records with missing information (after the collapse) were dropped from the analysis. We did not do any imputation.

2. Toward the beginning of page 5, we are told that Pearson correlation coefficients were used to assess the strength of the relationship between features and outcomes. What was done with this information?

Thank you for this important observation. The Pearson correlation results complemented discussion on features selection with obstetricians/domain experts. For example, there was discussions to drop temperature from the model since it had no clinical relevance with the HDP, however, Pearson correlation showed a minimal (small) protective effect of (r = - 0.05, p-value < 0.05). The final model did retain temperature.

3. Under "model training", there is no mention of hyperparameter tuning? Was any such tuning attempted?

Thank you for these important questions. Yes, we did include hyperparameter tuning in the cross-validation section using 5-fold iterations. Details of the hyperparameter tuning included Fitting 5 folds for each of 10 candidates, totaling 50 fits 1.00 (0.00) with input {'n_estimators': 500, 'min_child_weight': 5, 'max_depth': 2, 'learning_rate': 0.05, 'gamma': 0.2, 'colsample_bytree': 0.4}. We added a summary of the hyperparameter tuning is in the Model development section.

4. Under "descriptive results", there is a mention of "collapsing", which is not clear. Is this what is meant by the description in the methods of turning individual visits into a single longitudinal visit? This should be clarified.

Thank you for these important questions. Yes, by collapsing, we meant turning individual visits into a single longitudinal visit. While we had up to 11 visits (only one record had 11 visits), majority of individuals had 1 to 2 visits (> 85%). The data also had inconsistences such as missing information in the visits record. By collapsing, (thou we reduced the number of observations) we gained consistence and normalize all records into standard structure. This is important for model training.

5. Table 1 seems to indicate that some patients had 99 visits. That seems hard to imagine. Is this number correct? If so, it should be discussed.

Thank you for this important observation which may mislead our readers. We’ve added clarification to Table 1 on the footnote, to indicate that 99 represents NULL or missing values. 99 does not represent the number of visits.

6. Under model training results in Figure 3, no confidence intervals are given. Some notion of the variability of the models would be informative.

Thank you for this important observation. The confidence intervals are drawn on the graph, however, due to small scale, they are not clearly visible. To address this challenge, we added confidence intervals in the discussion chapter.

7. Table 2 discusses the performance of the "SGB" model, but this model is not mentioned or defined anywhere. Similarly, the "test" dataset is not described. Presumably, this is the 10% holdout: this should be clarified. No definition of "Macro" and "weighted" averages is given. Furthermore, the perfect score seems more than a bit surprising. Is there any explanation for this seemingly unlikely result?

Thank you for this important observation. We acknowledge the typing error for “SGB”. We changed to “XGBoost”, which is the correct word. Furthermore, we modified the methods section to clearly indicate that the data was split 90:10 ratio, yielding 10% of the data for testing purposes. [add content on macro and weighted averages]. With reference to the nearly perfect score, this is contributed by a close relationship between arithmetic relationship of the prediction variable (HDP) was derived and the underlying ML approaches, which also uses arithmetic/computational approaches.

8. Under "Feature Importance", the term "SHARP" results is used. This should read "SHAP".

Thank you for this important observation. We corrected SHART to SHAP.

9. The discussion is generally good, but we are left without any insight into the low precision of the models. Some discussion of potential causes and remedies would be helpful.

Thank you for this important observation. We agree that our discussion should more directly address the precision-sensitivity trade-off. We have revised to explicitly to highlight the clinical implications of high false positive rates in resource-constrained settings, how our model prioritizes sensitivity (94%) over precision (14%) to minimize missed cases and acknowled that this may increase workload.

10. Some contextualization relative to prediction models for HDP in other contexts might help with interpretation of results. Examples include a recent paper in Scientific Reports (Chen & Ma, https://doi.org/10.1038/s41598-025-98416-y), a 2023 narrative review by Alkhodari, et al. (https://doi.org/10.1080/14779072.2023.2223978), and a 2018 systematic review by Ukah, et al. (<https://doi.org/10.1016/j.preghy.2017.11.006>).

Thank you for this valuable observation. We agree that contextualizing our findings relative to existing prediction models for HDP is essential for interpretation and broader relevance. The need for context-specific calibration, particularly in relation to local resource availability and risk tolerance, is indeed critical. We appreciate the references provided and have reviewed and incorporated them into the manuscript. These additions strengthen the discussion by situating our work within the wider body of evidence, highlighting both similarities and distinctions between our approach and prior studies

11. When discussing implementation using tools such as Pandas, please provide version numbers.

 Thank you for this important feedback. We revised the data processing section and used more general terminologies such as comma separated values (csv) instead of Panda. This improved the clarity and make the manuscript more general.
