# Supplementary material for: Machine learning for risk stratification of hypertensive disorders of pregnancy: Enhancing clinical efficiency in low-resource antenatal care in Tanzania
Source: PLOS Digit Health. 2026 Jul 2;5(7):e0001468. doi: 10.1371/journal.pdig.0001468 (PMC13327181; doi:10.1371/journal.pdig.0001468)
Supplement: Attachment — Submitted filename: Manuscript_Review_declet_1.docx [file pdig.0001468.s003.docx]

**REVIEW**

**Abstract**

**What was done well:**

The abstract is much better now. The authors clearly explain the trade-off between sensitivity and precision and describe the model as a high-sensitivity screening tool rather than a diagnostic system. They have clarified the dataset size by distinguishing total ANC visits, collapsed client records, the balanced training subset, and the independent validation dataset. This addresses my earlier concerns about transparency and generalizability. The tone is now more restrained and better suited to a proof-of-concept study.

**What remains to be done:**

The abstract still focuses too much on accuracy and AUC, which could mislead non-technical readers, given the very low precision. Although the trade-off is mentioned, adding a short phrase that quantifies the operational impact, like the number of false positives per true positive, would make the summary more realistic.

**Introduction**

**What was done well:**

The introduction is clearer and better organized. The authors now state the research gap: few ML studies use routine government ANC data at a national level in low-resource settings. Background on ANC reporting and UCS is shortened and moved, and the study objectives are introduced earlier. This improves narrative flow and highlights novelty.

**What remains to be done:**

The introduction would be improved by a clearer comparison with previous ML-based HDP studies, especially regarding performance metrics such as precision. Right now, the novelty is mostly about the data source and context, not about how the methods differ from existing models. A comparison is needed here.

**Methods**

**What was done well:**

This section is the most improved. The authors now give detailed descriptions of data preprocessing, visit collapsing, handling missing data, feature selection, class balancing, cross-validation, hyperparameter tuning, and independent validation. They clearly explain why they collapsed the longitudinal data, based on real-world needs. They also openly discuss the potential for data leakage when using BP-based outcome definitions. The modeling process is now reproducible and defensible.

**What remains to be done:**

Even with improved transparency, the outcome definition remains limited by using BP thresholds, which are also the top predictors. The authors mention this, but they should make it clearer that the model’s performance does not show true risk prediction. Instead, it should be seen as improved screening in settings with noisy measurements. This point is still unclear. In addition, for the correlation analysis to detect collinearity, it assumes that the continuous variables have a linear relationship. What if this assumption is wrong? The authors need to state how they checked this.

**Results**

**What was done well:**

The Results section is now more honest and informative. The authors present confusion matrices and ROC curves, assess calibration, and carefully analyze cases where results differ. They show and discuss the big drop in precision during real-world validation. Feature importance is also explained in clinical terms. This addresses earlier concerns about overfitting and selective reporting.

**What remains to be done:**

The almost-perfect cross-validation results on the balanced training data are still presented without sufficient caution. Overfitting is mentioned, but the manuscript should clearly say that these results do not prove the model is better. Instead, they are likely due to under-sampling and how the labels were created.

**Discussion and Conclusion**

**What was done well:**

The discussion is more balanced and improved. The authors now address the effects of low precision in resource-limited systems and give step-by-step recommendations for piloting, improving data quality, training, and policy integration. The conclusion presents the work as a proof-of-concept needing further validation.

**What remains to be done:**

The discussion still sometimes overstates the clinical benefits while failing to fully account for the additional burden on the system. Adding a clear statement that using the current model could greatly increase clinical workload would make the paper more credible and better aligned with implementation science.

I strongly believe addressing these concerns will improve the paper.

Thank you.
