# Supplementary material for: Machine learning for risk stratification of hypertensive disorders of pregnancy: Enhancing clinical efficiency in low-resource antenatal care in Tanzania
Source: PLOS Digit Health. 2026 Jul 2;5(7):e0001468. doi: 10.1371/journal.pdig.0001468 (PMC13327181; doi:10.1371/journal.pdig.0001468)
Supplement: Attachment — Submitted filename: ManuscriptPointByPointResponses.docx [file pdig.0001468.s006.docx]

# Point by point responses - Intro

Dear reviewers. We appreciate the time and effort which contributes to further improving our manuscript. Below, we summarized three feedback into three major themes which emerges across reviews, and also provides a point-by-point response in blue text.

**THEME 1: Outcome Definition and Circular Reasoning (addressing Reviewer 2, 4, and 5)**

Multiple reviewers raised concerns about using blood pressure both to define HDP and as the primary predictor. We acknowledge this fundamental limitation. As detailed in the revised Methods section (Outcome Definition), clinically confirmed HDP diagnoses were unavailable due to severe underreporting (<0.5% of records had completed diagnosis fields versus expected 5-10% prevalence). We have made three substantive revisions:

1. Reframed the study scope: The manuscript now explicitly states that the model predicts **BP-defined HDP only**, not clinically confirmed disease (Abstract & Methods section)
2. Added explicit data leakage discussion: Methods section now includes "Potential Data Leakage" subsection acknowledging this limitation
3. Revised all claims: Removed "early detection" language throughout including changing the title from “**Harnessing Digital Health and Machine Learning for Early Detection of Hypertensive Disorders of Pregnancy: Insights from low-resource settings, Tanzania**” to “**Machine Learning for Risk Stratification of Hypertensive Disorders of Pregnancy: Enhancing Clinical Efficiency in low-resource Antenatal Care in Tanzania**”. Now, our manuscript consistently use "risk stratification" and "screening support"

**THEME 2: Precision and Operational Burden (addressing Reviewer 2, 4, and 5)**

Reviewers appropriately highlighted that 14% precision means 86% of flagged women are false positives, with unexplored workflow implications. We have strengthened the manuscript by:

1. Inserted a new section which discuss implementation strategies to mitigate false-positive burden
2. Added a new section on clinical interpretability and potential added value.
3. Adding threshold optimization analysis: New Figure 9 (calibration plot) and Figure 10 (PPR curve) with corresponding text

**THEME 3: Temporal Data Handling (addressing Reviewer 4 and 5)**

Both Reviewer 4 and 5 noted that collapsing longitudinal data destroys temporal patterns. We agree this is a structural limitation necessitated by extreme visit sparsity. We have added:

4. Quantitative justification: Added median number of visits (1, IQR 1-2), percent with ≥2 visits (43.4%), and percent with ≥4 visits (6.3%) in Results (lines 150-155)
5. Explicit acknowledgment: Added to Limitations that temporally aware modeling would have excluded >93% of clients, introducing selection bias
6. Future directions: Added recommendation for temporally aware models as data quality and visit frequency improve"

# Point by Point Responses

Reviewer 2 – minor revision 
The revised manuscript is much improved and addresses the main concerns from the previous review. The abstract, methods, and results are now better, especially regarding how the dataset was built, how class imbalance was handled, how independent validation was done, and how the model is positioned as a high-sensitivity screening tool rather than a diagnostic system. The authors have improved the methods by clearly describing preprocessing, feature selection, cross-validation, and performance evaluation. The discussion now more openly addresses the impact of low precision in settings with limited resources and offers practical implementation recommendations. A few minor issues remain, such as the need to temper further the interpretation of near-perfect training performance and to more clearly explain the operational burden of false positives, but these do not detract from the overall value of the work.

Thank you for this thoughtful and constructive feedback on our manuscript. We appreciate the positive assessment of our work and acknowledge the two minor but important issues raised. We have carefully addressed both points as follows:

1. **Near-perfect training performance:** We agree that the near-perfect cross-validation metrics (accuracy=1.00, AUC=1.00) for XGBoost and Random Forest warranted tempering our interpretation. We have revised the manuscript to explicitly acknowledge that these perfect scores. We now frame these results as indicative of strong signal capture under ideal conditions, rather than implying the model is infallible.
2. **Operational burden of false positives:**  We have added explicit discussion of the resource implications of false positives, including the potential strain on overburdened health workers and the need for clear referral pathways. We clarify that our high-sensitivity approach is intended as a screening tool to flag patients for prioritized rather than immediate specialized review, and we outline mitigation strategies such as risk score tiering and clinical workflow integration.

**Abstract** 
What was done well: 
The abstract is much better now. The authors clearly explain the trade-off between sensitivity and precision and describe the model as a high-sensitivity screening tool rather than a diagnostic system. They have clarified the dataset size by distinguishing total ANC visits, collapsed client records, the balanced training subset, and the independent validation dataset. This addresses my earlier concerns about transparency and generalizability. The tone is now more restrained and better suited to a proof-of-concept study.

What remains to be done: 
The abstract still focuses too much on accuracy and AUC, which could mislead non-technical readers, given the very low precision. Although the trade-off is mentioned, adding a short phrase that quantifies the operational impact, like the number of false positives per true positive, would make the summary more realistic. 
Introduction

What was done well: 
The introduction is clearer and better organized. The authors now state the research gap: few ML studies use routine government ANC data at a national level in low-resource settings. Background on ANC reporting and UCS is shortened and moved, and the study objectives are introduced earlier. This improves narrative flow and highlights novelty.

What remains to be done: 
The introduction would be improved by a clearer comparison with previous ML-based HDP studies, especially regarding performance metrics such as precision. Right now, the novelty is mostly about the data source and context, not about how the methods differ from existing models. A comparison is needed here.

Thank you for continuing thoughtful engagement with our manuscript. We are pleased that the revisions have improved clarity, transparency, and tone, and we appreciate the recognition of our efforts to contextualize this as a proof-of-concept study in a low-resource setting. We have carefully considered the two remaining suggestions and have revised the manuscript accordingly:

**1. Abstract – quantifying operational impact:** We agree that the abstract's emphasis on accuracy and AUC, despite the sensitivity-precision trade-off being stated, may mislead non-technical readers. We have added a section on Performance trade-offs and operational implications, and provide quantification of the potential burden to the workforce.

**2. Introduction – comparison with previous ML-based HDP studies:** We have expanded the introduction to include a focused comparison with existing ML studies for HDP prediction, specifically we included references from PIERS-ML model (Montgomery-Csobaen et all), Li et al. achieving AUCs of 0.81 - 0.86 using electronic health records from a Chinese tertiary hospital; and Chen & Ma who reported accuracy of 84% and precision of 79% using clinical data from a single medical center and contrasting their reported precision metrics with ours.

Methods 
What was done well: 
This section is the most improved. The authors now give detailed descriptions of data preprocessing, visit collapsing, handling missing data, feature selection, class balancing, cross-validation, hyperparameter tuning, and independent validation. They clearly explain why they collapsed the longitudinal data, based on real-world needs. They also openly discuss the potential for data leakage when using BP-based outcome definitions. The modelling process is now reproducible and defensible.

What remains to be done: 
Even with improved transparency, the outcome definition remains limited by using BP thresholds, which are also the top predictors. The authors mention this, but they should make it clearer that the model’s performance does not show true risk prediction. Instead, it should be seen as improved screening in settings with noisy measurements. This point is still unclear. In addition, for the correlation analysis to detect collinearity, it assumes that the continuous variables have a linear relationship. What if this assumption is wrong? The authors need to state how they checked this.

Thank you for acknowledging the substantial improvements in transparency, reproducibility, and defensibility. We clarify below

**1. Outcome definition and interpretation of model performance:** We agree that despite acknowledging potential data leakage, the manuscript did not sufficiently emphasize that the model's performance reflects improved screening for BP-defined HDP under real-world measurement conditions rather than true risk prediction for clinically confirmed disease. We have revised the manuscript to make this distinction explicit, clarifying that our model should be interpreted as a tool to enhance detection of BP elevation patterns in noisy, low-visit data and not as a predictor of future HDP risk or undiagnosed clinical disease. This reframing better aligns with the study's contribution as an implementation science proof-of-concept.

**2. Linearity assumption in correlation analysis:** Thank you for noting that Pearson correlation assumes linear relationships between continuous variables. We have now explicitly stated that we assessed this assumption through visual inspection of scatterplots and calculation of Spearman's rank correlation coefficients (which do not assume linearity) for key variable pairs. The results were consistent with Pearson correlations, supporting the appropriateness of our approach. We have added this detail to the methods section.

Results 
What was done well: 
The Results section is now more honest and informative. The authors present confusion matrices and ROC curves, assess calibration, and carefully analyze cases where results differ. They show and discuss the big drop in precision during real-world validation. Feature importance is also explained in clinical terms. This addresses earlier concerns about overfitting and selective reporting.

What remains to be done: 
The almost-perfect cross-validation results on the balanced training data are still presented without sufficient caution. Overfitting is mentioned, but the manuscript should clearly say that these results do not prove the model is better. Instead, they are likely due to under-sampling and how the labels were created.

Thank you for acknowledging the improved honesty and informativeness of the Results section. We appreciate the recognition that our presentation of confusion matrices, ROC curves, discordant case analysis, and feature importance now provides a more transparent account of model performance.

We agree with the remaining concern regarding the near-perfect cross-validation results. Despite mentioning overfitting, the manuscript did not sufficiently emphasize that these perfect metrics are largely an artifact of our methodological choices, specifically random under-sampling to achieve class balance and the algorithmic outcome definition rather than evidence of superior model performance. We have revised the Results section to make this explicit, clarifying that the cross-validation results reflect performance on an artificially balanced, cleaned dataset and should not be interpreted as generalizable to real-world conditions. We now explicitly state that these metrics are expected given the study design and do not indicate that the model is "better" than alternatives.

Discussion and Conclusion 
What was done well: 
The discussion is more balanced and improved. The authors now address the effects of low precision in resource-limited systems and give step-by-step recommendations for piloting, improving data quality, training, and policy integration. The conclusion presents the work as a proof-of-concept needing further validation. 
 

What remains to be done: 
The discussion still sometimes overstates the clinical benefits while failing to fully account for the additional burden on the system. Adding a clear statement that using the current model could greatly increase clinical workload would make the paper more credible and better aligned with implementation science. 
I strongly believe addressing these concerns will improve the paper.

Thank you for noting the improved balance and clarity of the Discussion section. We appreciate the recognition that our step-by-step recommendations strengthen the implementation science framing of the work. We agree with the remaining concern: despite addressing low precision and its implications, the Discussion still overstates clinical benefits without fully accounting for the additional workload burden on an already overstretched health system. We have revised the Discussion to include an explicit, quantified statement about the potential workload impact of deploying the current model, and we have tempered claims about clinical benefits accordingly.

Reviewer 4 – major revision 
This is an important and well-motivated study. However, the use of a proxy HDP label, low precision, and lack of temporal modeling limit the reliability of the model’s predictions. The manuscript would benefit from more rigorous evaluation, expanded discussion of implementation challenges, and clearer framing of its current role as exploratory rather than deployment-ready.

The authors present a retrospective study applying machine learning (ML) to antenatal care (ANC) data collected from Tanzania’s Unified Community System (UCS) to predict hypertensive disorders of pregnancy (HDP). They evaluate several classifiers, selecting XGBoost for deployment, reporting an AUC of 0.95 and 90.95% accuracy on unseen data (n >120,000). The goal is to demonstrate how digital health data can support early detection of HDP in lowresource environments. Both prior reviewers agree that the paper demonstrates relevance, ambition, and technical effort, but also exhibits substantial limitations in clinical labeling, model evaluation, temporal data usage, and real-world implementation claims. This final review consolidates those points, offering a full technical evaluation.

Thank you for thorough technical evaluation and for recognizing the importance and motivation of our study. We appreciate the opportunity to clarify our rationale for using a proxy outcome definition and to address concerns about model reliability and implementation readiness. Below we respond to the major issues raised.

Major Issues 
1. Use of Proxy Outcome Definition (HDP) Without Clinical Validation. The outcome label for HDP is derived solely from systolic and diastolic blood pressure thresholds (≥140/90 mmHg), without confirmation from diagnosis fields, medication data, or clinician annotations. This label construction is acknowledged as a proxy but remains fundamentally weak for building clinically trustworthy prediction models. Impact: This creates a risk of label noise, undermines model evaluation, and raises concerns about internal validity. Recommendation: While data limitations are acknowledged, the paper must reframe its scope from “early detection of HDP” to “classification of blood pressure risk states,” and clearly state the limits of proxy labelling in both methods and conclusions. 
 

We agree with the reviewer that using a proxy outcome defined by BP thresholds is a fundamental limitation. As detailed in the revised Methods section (Outcome definition), clinically confirmed HDP diagnoses were unavailable due to severe underreporting (<0.5% of records had completed diagnosis fields versus expected 5-10% prevalence).

To address this concern, we have:

1. Reframed the manuscript to explicitly state that the model predicts **BP-defined HDP only** (Abstract, Methods, Limitations)
2. Added a 'Potential Data Leakage' subsection acknowledging circularity
3. Removed all claims of 'early detection' and replaced with 'risk stratification'

The proxy definition was a pragmatic necessity. Requiring clinically confirmed diagnoses would have made model development impossible in this setting. We now position this as a proof-of-concept that demonstrates feasibility and incentivizes improved data capture, with prospective validation using clinical outcomes as the essential next step.

2. Severe Precision Deficit (14%) With Limited Operational Mitigation Both reviews noted that the model exhibits a 14% precision—indicating that most positive predictions are false positives. The authors attempt to justify this through high recall and potential for early warning, but there is no quantitative or operational analysis of the downstream clinical impact of such a high false positive burden in resourceconstrained settings. Impact: This challenges the model’s practical deployability and user trust. Recommendation: Authors must include a risk-benefit discussion, propose specific triaging protocols, or demonstrate via simulation how false positives would be handled without overwhelming staff or infrastructure. 

Response:

Thank you for comment on implementation challenge. We agree that a 14% precision score, resulting in approximately six false positives for every true positive, poses substantial operational concerns in resource-constrained settings. Our justification follows

**Prioritize sensitivity over precision:** In the context of hypertensive disorders of pregnancy in Tanzania, where HDP accounts for approximately 34% of direct obstetric deaths, the consequence of a false negative (missed case) can be maternal mortality, while the consequence of a false positive is additional clinical workload. Our design choice to prioritize sensitivity (recall = 100%) reflects a deliberate risk-benefit calculation: it is clinically preferable to over-alert and investigate than to miss a potentially fatal case. However, we fully acknowledge that this trade-off imposes a burden on an already overstretched health system.

**Quantifying the operational burden:** In the validation dataset (N=120,232), the model flagged 12,603 women as high-risk, of whom only 1,725 had BP-defined HDP, yielding 10,878 false positives. To contextualize this:

- Per true positive: 6.3 false positives require additional assessment
- Per 100 ANC clients: approximately 10 would be flagged as high-risk, but only 1-2 would have BP-defined HDP
- In a busy clinic seeing 50 ANC clients daily: the model would generate 5 high-risk flags per day, of which 4 would be false positives requiring follow-up

We have revised the manuscript to frame the model explicitly as a triage tool for prioritization rather than a diagnostic system. We have included specific paragraph (in the discussion section) for clinical interpretability and potential added value as well as implementation strategies to mitigate false-positive burden. Our goal is not to replace clinical judgment but to help overburdened clinicians focus attention on the women most likely to benefit from closer monitoring.

3. Flattening of Temporal Data is a Structural Limitation. The decision to aggregate longitudinal ANC visit data into static per-patient feature vectors eliminates critical gestational progression patterns, which are central to HDP pathophysiology. This limitation is acknowledged but not sufficiently addressed. Impact: This structurally limits predictive accuracy and masks important trends such as rising BP trajectories or changes in proteinuria over time. Recommendation: Authors should either experiment with temporally aware models or provide structured temporal feature engineering (e.g., BP slopes, deltas) and fully articulate the implications of the current model’s static nature. 

Thank you for this observation. The decision to flatten longitudinal ANC visit data into static per-patient vectors was indeed a structural compromise, and we agree that it eliminates critical gestational progression patterns that are central to HDP pathophysiology. It is indeed correct that the temporal patterns such as rising blood pressure trajectories or changes in proteinuria over time are clinically significant for HDP detection. However, our data suffered severe limitations in temporal depth where the median number of visits per client was 1 (IQR: 1–2), clients with ≥2 visits were 43.4% (81,325/187,438), clients with ≥4 visits were only 6.3% (11,795/187,438) and clients with ≥8 visits (WHO recommended minimum) were 0.08% (157/187,438)

With only 43% of clients having more than one visit, and fewer than 7% having sufficient visits to construct meaningful trajectories, any temporally aware modelling approach would have been limited to a small, non-representative subset of the population. Models trained on this subset would likely suffer from selection bias, as women with more visits may differ systematically from those with fewer visits (e.g., higher-risk pregnancies receiving more attention, or women with better healthcare access).   We have expanded the section under data quality and added Table 2 that shows missingness rates for key features in the original dataset before collapse

4. Lack of External or Prospective Validation While the model is tested on a large test cohort (n = 120,232), no prospective, geographic, or temporal external validation is performed. Furthermore, predictions are not linked to actual delivery or maternal outcomes. Impact: Limits generalizability and weakens claims about readiness for deployment or policy use. Recommendation: The manuscript should temper claims regarding deployment and emphasize the need for a formal prospective validation study. A pilot phase or follow-up outcome analysis would significantly strengthen future versions. 

Thank you for highlighting this critical limitation. We agree that the absence of prospective, geographic, or temporal external validation and the lack of linkage to actual delivery or maternal outcomes limits generalizability and means the model is not yet deployment-ready. Our study design was limited to several contextual and resource constraints, which prevented this. These includes,

- **Funding and timeline:** This study was funded through a two-year Grand Challenges grant from the Bill & Melinda Gates Foundation. The funding period constrained us to a retrospective analysis of existing data, with no resources allocated for prospective data collection or follow-up of maternal outcomes. While we were able to access data spanning beyond the two-year period through our collaboration with the Ministry of Health, this access was limited to existing retrospective records.

- Health facility access: Despite the large dataset (120,232 records for validation), these data originated from a limited number of health facilities where the UCS digital system was actively implemented. Geographic external validation across a broader range of facilities was not feasible within the project scope.

- Data linkage challenges: The reviewer correctly identifies the absence of linkage to delivery or maternal outcomes as a weakness. In Tanzania (as in most low-resource settings) ANC data and delivery outcome data are collected in separate records (often paper-based) and cannot be reliably linked at the individual level due to the absence of unique patient identifiers. Even within the UCS digital platform, which captures both ANC and delivery data, we experienced challenges to link a woman's ANC records to her delivery outcomes. This structural limitation is well-documented in LMIC health information systems [17,19] and represents a fundamental barrier to outcome validation that this study alone could not overcome.

5. Omission of Missing Data and Data Quality Protocols There is no description of missing data handling, which is critical in LMIC digital health datasets. Features such as glucose, protein in urine, and syphilis status are often missing or inconsistently recorded. Impact: Compromises the transparency, reproducibility, and robustness of the model. Recommendation: Authors must report missingness rates per feature, describe imputation or exclusion methods, and assess model sensitivity to data completeness.

Thank you for highlighting this important omission. We added Table2 (and corresponding text) which details the missingness rates for key features in the original dataset. In addition, we deliberately chose not to impute missing values. Imputation in this context could introduce bias by assuming patterns that may not hold in settings where data are missing not at random (e.g., sicker patients may be more likely to have certain tests ordered). Instead, we used listwise deletion (complete case analysis) for model development. Furthermore, records missing any of the final seven features were excluded from the analytic dataset. This resulted in the exclusion of 136,864 records (73% of the original 187,438 clients).

Minor Issues

Model threshold selection and calibration are not discussed. Threshold optimization (e.g., Youden Index, F1-maximization) under class imbalance should be detailed.

Thank you for highlighting the need to discuss threshold optimization and calibration. Because of very high class imbalance, we did not performYouden’s Index calculation but intentionally calibrated the decision threshold to prioritize 100% sensitivity. In additional, we have included a calibration plot and Brier score and corresponding text in the results section

Feature definitions are ambiguous in several places (e.g., “ANC risk flag,” “partner HIV status”); authors should provide a data dictionary or glossary.

Thank you for this key observation. We have revised the manuscript to remove any ambiguous featured definition.

Use of ROC curves is insufficient under high class imbalance; precision-recall curves should be included.

Thank you for this methodological suggestion. We have added Precision-	Recall 	curve (Figure 8) and corresponding text in Results

SHAP plots are informative but need clearer axis labels and clinical interpretation guidance.

Thank you. We have revised Figure 5 caption and added clinical interpretation text in Results

Terminological ambiguity between “HDP” and “gestational hypertension” must be resolved; either clarify or limit the scope of what the model is predicting.

Thank you. Revised throughout to consistently use "HDP"

Figures and Tables 
Figures are well constructed but need improvements in labeling. A confusion matrix and precision-recall curve should be included. Tables summarizing missing data rates and class distribution across training, validation, and test sets are missing and should be provided.

Thank you. Confusion matrix was already there. We have added precision-recall figure and a table summarizing missing data by features.

Methods Structurally adequate but lacking in key details:

No account of data cleaning or outlier handling

No missing data methodology

No description of feature engineering or variable encoding

- Sampling strategy for balancing classes is not defined Study Design Appropriate as a retrospective feasibility study. However, the title and framing imply implementation readiness, which the current evidence does not support. A prospective or hybrid study design would be a more suitable next step.

Thank you for the constructive feedback on our methods section. We have substantially expanded the description of our methodological approach to address each of the points raised. We have expanded the section on data processing and feature engineering, added a section on missing data handling, feature engineering, and sampling strategy.

Any Experiments Missing?

·       External or temporal validation experiment

·       Sensitivity analysis of label definitions

·       Subgroup analysis by gestational age or risk tier

- Threshold tuning experiment Statistics Baseline metrics are acceptable, but calibration (e.g., Brier score, reliability diagrams) is not assessed. Confidence intervals for key metrics (AUC, precision, recall) are missing.

Thank you for these thoughtful suggestions regarding additional experiments that could strengthen the analysis. Below are responses corresponding to the point raised.

**External validation experiments** 
We agree that external validation is essential for establishing generalizability. While we could not perform geographic external validation across different regions or health systems, we did conduct temporal validation by training the model on historical data (2020–2023) and testing on a fully independent dataset from a subsequent time period (April–May 2024, N=120,232). This temporal split ensures that the validation data were not used in any way during model development and provides evidence of performance stability over time. However, we acknowledge that Geographic external validation (e.g., testing in regions not represented in the training data) was not performed. The temporal validation dataset, while independent, came from the same health facilities and regions as the training data

**Sensitivity analysis of label definitions** 
Our outcome label was algorithmically defined using BP thresholds (≥140/90 mmHg). We recognize that this definition may be sensitive to the specific threshold chosen and does not capture the full clinical spectrum of HDP. This was necessary due to the absence of clinically confirmed HDP diagnoses in the routine data (as detailed in our response to Question 1), we could not conduct a formal sensitivity analysis comparing model performance against different gold standards (e.g., clinically diagnosed HDP vs. BP-defined HDP).

To overcome this limitation, we assessed the model's sensitivity to the BP threshold itself by examining discordant cases where model predictions diverged from BP threshold-based labels (reported in Results) and demonstrating that the model identifies cases missed by single-visit BP checks, suggesting it captures additional signal beyond the threshold definition. We have added explicit acknowledgment of this limitation:

**Subgroup analysis by gestational age or risk tier** 
We agree that subgroup analyses would provide valuable insights into model performance across clinically relevant populations. However, the gestational age variable was not retained in the final feature set. Our feature selection process using Recursive Feature Elimination (RFE) identified systolic BP, diastolic BP, and syphilis status as the most parsimonious set. Gestational age was eliminated due to limited predictive contribution in the context of our collapsed, non-temporal dataset. Without gestational age in the model, meaningful subgroup analysis by trimester or gestational age windows is not feasible. In addition, our current model produces binary classifications (risk/no risk) based on a single threshold. We have not yet developed or validated a multi-tier risk stratification system (e.g., low/moderate/high)

**Threshold Tuning Experience**

Thank you for this highlight. Our revised manuscript now includes clinical threshold optimization and triage utility.

Calibration Assessment (Brier Score, Reliability diagrams)

Our revised manuscript does include calibration assessment indicating Brier score (0.08), calibration slope (0.94) and calibration intercept (-.0.08). We have included (**Figure 9)** (calibration plot) showing the relationship between predicted probabilities and observed HDP prevalence.

Results and Data: Support Conclusions? 
Partially. The technical performance is adequate, but the clinical conclusions and implementation claims outpace the underlying evidence.

Thank you for this critical and fair assessment. We agree that our initial framing overstated the clinical conclusions and implementation readiness relative to the underlying evidence. We have revised the manuscript to ensure that our conclusions are appropriately tempered and grounded in the study's

All Data Accessible? No data or code sharing statement is present. Authors must clarify availability of de-identified data and model artifacts for reproducibility.

Thank you for this highlight. The data supporting this study is not publicly available due to the presence of potentially identifiable information and restrictions under institutional and national data protection policies. Access may be granted upon reasonable request and with prior approval from the Ministry of Health, Tanzania. Interested researchers may contact the Ministry to initiate the data-access process, which is subject to all required ethical and administrative approvals. But the source code for the model is published in GitHub, and we provided a reference under code reproducibility paragraph.

References 
Sufficient for background and methodology. Authors may consider referencing clinical HDP prediction tools (e.g., fullPIERS, PREP-S) and ethical implications of ML in maternal health.

Thank you for this suggestion. We have now added Montgomery-Csobaen et all and von Dadelszen P (fullPIERS, PIERS models) as an additional reference to our manuscript.

Also, we have added below references for ethical consideration in ML

- Rajkomar A, Hardt M, Howell MD, Corrado G, Chin MH. Ensuring fairness in machine learning to advance health equity. Annals of Internal Medicine. 2018.
- Wiens J, Saria S, Sendak M, et al. Do no harm: a roadmap for responsible machine learning for healthcare. Nature Medicine. 2019.
- World Health Organization. Ethics and governance of artificial intelligence for health. WHO. 2021.

Title 
Accurate but overstated. Given the limitations in clinical outcome validation, a more accurate title would replace “Early Detection of Hypertensive Disorders of Pregnancy” with “Machine Learning Risk Classification of Elevated Blood Pressure in ANC Records.”

# Thank you for this highlight. We have changed the title to not over-state the claims. The revised title is **Machine Learning for Risk Stratification of Hypertensive Disorders of Pregnancy: Enhancing Clinical Efficiency in low-resource Antenatal Care in Tanzania**

Reviewer 5 – reject 
Recommendations: 
 
1. The study uses blood pressure measurements to both define HDP and as the primary predictor variables, creating inherent circular reasoning. This undermines the core premise that machine learning adds value beyond simple threshold-based screening. The authors should either reframe this as a digital infrastructure feasibility study, obtain clinically confirmed HDP diagnoses for proper validation, or demonstrate true temporal prediction by using early visit data to predict HDP at later visits. 

Response: Thank you for this key observation. This is indeed the most fundamental limitation of our study, and we appreciate the opportunity to clarify our rationale, the steps we have taken to mitigate this concern, and how we have reframed the manuscript to accurately reflect what the model does and does not demonstrate.

As detailed in our response to Reviewer 4 (Question 1) and our themed responses, clinically confirmed HDP diagnoses were unavailable in the routine data for several reasons:

1. Underreporting - <0.5% of records had completed HDP diagnosis fields vs. expected 5–10% prevalence
2. Delayed entry - Data entered after clinical events often miss diagnoses
3. Labour ward separation - HDP cases managed in labour wards could not be linked to ANC records
4. Reporting reluctance - Providers may avoid documenting adverse outcomes due to facility performance concerns
5. Insufficient visit frequency hindered temporal assessment --> Median number of visits per client was 1; only 43% of the clients had more than 2 visits.

In this context, relying on documented diagnoses would have made model development impossible. The proxy definition was a pragmatic necessity, not a methodological preference.

What we have done to address this limitation,

1. Reframed the study scope: The manuscript now explicitly states that the model predicts **BP-defined HDP only**, not clinically confirmed disease
2. Explicit defined the model scope as the BP-defined HDP only
3. Demonstrated added value beyond simple threshold
4. Acknowledged that temporal prediction was not possible.
5. Recommended prospective validation with clinical outcomes

2. The title and abstract emphasize early detection, but the study design only demonstrates concurrent classification using current blood pressure readings, not prediction before clinical onset. The discordant case analysis shows that false positives have blood pressure just below diagnostic thresholds (136.5 vs 140 mmHg), which represents borderline detection rather than early warning. Authors should either demonstrate genuine early prediction using data from first trimester visits to predict later HDP, or reframe their contribution as "enhanced screening support" rather than early detection.

# Thank you for this important distinction. We have accepted the suggestion and chose to reframe our contribution as **"enhanced screening support"** and **"risk stratification".** We changed the title to “**Machine Learning for Risk Stratification of Hypertensive Disorders of Pregnancy: Enhancing Clinical Efficiency in low-resource Antenatal Care in Tanzania**

# 3. Excluding 73% of records (136,864 of 187,438) due to missing data represents a critical threat to generalizability that is inadequately discussed. Authors must provide a comparison table showing whether excluded records differ systematically from included records, analyze missingness patterns (MCAR vs MAR vs MNAR), and justify why complete-case analysis was chosen over imputation methods. This selection bias could severely limit real-world applicability and must be transparently reported with implications for model deployment.

Thank you for suggesting this rigorous analysis. We have added Table 2 detailing missingness rates for key features in the original dataset. In additional, we performed separate statistical tests for numerical variables (t-test) and categorical variables (chi-square), obtaining actual p-values. The findings not only confirm and strengthen our earlier interpretation but also reveal important nuances about the excluded population. Specifically, we observed highly significant differences (p < 0.001 for all comparisons) between excluded and included records. The missing data pattern is not random; rather, it reflects systematic undertesting, with nearly half of excluded women lacking proteinuria and glucose testing altogether. This likely mirrors clinical practice, where women presenting urgently, arriving late, or experiencing complications often receive treatment before testing, leaving them underrepresented in the dataset.

4. While the authors justify prioritizing sensitivity, the 14% precision means 86% of flagged women do not actually have HDP, and the clinical workflow implications are unexplored. The manuscript needs to include baseline HDP prevalence in Tanzanian populations, describe the clinical pathway for managing flagged cases, estimate resource costs of investigating 12,603 alerts to find 1,725 true cases, and compare performance against existing standard-of-care detection rates. A sensitivity analysis showing model performance at different probability thresholds would also strengthen the clinical applicability discussion. 

We thank the reviewer for highlighting the practical implications of low precision. We have added baseline HDP prevalence in Tanzania in the introduction. In additional, we expanded our discussion to include our deliberate move to prioritized sensitivity (100%) over precision (14%), accepting 6.3 false positives per true positive to ensure no cases are missed. This trade-off must account for current standard-of-care (single-visit BP) which detects only 60 - 70% of HDP cases, missing an estimated 11 - 15 women per 1,000 attendees. Our model could potentially identify an additional 22,000 to 30,000 cases annually nationwide, at a cost of 75 minutes of additional clinical time in busy clinics.

5. The decision to collapse multiple ANC visits into single records using different aggregation rules (means for BP, maximum for gestational age, most recent for categorical variables) appears arbitrary and destroys potentially valuable temporal patterns. Authors should provide explicit justification for each aggregation method, consider time-series approaches or visit-specific models as alternatives, and at minimum conduct sensitivity analyses using different aggregation strategies. This is particularly important since BP trends over pregnancy are clinically meaningful for HDP risk assessment.

Thank you for this methodological critique. The decision to collapse longitudinal data was not arbitrary but necessitated by extreme visit sparsity in our dataset, which precluded meaningful temporal analysis. Our data revealed severe limitations in temporal depth where the median number of visits per client was 1 (IQR: 1–2), clients with ≥2 visits were 43.4% (81,325/187,438), clients with ≥4 visits were only 6.3% (11,795/187,438) and clients with ≥8 visits (WHO recommended minimum) were 0.08% (157/187,438)

With only 43% of clients having more than one visit, and fewer than 7% having sufficient visits to construct meaningful trajectories, any temporally aware modelling approach would have been limited to a small, non-representative subset of the population. Models trained on this subset would likely suffer from selection bias, as women with more visits may differ systematically from those with fewer visits (e.g., higher-risk pregnancies receiving more attention, or women with better healthcare access).   

 
6. The validation dataset (April-May 2024) comes from the same population, facilities, and data collection system as the training data (pre-April 2024), which tests temporal stability rather than true generalizability. Authors should add geographic or facility-level cross-validation, report stratified performance by region and patient demographics, and explicitly discuss how the model would perform in facilities without UCS infrastructure. Without this, claims about scalability and broader implementation remain unsubstantiated.

Thank you for this distinction between temporal validation and true geographic generalizability. We agree that our validation dataset, while temporally independent (April–May 2024, N=120,232), draws from the same facilities and regions as the training data. This demonstrates temporal stability but not geographic transportability. We have therefore revised our claims accordingly.

The manuscript now explicitly states that geographic external validation across different regions, health systems, or facilities without UCS infrastructure was not performed, and that the temporal validation dataset, while independent, originated from the same health facilities and regions as the training data. This means the model's performance in facilities with different populations, data quality profiles, or clinical workflows remains unknown. However, an important contextual consideration is that the Ministry of Health has committed to continued nationwide deployment of the UCS platform as the digital backbone for ANC services, particularly in low-resource settings with limited staffing. This planned expansion means that future implementations will benefit from the same data structure, system integration, and standardized data elements. As UCS scales to new facilities and regions, the model can be deployed with minimal adaptation, leveraging consistent data definitions and workflows.

7. Given that BP dominates the model (SHAP values +7.94 and +7.62) and the outcome is defined by BP thresholds, the manuscript must explicitly demonstrate what additional value machine learning provides over simple clinical protocols. Authors should conduct an ablation study showing model performance when BP features are excluded entirely, compare detection rates against current Tanzanian ANC guidelines, and provide concrete examples of cases where the integration of BMI, proteinuria, and other factors identified at-risk women who would have been missed by BP screening alone. This is essential to justify the added complexity and resource investment of ML deployment.

Thank you for this important comment. While blood pressure (BP) is central to both the clinical definition of hypertensive disorders of pregnancy and the model predictions, the machine learning (ML) model does not simply replicate the standard BP threshold used in routine screening. Instead, the model uses the full range and subtle variation in BP measurements together with other risk factors to identify patterns of elevated risk that may not yet meet the clinical diagnostic cutoff. In practice, this means the model can detect women with borderline or gradually increasing BP levels (e.g., values below 140/90) whose overall risk becomes elevated when combined with other variables such as BMI, proteinuria, age, or infection status. Compared with current Tanzanian ANC guidelines that rely primarily on a BP cutoff of ≥140/90, our model identifies additional women at risk whose BP is still below the diagnostic threshold at the time of screening. The advantage of the ML approach is therefore not to replace BP measurement, but to use the continuous BP signal together with other clinical indicators to generate a probabilistic risk score. This allows the model to capture marginal BP differences and multi-factor risk patterns that are not accounted for by traditional cut-point–based protocols, thereby enabling earlier risk stratification and potentially more timely monitoring within routine antenatal care systems.

We believe these revisions strengthen the manuscript's scientific rigor and practical relevance. All changes are clearly marked in the revised manuscript.
