# Supplementary material for: Machine learning for risk stratification of hypertensive disorders of pregnancy: Enhancing clinical efficiency in low-resource antenatal care in Tanzania
Source: PLOS Digit Health. 2026 Jul 2;5(7):e0001468. doi: 10.1371/journal.pdig.0001468 (PMC13327181; doi:10.1371/journal.pdig.0001468)
Supplement: Peer review history

# Peer review: Decision Letter 0

**DOI:** 10.1371/journal.pdig.0001468.r001

**Authors:** Dhiya Al-Jumeily OBE, Anna Khalemsky

19 Sep 2025

PDIG-D-25-00485

Harnessing Digital Health and Machine Learning for Early Detection of Hypertensive Disorders of Pregnancy: Insights from low-resource settings, Tanzania

PLOS Digital Health

Dear Dr. Lyatuu,

Thank you for submitting your manuscript to PLOS Digital Health. After careful consideration, we feel that it has merit but does not fully meet PLOS Digital Health's publication criteria as it currently stands. Therefore, we invite you to submit a revised version of the manuscript that addresses the points raised during the review process.

Please submit your revised manuscript within 60 days Nov 18 2025 11:59PM. If you will need more time than this to complete your revisions, please reply to this message or contact the journal office at digitalhealth@plos.org. When you're ready to submit your revision, log on to https://www.editorialmanager.com/pdig/ and select the 'Submissions Needing Revision' folder to locate your manuscript file.

Please include the following items when submitting your revised manuscript:

* A rebuttal letter that responds to each point raised by the editor and reviewer(s). You should upload this letter as a separate file labeled 'Response to Reviewers'. This file does not need to include responses to any formatting updates and technical items listed in the 'Journal Requirements' section below.

* A marked-up copy of your manuscript that highlights changes made to the original version. You should upload this as a separate file labeled 'Revised Manuscript with Track Changes'.

* An unmarked version of your revised paper without tracked changes. You should upload this as a separate file labeled 'Manuscript'.

If you would like to make changes to your financial disclosure, competing interests statement, or data availability statement, please make these updates within the submission form at the time of resubmission. Guidelines for resubmitting your figure files are available below the reviewer comments at the end of this letter.

We look forward to receiving your revised manuscript.

Kind regards,

Dhiya Al-Jumeily OBE

Section Editor

PLOS Digital Health

Leo Anthony Celi

Editor-in-Chief

PLOS Digital Health

orcid.org/0000-0001-6712-6626

**Journal Requirements:**

1. Please amend your detailed Financial Disclosure statement. This is published with the article. It must therefore be completed in full sentences and contain the exact wording you wish to be published.

i. State the initials, alongside each funding source, of each author to receive each grant.

ii. State what role the funders took in the study. If the funders had no role in your study, please state: “The funders had no role in study design, data collection and analysis, decision to publish, or preparation of the manuscript.”

2. Please ensure that your Ethics Statement is available in its entirety at the beginning of your Methods section, under a subheading 'Ethics Statement'.

3. Please upload separate figure files in .tif or .eps format. Also, remove the figures from your manuscript file but keep the legends.

For more information about figure files please see our guidelines:

https://journals.plos.org/digitalhealth/s/figures

https://journals.plos.org/digitalhealth/s/figures#loc-file-requirements

4. Please provide an Author Summary. This should appear in your manuscript between the Abstract (if applicable) and the Introduction, and should be 150–200 words long. The aim should be to make your findings accessible to a wide audience that includes both scientists and non-scientists. Sample summaries can be found on our website under Submission Guidelines:

https://journals.plos.org/digitalhealth/s/submission-guidelines#loc-parts-of-a-submission

5. For studies involving third-party data, we encourage authors to share any data specific to their analyses that they can legally distribute. PLOS recognizes, however, that authors may be using third-party data they do not have the rights to share. When third-party data cannot be publicly shared, authors must provide all information necessary for interested researchers to apply to gain access to the data. (https://journals.plos.org/plosone/s/data-availability#loc-acceptable-data-access-restrictions)

For any third-party data that the authors cannot legally distribute, they should include the following information in their Data Availability Statement upon submission:

1) A description of the data set and the third-party source

2) If applicable, verification of permission to use the data set

3) Confirmation of whether the authors received any special privileges in accessing the data that other researchers would not have

4) All necessary contact information others would need to apply to gain access to the data

If the reviewer comments include a recommendation to cite specific previously published works, please review and evaluate these publications to determine whether they are relevant and should be cited. There is no requirement to cite these works unless the editor has indicated otherwise.

**Additional Editor Comments (if provided):**

Reviewer #1:

Reviewer 1: Recommendation - reject

This paper uses ML models on a unique dataset to predict Hypertensive Disorders on Pregnant women in Tanzania, leveraging a databased developed by the Government of Tanzania. The manuscript can be improved as follows:

Major comments:

1. It is unclear why we should use machine learning to detect hypertension, when. The hypertension is defined by a simple threshold of blood pressure measurements, and blood pressure is available to the model. The authors should explain better their reasoning for this.

2. The study design has an important problem of data leaks. The outcome is defined from the features of interests.

3. The dataset is highly imbalance, which makes accuracy a suboptimal metric for evaluating performance.

4. Confusion metrics is not provided

5. The ROC curve used for calculating the AUC is not provided

Overall, it is unclear the benefit of this approach, and how it contributes to the state-of-the-art. The authors should provide further information on why their approach is novel, and why.

Minor comments:

6. Figure 1 has no x-axis label

7. Page 3, when mentioning "In the context of maternal health, numerous studies have successfully developed and validated ML models", please cite these studies.

Reviewer #2:

Reviewer 2: Recommendation – major review

Abstract

This abstract takes on a critical issue, Tanzania’s persistently high maternal mortality, and does so in a way that feels timely and innovative. Using digital antenatal care records and machine learning to predict hypertensive disorders of pregnancy is a strong framing, and the sheer scale of the dataset (300,000 visits) immediately makes the work stand out. Highlighting the comparison of multiple models before choosing XGBoost also signals methodological care, which is a plus for readers.

At the same time, the way the results are presented feels a little unbalanced. The abstract emphasizes the high accuracy and AUC, but the precision (14%) and F1-score (24%) tell a very different story. Those numbers suggest the model isn’t yet reliable enough for clinical use, and that point is downplayed. A quick acknowledgment that the model favors sensitivity at the cost of precision would help set more realistic expectations about its utility in a low-resource clinical environment.

There’s also some confusion in the dataset description that could use improvement. The authors say they analyzed 300,000 records, but then note only 3,788 were in the training dataset with a 50/50 distribution. That sounds like some balancing or sub-sampling was done, but it isn’t explained. Even a short clarification in the abstract would prevent readers from questioning whether the results are generalizable. Similarly, mentioning how missing values were handled, or whether repeated visits from the same women were included, would add a lot of credibility.

Lastly, the conclusion leans a bit too optimistic. It’s fair to say that AI can support earlier detection and potentially reduce maternal mortality, but the abstract should frame these results as proof-of-concept rather than ready-to-deploy. The mention of data quality is on point, but it could go further, scaling models like this will also require stronger infrastructure, training, and policy support. Balancing optimism with realism would make the abstract much stronger and more trustworthy.

Introduction

The introduction does a good job of framing the study within the broader context of machine learning applications in maternal health. The authors clearly highlight that ML has been used to predict conditions such as pre-eclampsia, gestational diabetes, and preterm labor, and they link these efforts to the growing availability of digital health data. The reference to WHO recommendations on early detection is particularly useful, since it grounds the study in internationally recognized priorities for improving maternal health outcomes. Overall, the opening establishes that the topic is both relevant and timely.

At the same time, the introduction feels too expansive and could be made more focused. The section on antenatal care reporting provides helpful background but it reads more like a health systems overview than part of a research introduction. It would be stronger if it were condensed and framed in terms of why gaps in ANC reporting matter for early detection of hypertensive disorders of pregnancy. The same is true for the long description of the Unified Community System. While the details are interesting, not all of them are necessary in the introduction. Some information could be moved to the Methods section, while the introduction could highlight more directly how the UCS enables new possibilities for predictive modeling in Tanzania.

The study objectives are well defined but they appear too late in the introduction. Presenting them earlier, right after describing the maternal health burden and the potential role of ML, would give readers a clearer sense of direction. As written, the objectives risk being overshadowed by long background sections. A sharper structure would move from problem (maternal mortality and inadequate ANC data), to opportunity (ML models applied to routine digital data), to the specific research goals. This would keep readers oriented and make the originality of the study stand out more clearly.

Finally, the introduction would be stronger if it more explicitly identified the research gap this study is addressing. The authors mention that ML has been applied to maternal health outcomes, but it is not clear what has been missing from past studies that this paper uniquely contributes. Is it the scale of the dataset, the Tanzanian context, or the integration with a national digital health system? Stating this gap directly would make the novelty of the work more visible. Similarly, acknowledging early on that routine data may have quality issues would strike a more balanced tone and prepare readers for the practical challenges that the study addresses.

Methods

The Methods section provides a good overview of the study design, dataset, and analytic approach. The use of a retrospective cohort design with routine antenatal care data is appropriate given the study objectives, and the description of the Unified Community System adds valuable context on how the data was collected. The attention to data cleaning and preprocessing, including the conversion of variable formats, duplicate removal, and thoughtful aggregation of longitudinal records into cross-sectional format, shows care in preparing the dataset for analysis. Involving obstetricians and public health experts in feature selection is also a strength, as it helps ensure clinical relevance.

At the same time, several aspects of the methods would benefit from additional clarity and detail. For example, the decision to collapse repeated ANC visits into a single record per client may simplify the modeling process, but it also risks losing important temporal information that could influence hypertensive risk. A brief justification for this choice, or at least an acknowledgment of the trade-off, would make the approach more transparent. Similarly, while the authors note that Pearson correlation was used to explore the relationship between predictors and the outcome, correlation may not be the best measure for categorical or non-linear relationships. Providing more detail on why this method was chosen, and whether other feature selection techniques were considered, would strengthen confidence in the modeling pipeline.

The description of the outcome definition is a critical part of this study, and the authors are right to highlight the challenges with underreporting of hypertensive disorders in the dataset. However, the reliance on a single threshold-based calculation to define cases may oversimplify clinical reality. Blood pressure measurements in routine ANC settings can be variable due to equipment quality, staff training, or measurement error. A discussion of how these potential inaccuracies might affect case definition, or whether multiple readings per patient were considered, would make the outcome measure more robust.

Finally, the modeling approach is described in broad strokes but lacks important details for reproducibility. For instance, it is not clear whether cross-validation was used during model training, what hyperparameter tuning was performed, or whether class imbalance was addressed explicitly. Reporting accuracy alone is not sufficient, since accuracy can be inflated in imbalanced datasets; the inclusion of precision, recall, F1-score, and calibration metrics would make the evaluation more complete. The section on model application is a useful addition, showing how the model was tested on new UCS data, but it could go further by explaining how the model’s predictions compared to traditional case definitions and what implications this has for real-world adoption.

Results

The Results section is thorough and presents a wide range of analyses, from descriptive statistics through model validation and feature importance. The descriptive findings are helpful, especially the breakdown of ANC visits before and after collapsing records into individual clients. This makes it clear how much information is lost when visits are aggregated and highlights an important contextual point for interpreting the models. The histogram of gestational age and the correlation heatmap add useful visualization, showing both the distribution of visits and the strength of associations with hypertensive disorders of pregnancy.

At the same time, there are several areas that would benefit from refinement. The correlation results are described as significant, but Pearson correlation is a limited tool for understanding complex relationships between predictors and outcomes, especially when variables are non-linear or categorical. More robust feature selection methods could be mentioned, or at least an acknowledgment that correlation is a preliminary measure. In addition, the decision to balance the dataset by downsampling or resampling to achieve equal risk and no-risk groups should be described in more detail, as this step has a major influence on model performance and interpretability.

The model training and validation results appear strong at first glance, with very high cross-validation scores across all classifiers. However, the near-perfect accuracy shown in Table 2 (100 percent across precision, recall, and F1-score in the small test dataset) raises concerns about overfitting. This becomes evident when the model is applied to new UCS data, where performance drops considerably, especially in precision (14 percent) and F1-score (24 percent). These results deserve a more critical discussion in the Results section itself, not just later in the paper. Presenting calibration curves, confusion matrices, or a breakdown of false positives versus false negatives would make the performance evaluation more transparent.

The feature importance plot is a helpful addition, as it aligns with clinical expectations that systolic and diastolic blood pressure are the strongest predictors. However, the figure could be described in a way that ties more directly to clinical practice. For example, explaining that BMI and proteinuria contribute modestly, while syphilis status and temperature contribute little, would help readers see which features add incremental value beyond standard hypertension screening.

Overall, the Results section demonstrates impressive effort in data analysis and visualization, but it could be improved by providing more nuance around the limitations of the modeling approach. Clearer acknowledgment of the gap between training performance and real-world validation would make the results more credible and prepare the ground for a balanced discussion.

Discussion

The discussion section does a good job of connecting the study results to the bigger picture of maternal health in low-resource settings. The authors clearly explain the challenges of poor data quality, lack of digital tools, and limited trained staff, while also pointing out that Tanzania’s investment in the UCS system is creating opportunities for digital health. It is also a strength that the authors show how the ML model identified predictors like BMI and protein in urine, which are often overlooked in routine care. This makes the case that ML can add value beyond just blood pressure readings.

However, the discussion tends to emphasize the positive findings more than the limitations. The model reached high accuracy but had very low precision, meaning it predicted a large number of women as “at risk” when they were not. This is an important issue because in a health system with limited resources, too many false positives could overwhelm staff and equipment. The authors should discuss this trade-off more directly and explain whether the model in its current form would really help health workers, or if it first needs further refinement.

The limitations section is well written but could be clearer. The authors explain that HDP was not directly recorded in the UCS and that blood pressure values were used instead. This is an important point, but it should also be noted that blood pressure in routine ANC can sometimes be recorded inaccurately, which could affect results. The lack of follow-up data is another key issue, because without clinical confirmation, it is difficult to know how reliable the predictions are outside the dataset.

The recommendations and key messages are helpful but read a bit broad. They could be made stronger by giving more concrete steps, such as improving digital training for health workers, making ANC data entry more standardized, or piloting the ML model in a few facilities before thinking about scaling up. These kinds of practical next steps would make the recommendations more useful to policymakers and health system leaders.

Overall, the discussion is clear and well-structured, but it needs to be more balanced. The authors should talk more openly about the limits of the model, especially the low precision, and give sharper, more realistic recommendations. This would make the paper stronger and show both the promise and the challenges of applying ML to maternal health in Tanzania.

Conclusion

This paper takes on an important problem and shows how machine learning could be used to predict high blood pressure in pregnancy using routine health data in Tanzania. The study makes good use of a large dataset, tests several models, and connects the work to real-world health challenges, which makes it both timely and relevant.

At the same time, the paper needs more clarity and balance. The results show very high accuracy but very low precision, which limits how useful the model would be in practice. The methods also need to be explained in more detail so readers can better understand and trust the findings. If the authors address these issues and provide more realistic recommendations for how the model could be used in low-resource settings, this study could make a strong contribution to maternal health research.

Reviewer #3:

Reviewer 3: Recommendation – major review

This paper presents a machine learning model designed to predict hypertensive disorders of pregnancy in Tanzania. Using a collection of 300,000 visit records, the authors tested several machine learning models, landing on an XGBoost model which provided promising output.

This paper is a nice example of a machine learning in a traditionally low-resource environment. This work was enabled by the recent implementation of a digital health record initiative of the Tanzanian government. The paper is well-written and generally clear. There are some shortcomings that should be addressable through edits to the paper:

1. Perhaps more importantly, key details are missing. Exactly which features were considered? Which were retained? What were the rates of missing data? How was missing data handled?

2. Toward the beginning of page 5, we are told that Pearson correlation coefficients were used to assess the strength of the relationship between features and outcomes. What was done with this information?

3. Under "model training", there is no mention of hyperparameter tuning? Was any such tuning attempted?

4. Under "descriptive results", there is a mention of "collapsing", which is not clear. Is this what is meant by the description in the methods of turning individual visits into a single longitudinal visit? This should be clarified.

5. Table 1 seems to indicate that some patients had 99 visits. That seems hard to imagine. Is this number correct? If so, it should be discussed.

6. Under model training results in Figure 3, no confidence intervals are given. Some notion of the variability of the models would be informative.

7. Table 2 discusses the performance of the "SGB" model, but this model is not mentioned or defined anywhere. Similarly, the "test" dataset is not described. Presumably, this is the 10% holdout: this should be clarified. No definition of "Macro" and "weighted" averages is given. Furthermore, the perfect score seems more than a bit surprising. Is there any explanation for this seemingly unlikely result?

8. Under "Feature Importance", the term "SHARP" results is used. This should read "SHAP".

9. The discussion is generally good, but we are left without any insight into the low precision of the models. Some discussion of potential causes and remedies would be helpful.

10. Some contextualization relative to prediction models for HDP in other contexts might help with interpretation of results. Examples include a recent paper in Scientific Reports (Chen & Ma, https://doi.org/10.1038/s41598-025-98416-y), a 2023 narrative review by Alkhodari, et al. (https://doi.org/10.1080/14779072.2023.2223978), and a 2018 systematic review by Ukah, et al. (https://doi.org/10.1016/j.preghy.2017.11.006).

11. When discussing implementation using tools such as Pandas, please provide version numbers.

[Note: HTML markup is below. Please do not edit.]

**Reviewers' Comments:**

Reviewer's Responses to Questions

**Comments to the Author**

1. Does this manuscript meet PLOS Digital Health’s publication criteria? Is the manuscript technically sound, and do the data support the conclusions? The manuscript must describe methodologically and ethically rigorous research with conclusions that are appropriately drawn based on the data presented.

Reviewer #1: No

Reviewer #2: Yes

Reviewer #3: Yes

**********

2. Has the statistical analysis been performed appropriately and rigorously?

Reviewer #1: No

Reviewer #2: Yes

Reviewer #3: Yes

**********

3. Have the authors made all data underlying the findings in their manuscript fully available (please refer to the Data Availability Statement at the start of the manuscript PDF file)?

The PLOS Data policy requires authors to make all data underlying the findings described in their manuscript fully available without restriction, with rare exception. The data should be provided as part of the manuscript or its supporting information, or deposited to a public repository. For example, in addition to summary statistics, the data points behind means, medians and variance measures should be available. If there are restrictions on publicly sharing data—e.g. participant privacy or use of data from a third party—those must be specified.

Reviewer #1: Yes

Reviewer #2: Yes

Reviewer #3: Yes

**********

4. Is the manuscript presented in an intelligible fashion and written in standard English?

PLOS Digital Health does not copyedit accepted manuscripts, so the language in submitted articles must be clear, correct, and unambiguous. Any typographical or grammatical errors should be corrected at revision, so please note any specific errors here.

Reviewer #1: Yes

Reviewer #2: Yes

Reviewer #3: Yes

**********

5. Review Comments to the Author

Please use the space provided to explain your answers to the questions above. You may also include additional comments for the author, including concerns about dual publication, research ethics, or publication ethics. (Please upload your review as an attachment if it exceeds 20,000 characters)

Reviewer #1: This paper uses ML models on a unique dataset to predict Hypertensive Disorders on Pregnant women in Tanzania, leveraging a databased developed by the Governement of Tanzania. The manuscript can be improved as follows:

Major comments:

- It is unclear why we should use machine learning to detect hypertension, when. the hypertensiuon is defined by a simple threshold of blood pressure measurements, and blood pressure is available to the model. The authors should explain better their reasoning for this.

- The study design has an important problem of data leak. The outcome is defined from the features of interests.

- The dataset is highly imbalance, which makes accuracy a suboptimal metric for evaluating performance.

- A confusion metrics is not provided

- The ROC curve used for calculating the AUC is not provided

- Overall, it is unclear the benefit of this approach, and how it contributes to the state-of-the-art. The authors should provide further information on why their approach is novel, and why.

Minor comments:

- Figure 1 has no x-axis label

- Page 3, when mentioning "In the context of maternal health, numerous studies have successfully developed and validated ML models", please cite these studies.

Reviewer #2: This manuscript tackles an important issue in maternal health by examining the use of machine learning to predict hypertensive disorders during pregnancy in Tanzania. The study is timely, utilizes a large dataset, and offers valuable insights into how digital health systems can aid early detection in low-resource settings. However, the paper would benefit from clearer descriptions of the methods, a more balanced interpretation of the results, and stronger, more practical recommendations for implementation. The main concern is the discrepancy between the high reported accuracy and the very low precision, which raises questions about the model’s real-world utility. Addressing this, along with increasing transparency in the analytic process and acknowledging data quality limitations, will significantly improve the work.

I have attached my full detailed review with section-by-section feedback for the authors.

Reviewer #3: This paper presents a machine learning model designed to predict

hypertensive disorders of pregnancy in Tanzania. Using a collection of

300,000 visit records, the authors tested several machine learning

models, landing on an XGBoost model which provided promising output.

This paper is a nice example of a machine learning in a traditionally

low-resource environment. This work was enabled by the recent

implementation of a digital health record initiative of the Tanzanian

government.

The paper is well-written and generally clear. There are some

shortcomings that should be addressable through edits to the paper:

1. Perhaps more importantly, key details are missing. Exactly which

features were considered? Which were retained? What were the rates of

missing data? How was missing data handled?

2. Toward the beginning of page 5, we are told that Pearson

correlation coefficients were used to assess the strength of the

relationship between features and outcomes. What was done with this

information?

3. Under "model training", there is no mention of hyperparameter

tuning? Was any such tuning attempted?

4. Under "descriptive results", there is a mention of "collapsing",

which is not clear. Is this what is meant by the description in the

methods of turning individual visits into a single longitudinal visit?

This should be clarified.

5. Table 1 seems to indicate that some patients had 99 visits. That

seems hard to imagine. Is this number correct? If so, it should be

discussed.

5. Under model training results in Figure 3, no confidence intervals

are given. Some notion of the variability of the models would be

informative.

6. Table 2 discusses the performance of the "SGB" model, but this

model is not mentioned or defined anywhere. Similarly, the "test"

dataset is not described. Presumably, this is the 10% holdout: this

should be clarified. No definition of "Macro" and "weighted" averages

is given. Furthermore, the perfect score seems more than a

bit surprising. Is there any explanation for this seemingly unlikely

result?

7. Under "Feature Importance", the term "SHARP" results is used. This

should read "SHAP".

8. The discussion is generally good, but we are left without any

insight into the low precision of the models. Some discussion of

potential causes and remedies would be helpful.

9. Some contextualization relative to prediction models for HDP in

other contexts might help with interpretation of results. Examples

include a recent paper in Scientific Reports (Chen & Ma,

https://doi.org/10.1038/s41598-025-98416-y), a 2023 narrative review

by Alkhodari, et al. (https://doi.org/10.1080/14779072.2023.2223978),

and a 2018 systematic review by Ukah, et

al. (https://doi.org/10.1016/j.preghy.2017.11.006).

include

9. When discussing implementation using tools such as Pandas, please

provide version numbers.

**********

6. PLOS authors have the option to publish the peer review history of their article (what does this mean?). If published, this will include your full peer review and any attached files.

**Do you want your identity to be public for this peer review?** If you choose “no”, your identity will remain anonymous but your review may still be made public.

For information about this choice, including consent withdrawal, please see our Privacy Policy.

Reviewer #1: No

Reviewer #2: **Yes:**Victor Ifechukwude Agboli

Reviewer #3: **Yes:**Harry Hochheiser

**********

[NOTE: If reviewer comments were submitted as an attachment file, they will be attached to this email and accessible via the submission site. Please log into your account, locate the manuscript record, and check for the action link "View Attachments". If this link does not appear, there are no attachment files.]

**Figure resubmission:**

While revising your submission, we strongly recommend that you use PLOS’s NAAS tool (https://ngplosjournals.pagemajik.ai/artanalysis) to test your figure files. NAAS can convert your figure files to the TIFF file type and meet basic requirements (such as print size, resolution), or provide you with a report on issues that do not meet our requirements and that NAAS cannot fix.

After uploading your figures to PLOS’s NAAS tool - https://ngplosjournals.pagemajik.ai/artanalysis, NAAS will process the files provided and display the results in the "Uploaded Files" section of the page as the processing is complete. If the uploaded figures meet our requirements (or NAAS is able to fix the files to meet our requirements), the figure will be marked as "fixed" above. If NAAS is unable to fix the files, a red "failed" label will appear above. When NAAS has confirmed that the figure files meet our requirements, please download the file via the download option, and include these NAAS processed figure files when submitting your revised manuscript.

**Reproducibility:**

To enhance the reproducibility of your results, we recommend that authors of applicable studies deposit laboratory protocols in protocols.io, where a protocol can be assigned its own identifier (DOI) such that it can be cited independently in the future. Additionally, PLOS ONE offers an option to publish peer-reviewed clinical study protocols. Read more information on sharing protocols at https://plos.org/protocols?utm_medium=editorial-email&utm_source=authorletters&utm_campaign=protocols

Attachment Submitted filename: Manuscript Review.docx

# Author comment: Author response to Decision Letter 1

**DOI:** 10.1371/journal.pdig.0001468.r002

20 Nov 2025

Attachment Submitted filename: PointByPointResponses.docx

# Peer review: Decision Letter 1

**DOI:** 10.1371/journal.pdig.0001468.r003

**Authors:** Dhiya Al-Jumeily OBE, Anna Khalemsky

11 Jan 2026

PDIG-D-25-00485R1

Harnessing Digital Health and Machine Learning for Early Detection of Hypertensive Disorders of Pregnancy: Insights from low-resource settings, Tanzania

PLOS Digital Health

Dear Dr. Lyatuu,

Thank you for submitting your manuscript to PLOS Digital Health. After careful consideration, we feel that it has merit but does not fully meet PLOS Digital Health's publication criteria as it currently stands. Therefore, we invite you to submit a revised version of the manuscript that addresses the points raised during the review process.

Please submit your revised manuscript by Mar 12 2026 11:59PM. If you will need more time than this to complete your revisions, please reply to this message or contact the journal office at digitalhealth@plos.org. When you're ready to submit your revision, log on to https://www.editorialmanager.com/pdig/ and select the 'Submissions Needing Revision' folder to locate your manuscript file.

Please include the following items when submitting your revised manuscript:

* A letter that responds to each point raised by the editor and reviewer(s). You should upload this letter as a separate file labeled 'Response to Reviewers'. This file does not need to include responses to any formatting updates and technical items listed in the 'Journal Requirements' section below.

* A marked-up copy of your manuscript that highlights changes made to the original version. You should upload this as a separate file labeled 'Revised Manuscript with Track Changes'.

* An unmarked version of your revised paper without tracked changes. You should upload this as a separate file labeled 'Manuscript'.

If you would like to make changes to your financial disclosure, competing interests statement, or data availability statement, please make these updates within the submission form at the time of resubmission. Guidelines for resubmitting your figure files are available below the reviewer comments at the end of this letter.

We look forward to receiving your revised manuscript.

Kind regards,

Anna Khalemsky, Ph.D.

Guest Editor

PLOS Digital Health

Anna Khalemsky

Guest Editor

PLOS Digital Health

Leo Anthony Celi

Editor-in-Chief

PLOS Digital Health

orcid.org/0000-0001-6712-6626

**Journal Requirements:**

1. Please provide a detailed online Financial Disclosure statement. This is published with the article. It must therefore be completed in full sentences and contain the exact wording you wish to be published.

a) State the initials, alongside each funding source, of each author to receive each grant. For example: “This work was supported by the National Institutes of Health (####### to AM; ###### to CJ) and the National Science Foundation (###### to AM).”

b) State what role the funders took in the study. If the funders had no role in your study, please state: “The funders had no role in study design, data collection and analysis, decision to publish, or preparation of the manuscript.”

For more information, please go to our submission guidelines:

https://journals.plos.org/digitalhealth/s/submission-guidelines#loc-financial-disclosure-statement

2. Please ensure that the funders and grant numbers match between the Financial Disclosure field and the Funding Information tab in your submission form. Note that the funders must be provided in the same order in both places as well.

3. Please update your online Competing Interests statement. If you have no competing interests to declare, please state: “The authors have declared that no competing interests exist.”

4. We note that you have indicated that there are restrictions to data sharing for this study. For studies involving human research participant data or other sensitive data, we encourage authors to share de-identified or anonymized data. However, when data cannot be publicly shared for ethical reasons, we allow authors to make their data sets available upon request. For information on unacceptable data access restrictions, please see http://journals.plos.org/digitalhealth/s/data-availability#loc-unacceptable-data-access-restrictions.

Before we proceed with your manuscript, please address the following prompts:

a) If there are ethical or legal restrictions on sharing a de-identified data set, please explain them in detail (e.g., data contain potentially identifying or sensitive patient information, data are owned by a third-party organization, etc.) and who has imposed them (e.g., a Research Ethics Committee or Institutional Review Board, etc.). Please also provide contact information for a data access committee, ethics committee, or other institutional body to which data requests may be sent.

b) If there are no restrictions, please upload the minimal anonymized data set necessary to replicate your study findings to a stable, public repository and provide us with the relevant URLs, DOIs, or accession numbers. Please see http://www.bmj.com/content/340/bmj.c181.long for guidelines on how to de-identify and prepare clinical data for publication. For a list of recommended repositories, please see https://journals.plos.org/digitalhealth/s/recommended-repositories. You also have the option of uploading the data as Supporting Information files, but we would recommend depositing data directly to a data repository if possible.

Please update your Data Availability statement in the submission form accordingly.

5. Please provide separate main figure files in .tif or .eps format only and ensure that all files are under our size limit of 10MB. You may leave the embedded figures in the manuscript.

For more information about how to convert your figure files please see our guidelines: https://journals.plos.org/digitalhealth/s/figures

6. If the reviewer comments include a recommendation to cite specific previously published works, please review and evaluate these publications to determine whether they are relevant and should be cited. There is no requirement to cite these works unless the editor has indicated otherwise.

**Additional Editor Comments (if provided):**

Harnessing Digital Health and Machine Learning for Early Detection of Hypertensive Disorders of Pregnancy

The three reviews converge on the view that this manuscript addresses an important and timely problem and demonstrates substantial technical effort, improved transparency, and clearer positioning compared to the previous version. Reviewer 2 considers the work close to publishable with minor refinements, particularly around tempering claims, clarifying operational implications of low precision, and contextualizing near-perfect training performance. In contrast, Reviewers 4 and 5 raise significant concerns regarding the use of proxy outcome definitions derived from blood pressure thresholds, the circularity between predictors and labels, severe precision limitations, loss of temporal information, missing data handling, and overstatement of clinical readiness. Collectively, these issues do not invalidate the study’s value as a large-scale, retrospective, proof-of-concept analysis using routine ANC data, but they do require substantial reframing, additional analyses, and stronger methodological transparency. I therefore recommend major revision according to reviewers comments. Addressing these points would significantly strengthen the manuscript’s credibility and alignment with implementation-oriented digital health research.

Reviewer 2 – minor revision

The revised manuscript is much improved and addresses the main concerns from the previous review. The abstract, methods, and results are now better, especially regarding how the dataset was built, how class imbalance was handled, how independent validation was done, and how the model is positioned as a high-sensitivity screening tool rather than a diagnostic system. The authors have improved the methods by clearly describing preprocessing, feature selection, cross-validation, and performance evaluation. The discussion now more openly addresses the impact of low precision in settings with limited resources and offers practical implementation recommendations. A few minor issues remain, such as the need to temper further the interpretation of near-perfect training performance and to more clearly explain the operational burden of false positives, but these do not detract from the overall value of the work.

Abstract

What was done well:

The abstract is much better now. The authors clearly explain the trade-off between sensitivity and precision and describe the model as a high-sensitivity screening tool rather than a diagnostic system. They have clarified the dataset size by distinguishing total ANC visits, collapsed client records, the balanced training subset, and the independent validation dataset. This addresses my earlier concerns about transparency and generalizability. The tone is now more restrained and better suited to a proof-of-concept study.

What remains to be done:

The abstract still focuses too much on accuracy and AUC, which could mislead non-technical readers, given the very low precision. Although the trade-off is mentioned, adding a short phrase that quantifies the operational impact, like the number of false positives per true positive, would make the summary more realistic.

Introduction

What was done well:

The introduction is clearer and better organized. The authors now state the research gap: few ML studies use routine government ANC data at a national level in low-resource settings. Background on ANC reporting and UCS is shortened and moved, and the study objectives are introduced earlier. This improves narrative flow and highlights novelty.

What remains to be done:

The introduction would be improved by a clearer comparison with previous ML-based HDP studies, especially regarding performance metrics such as precision. Right now, the novelty is mostly about the data source and context, not about how the methods differ from existing models. A comparison is needed here.

Methods

What was done well:

This section is the most improved. The authors now give detailed descriptions of data preprocessing, visit collapsing, handling missing data, feature selection, class balancing, cross-validation, hyperparameter tuning, and independent validation. They clearly explain why they collapsed the longitudinal data, based on real-world needs. They also openly discuss the potential for data leakage when using BP-based outcome definitions. The modeling process is now reproducible and defensible.

What remains to be done:

Even with improved transparency, the outcome definition remains limited by using BP thresholds, which are also the top predictors. The authors mention this, but they should make it clearer that the model’s performance does not show true risk prediction. Instead, it should be seen as improved screening in settings with noisy measurements. This point is still unclear. In addition, for the correlation analysis to detect collinearity, it assumes that the continuous variables have a linear relationship. What if this assumption is wrong? The authors need to state how they checked this.

Results

What was done well:

The Results section is now more honest and informative. The authors present confusion matrices and ROC curves, assess calibration, and carefully analyze cases where results differ. They show and discuss the big drop in precision during real-world validation. Feature importance is also explained in clinical terms. This addresses earlier concerns about overfitting and selective reporting.

What remains to be done:

The almost-perfect cross-validation results on the balanced training data are still presented without sufficient caution. Overfitting is mentioned, but the manuscript should clearly say that these results do not prove the model is better. Instead, they are likely due to under-sampling and how the labels were created.

Discussion and Conclusion

What was done well:

The discussion is more balanced and improved. The authors now address the effects of low precision in resource-limited systems and give step-by-step recommendations for piloting, improving data quality, training, and policy integration. The conclusion presents the work as a proof-of-concept needing further validation.

What remains to be done:

The discussion still sometimes overstates the clinical benefits while failing to fully account for the additional burden on the system. Adding a clear statement that using the current model could greatly increase clinical workload would make the paper more credible and better aligned with implementation science.

I strongly believe addressing these concerns will improve the paper.

Reviewer 4 – major revision

This is an important and well-motivated study. However, the use of a proxy HDP label, low precision, and lack of temporal modeling limit the reliability of the model’s predictions. The manuscript would benefit from more rigorous evaluation, expanded discussion of implementation challenges, and clearer framing of its current role as exploratory rather than deployment-ready.

The authors present a retrospective study applying machine learning (ML) to antenatal care (ANC) data collected from Tanzania’s Unified Community System (UCS) to predict hypertensive disorders of pregnancy (HDP). They evaluate several classifiers, selecting XGBoost for deployment, reporting an AUC of 0.95 and 90.95% accuracy on unseen data (n >120,000). The goal is to demonstrate how digital health data can support early detection of HDP in lowresource environments. Both prior reviewers agree that the paper demonstrates relevance, ambition, and technical effort, but also exhibits substantial limitations in clinical labeling, model evaluation, temporal data usage, and real-world implementation claims. This final review consolidates those points, offering a full technical evaluation.

Major Issues

1. Use of Proxy Outcome Definition (HDP) Without Clinical Validation. The outcome label for HDP is derived solely from systolic and diastolic blood pressure thresholds (≥140/90 mmHg), without confirmation from diagnosis fields, medication data, or clinician annotations. This label construction is acknowledged as a proxy but remains fundamentally weak for building clinically trustworthy prediction models. Impact: This creates a risk of label noise, undermines model evaluation, and raises concerns about internal validity. Recommendation: While data limitations are acknowledged, the paper must reframe its scope from “early detection of HDP” to “classification of blood pressure risk states,” and clearly state the limits of proxy labeling in both methods and conclusions.

2. Severe Precision Deficit (14%) With Limited Operational Mitigation Both reviews noted that the model exhibits a 14% precision—indicating that most positive predictions are false positives. The authors attempt to justify this through high recall and potential for early warning, but there is no quantitative or operational analysis of the downstream clinical impact of such a high false positive burden in resourceconstrained settings. Impact: This challenges the model’s practical deployability and user trust. Recommendation: Authors must include a risk-benefit discussion, propose specific triaging protocols, or demonstrate via simulation how false positives would be handled without overwhelming staff or infrastructure.

3. Flattening of Temporal Data is a Structural Limitation. The decision to aggregate longitudinal ANC visit data into static per-patient feature vectors eliminates critical gestational progression patterns, which are central to HDP pathophysiology. This limitation is acknowledged but not sufficiently addressed. Impact: This structurally limits predictive accuracy and masks important trends such as rising BP trajectories or changes in proteinuria over time. Recommendation: Authors should either experiment with temporally-aware models or provide structured temporal feature engineering (e.g., BP slopes, deltas), and fully articulate the implications of the current model’s static nature.

4. Lack of External or Prospective Validation While the model is tested on a large test cohort (n = 120,232), no prospective, geographic, or temporal external validation is performed. Furthermore, predictions are not linked to actual delivery or maternal outcomes. Impact: Limits generalizability and weakens claims about readiness for deployment or policy use. Recommendation: The manuscript should temper claims regarding deployment and emphasize the need for a formal prospective validation study. A pilot phase or follow-up outcome analysis would significantly strengthen future versions.

5. Omission of Missing Data and Data Quality Protocols There is no description of missing data handling, which is critical in LMIC digital health datasets. Features such as glucose, protein in urine, and syphilis status are often missing or inconsistently recorded. Impact: Compromises the transparency, reproducibility, and robustness of the model. Recommendation: Authors must report missingness rates per feature, describe imputation or exclusion methods, and assess model sensitivity to data completeness.

Minor Issues

• Model threshold selection and calibration are not discussed. Threshold optimization (e.g., Youden Index, F1-maximization) under class imbalance should be detailed.

• Feature definitions are ambiguous in several places (e.g., “ANC risk flag,” “partner HIV status”); authors should provide a data dictionary or glossary.

• Use of ROC curves is insufficient under high class imbalance; precision-recall curves should be included.

• SHAP plots are informative but need clearer axis labels and clinical interpretation guidance.

• Terminological ambiguity between “HDP” and “gestational hypertension” must be resolved; either clarify or limit the scope of what the model is predicting.

Figures and Tables

Figures are well constructed but need improvements in labeling. A confusion matrix and precision-recall curve should be included. Tables summarizing missing data rates and class distribution across training, validation, and test sets are missing and should be provided. Methods Structurally adequate but lacking in key details:

• No account of data cleaning or outlier handling

• No missing data methodology

• No description of feature engineering or variable encoding

• Sampling strategy for balancing classes is not defined Study Design Appropriate as a retrospective feasibility study. However, the title and framing imply implementation readiness, which the current evidence does not support. A prospective or hybrid study design would be a more suitable next step.

Any Experiments Missing?

• External or temporal validation experiment

• Sensitivity analysis of label definitions • Subgroup analysis by gestational age or risk tier

• Threshold tuning experiment Statistics Baseline metrics are acceptable, but calibration (e.g., Brier score, reliability diagrams) is not assessed. Confidence intervals for key metrics (AUC, precision, recall) are missing.

Results and Data: Support Conclusions?

Partially. The technical performance is adequate, but the clinical conclusions and implementation claims outpace the underlying evidence.

All Data Accessible? No data or code sharing statement is present. Authors must clarify availability of de-identified data and model artifacts for reproducibility.

References

Sufficient for background and methodology. Authors may consider referencing clinical HDP prediction tools (e.g., fullPIERS, PREP-S) and ethical implications of ML in maternal health. Title

Accurate but overstated. Given the limitations in clinical outcome validation, a more accurate title would replace “Early Detection of Hypertensive Disorders of Pregnancy” with “Machine Learning Risk Classification of Elevated Blood Pressure in ANC Records.”

Reviewer 5 – reject

Recommendations:

1. The study uses blood pressure measurements to both define HDP and as the primary predictor variables, creating inherent circular reasoning. This undermines the core premise that machine learning adds value beyond simple threshold-based screening. The authors should either reframe this as a digital infrastructure feasibility study, obtain clinically confirmed HDP diagnoses for proper validation, or demonstrate true temporal prediction by using early visit data to predict HDP at later visits.

2. The title and abstract emphasize early detection, but the study design only demonstrates concurrent classification using current blood pressure readings, not prediction before clinical onset. The discordant case analysis shows that false positives have blood pressure just below diagnostic thresholds (136.5 vs 140 mmHg), which represents borderline detection rather than early warning. Authors should either demonstrate genuine early prediction using data from first trimester visits to predict later HDP, or reframe their contribution as "enhanced screening support" rather than early detection.

3. Excluding 73% of records (136,864 of 187,438) due to missing data represents a critical threat to generalizability that is inadequately discussed. Authors must provide a comparison table showing whether excluded records differ systematically from included records, analyze missingness patterns (MCAR vs MAR vs MNAR), and justify why complete-case analysis was chosen over imputation methods. This selection bias could severely limit real-world applicability and must be transparently reported with implications for model deployment.

4. While the authors justify prioritizing sensitivity, the 14% precision means 86% of flagged women do not actually have HDP, and the clinical workflow implications are unexplored. The manuscript needs to include baseline HDP prevalence in Tanzanian populations, describe the clinical pathway for managing flagged cases, estimate resource costs of investigating 12,603 alerts to find 1,725 true cases, and compare performance against existing standard-of-care detection rates. A sensitivity analysis showing model performance at different probability thresholds would also strengthen the clinical applicability discussion.

5. The decision to collapse multiple ANC visits into single records using different aggregation rules (means for BP, maximum for gestational age, most recent for categorical variables) appears arbitrary and destroys potentially valuable temporal patterns. Authors should provide explicit justification for each aggregation method, consider time-series approaches or visit-specific models as alternatives, and at minimum conduct sensitivity analyses using different aggregation strategies. This is particularly important since BP trends over pregnancy are clinically meaningful for HDP risk assessment.

6. The validation dataset (April-May 2024) comes from the same population, facilities, and data collection system as the training data (pre-April 2024), which tests temporal stability rather than true generalizability. Authors should add geographic or facility-level cross-validation, report stratified performance by region and patient demographics, and explicitly discuss how the model would perform in facilities without UCS infrastructure. Without this, claims about scalability and broader implementation remain unsubstantiated.

7. Given that BP dominates the model (SHAP values +7.94 and +7.62) and the outcome is defined by BP thresholds, the manuscript must explicitly demonstrate what additional value machine learning provides over simple clinical protocols. Authors should conduct an ablation study showing model performance when BP features are excluded entirely, compare detection rates against current Tanzanian ANC guidelines, and provide concrete examples of cases where the integration of BMI, proteinuria, and other factors identified at-risk women who would have been missed by BP screening alone. This is essential to justify the added complexity and resource investment of ML deployment.

[Note: HTML markup is below. Please do not edit.]

**Reviewers' Comments:**

Reviewer's Responses to Questions

**Comments to the Author**

1. If the authors have adequately addressed your comments raised in a previous round of review and you feel that this manuscript is now acceptable for publication, you may indicate that here to bypass the “Comments to the Author” section, enter your conflict of interest statement in the “Confidential to Editor” section, and submit your "Accept" recommendation.

Reviewer #2: All comments have been addressed

Reviewer #4: (No Response)

Reviewer #5: All comments have been addressed

**********

2. Does this manuscript meet PLOS Digital Health’s publication criteria? Is the manuscript technically sound, and do the data support the conclusions? The manuscript must describe methodologically and ethically rigorous research with conclusions that are appropriately drawn based on the data presented.

Reviewer #2: Yes

Reviewer #4: Partly

Reviewer #5: Partly

**********

3. Has the statistical analysis been performed appropriately and rigorously?

Reviewer #2: Yes

Reviewer #4: No

Reviewer #5: Yes

**********

4. Have the authors made all data underlying the findings in their manuscript fully available (please refer to the Data Availability Statement at the start of the manuscript PDF file)?

The PLOS Data policy requires authors to make all data underlying the findings described in their manuscript fully available without restriction, with rare exception. The data should be provided as part of the manuscript or its supporting information, or deposited to a public repository. For example, in addition to summary statistics, the data points behind means, medians and variance measures should be available. If there are restrictions on publicly sharing data—e.g. participant privacy or use of data from a third party—those must be specified.

Reviewer #2: Yes

Reviewer #4: Yes

Reviewer #5: No

**********

5. Is the manuscript presented in an intelligible fashion and written in standard English?

PLOS Digital Health does not copyedit accepted manuscripts, so the language in submitted articles must be clear, correct, and unambiguous. Any typographical or grammatical errors should be corrected at revision, so please note any specific errors here.

Reviewer #2: Yes

Reviewer #4: Yes

Reviewer #5: (No Response)

**********

6. Review Comments to the Author

Please use the space provided to explain your answers to the questions above. You may also include additional comments for the author, including concerns about dual publication, research ethics, or publication ethics. (Please upload your review as an attachment if it exceeds 20,000 characters)

Reviewer #2: The revised manuscript is much improved and addresses the main concerns from the previous review. The abstract, methods, and results are now better, especially regarding how the dataset was built, how class imbalance was handled, how independent validation was done, and how the model is positioned as a high-sensitivity screening tool rather than a diagnostic system. The authors have improved the methods by clearly describing preprocessing, feature selection, cross-validation, and performance evaluation. The discussion now more openly addresses the impact of low precision in settings with limited resources and offers practical implementation recommendations. A few minor issues remain, such as the need to temper further the interpretation of near-perfect training performance and to more clearly explain the operational burden of false positives, but these do not detract from the overall value of the work.

Please see my attached comments for detailed, section-by-section suggestions.

Reviewer #4: This is an important and well-motivated study. However, the use of a proxy HDP label, low precision, and lack of temporal modeling limit the reliability of the model’s predictions. The manuscript would benefit from more rigorous evaluation, expanded discussion of implementation challenges, and clearer framing of its current role as exploratory rather than deployment-ready.

Reviewer #5: Recommendations:

1. The study uses blood pressure measurements to both define HDP and as the primary predictor variables, creating inherent circular reasoning. This undermines the core premise that machine learning adds value beyond simple threshold-based screening. The authors should either reframe this as a digital infrastructure feasibility study, obtain clinically confirmed HDP diagnoses for proper validation, or demonstrate true temporal prediction by using early visit data to predict HDP at later visits.

2. The title and abstract emphasize early detection, but the study design only demonstrates concurrent classification using current blood pressure readings, not prediction before clinical onset. The discordant case analysis shows that false positives have blood pressure just below diagnostic thresholds (136.5 vs 140 mmHg), which represents borderline detection rather than early warning. Authors should either demonstrate genuine early prediction using data from first trimester visits to predict later HDP, or reframe their contribution as "enhanced screening support" rather than early detection.

3. Excluding 73% of records (136,864 of 187,438) due to missing data represents a critical threat to generalizability that is inadequately discussed. Authors must provide a comparison table showing whether excluded records differ systematically from included records, analyze missingness patterns (MCAR vs MAR vs MNAR), and justify why complete-case analysis was chosen over imputation methods. This selection bias could severely limit real-world applicability and must be transparently reported with implications for model deployment.

4. While the authors justify prioritizing sensitivity, the 14% precision means 86% of flagged women do not actually have HDP, and the clinical workflow implications are unexplored. The manuscript needs to include baseline HDP prevalence in Tanzanian populations, describe the clinical pathway for managing flagged cases, estimate resource costs of investigating 12,603 alerts to find 1,725 true cases, and compare performance against existing standard-of-care detection rates. A sensitivity analysis showing model performance at different probability thresholds would also strengthen the clinical applicability discussion.

5. The decision to collapse multiple ANC visits into single records using different aggregation rules (means for BP, maximum for gestational age, most recent for categorical variables) appears arbitrary and destroys potentially valuable temporal patterns. Authors should provide explicit justification for each aggregation method, consider time-series approaches or visit-specific models as alternatives, and at minimum conduct sensitivity analyses using different aggregation strategies. This is particularly important since BP trends over pregnancy are clinically meaningful for HDP risk assessment.

6. The validation dataset (April-May 2024) comes from the same population, facilities, and data collection system as the training data (pre-April 2024), which tests temporal stability rather than true generalizability. Authors should add geographic or facility-level cross-validation, report stratified performance by region and patient demographics, and explicitly discuss how the model would perform in facilities without UCS infrastructure. Without this, claims about scalability and broader implementation remain unsubstantiated.

7. Given that BP dominates the model (SHAP values +7.94 and +7.62) and the outcome is defined by BP thresholds, the manuscript must explicitly demonstrate what additional value machine learning provides over simple clinical protocols. Authors should conduct an ablation study showing model performance when BP features are excluded entirely, compare detection rates against current Tanzanian ANC guidelines, and provide concrete examples of cases where the integration of BMI, proteinuria, and other factors identified at-risk women who would have been missed by BP screening alone. This is essential to justify the added complexity and resource investment of ML deployment.

**********

7. PLOS authors have the option to publish the peer review history of their article (what does this mean?). If published, this will include your full peer review and any attached files.

**Do you want your identity to be public for this peer review?** If you choose “no”, your identity will remain anonymous but your review may still be made public.

For information about this choice, including consent withdrawal, please see our Privacy Policy.

Reviewer #2: **Yes:**Victor Ifechukwude Agboli

Reviewer #4: No

Reviewer #5: No

**********

[NOTE: If reviewer comments were submitted as an attachment file, they will be attached to this email and accessible via the submission site. Please log into your account, locate the manuscript record, and check for the action link "View Attachments". If this link does not appear, there are no attachment files.]

**Figure resubmission:**

While revising your submission, we strongly recommend that you use PLOS’s NAAS tool (https://ngplosjournals.pagemajik.ai/artanalysis) to test your figure files. NAAS can convert your figure files to the TIFF file type and meet basic requirements (such as print size, resolution), or provide you with a report on issues that do not meet our requirements and that NAAS cannot fix.

After uploading your figures to PLOS’s NAAS tool - https://ngplosjournals.pagemajik.ai/artanalysis, NAAS will process the files provided and display the results in the "Uploaded Files" section of the page as the processing is complete. If the uploaded figures meet our requirements (or NAAS is able to fix the files to meet our requirements), the figure will be marked as "fixed" above. If NAAS is unable to fix the files, a red "failed" label will appear above. When NAAS has confirmed that the figure files meet our requirements, please download the file via the download option, and include these NAAS processed figure files when submitting your revised manuscript.

**Reproducibility:**

To enhance the reproducibility of your results, we recommend that authors of applicable studies deposit laboratory protocols in protocols.io, where a protocol can be assigned its own identifier (DOI) such that it can be cited independently in the future. Additionally, PLOS ONE offers an option to publish peer-reviewed clinical study protocols. Read more information on sharing protocols at https://plos.org/protocols?utm_medium=editorial-email&utm_source=authorletters&utm_campaign=protocols

Attachment Submitted filename: Manuscript_Review_declet_1.docx

Attachment Submitted filename: PDIG-D-25-00485R1_reviewer_JULIAN_BORGES.pdf

# Author comment: Author response to Decision Letter 2

**DOI:** 10.1371/journal.pdig.0001468.r004

23 Mar 2026

Attachment Submitted filename: ManuscriptPointByPointResponses.docx

# Peer review: Decision Letter 2

**DOI:** 10.1371/journal.pdig.0001468.r005

**Authors:** Dhiya Al-Jumeily OBE, Anna Khalemsky

13 May 2026

Machine Learning for Risk Stratification of Hypertensive Disorders of Pregnancy: Enhancing Clinical Efficiency in low-resource Antenatal Care in Tanzania

PDIG-D-25-00485R2

Dear Dr. Lyatuu,

We are pleased to inform you that your manuscript 'Machine Learning for Risk Stratification of Hypertensive Disorders of Pregnancy: Enhancing Clinical Efficiency in low-resource Antenatal Care in Tanzania' has been provisionally accepted for publication in PLOS Digital Health.

Before your manuscript can be formally accepted you will need to complete some formatting changes, which you will receive in a follow-up email from a member of our team.

Please note that your manuscript will not be scheduled for publication until you have made the required changes, so a swift response is appreciated.

IMPORTANT: The editorial review process is now complete. PLOS will only permit corrections to spelling, formatting or significant scientific errors from this point onwards. Requests for major changes, or any which affect the scientific understanding of your work, will cause delays to the publication date of your manuscript.

If your institution or institutions have a press office, please notify them about your upcoming paper to help maximize its impact. If they'll be preparing press materials, please inform our press team as soon as possible -- no later than 48 hours after receiving the formal acceptance. Your manuscript will remain under strict press embargo until 2 pm Eastern Time on the date of publication. For more information, please contact digitalhealth@plos.org.

Thank you again for supporting Open Access publishing; we are looking forward to publishing your work in PLOS Digital Health.

Best regards,

Anna Khalemsky, Ph.D.

Guest Editor

PLOS Digital Health

***********************************************************

**Additional Editor Comments (if provided):**

Recommendation letter

Subject: Decision on Manuscript PDIG-D-25-00485: Harnessing Digital Health and Machine Learning for Early Detection of Hypertensive Disorders of Pregnancy: Insights from Low-Resource Settings, Tanzania

Dear Isaac Lyatuu,

Thank you for submitting your revised manuscript and for addressing the reviewers’ comments. After careful consideration of the revisions and the reviewers’ evaluations, I regret to inform you that the manuscript cannot be accepted for publication in its current form.

While improvements have been made, the remaining concerns are substantial and would require major reconceptualization rather than further revision.

Thank you for considering our journal for your work.

Sincerely,

Anna Khalemsky, PhD

Reviewer 2: Recommendation – minor revision

Abstract

What was done well:

The abstract is much better now. The authors clearly explain the trade-off between sensitivity and precision and describe the model as a high-sensitivity screening tool rather than a diagnostic system. They have clarified the dataset size by distinguishing total ANC visits, collapsed client records, the balanced training subset, and the independent validation dataset. This addresses my earlier concerns about transparency and generalizability. The tone is now more restrained and better suited to a proof-of-concept study.

What remains to be done:

The abstract still focuses too much on accuracy and AUC, which could mislead non-technical readers, given the very low precision. Although the trade-off is mentioned, adding a short phrase that quantifies the operational impact, like the number of false positives per true positive, would make the summary more realistic.

Introduction

What was done well:

The introduction is clearer and better organized. The authors now state the research gap: few ML studies use routine government ANC data at a national level in low-resource settings. Background on ANC reporting and UCS is shortened and moved, and the study objectives are introduced earlier. This improves narrative flow and highlights novelty.

What remains to be done:

The introduction would be improved by a clearer comparison with previous ML-based HDP studies, especially regarding performance metrics such as precision. Right now, the novelty is mostly about the data source and context, not about how the methods differ from existing models. A comparison is needed here.

Methods

What was done well:

This section is the most improved. The authors now give detailed descriptions of data preprocessing, visit collapsing, handling missing data, feature selection, class balancing, cross-validation, hyperparameter tuning, and independent validation. They clearly explain why they collapsed the longitudinal data, based on real-world needs. They also openly discuss the potential for data leakage when using BP-based outcome definitions. The modeling process is now reproducible and defensible.

What remains to be done:

Even with improved transparency, the outcome definition remains limited by using BP thresholds, which are also the top predictors. The authors mention this, but they should make it clearer that the model’s performance does not show true risk prediction. Instead, it should be seen as improved screening in settings with noisy measurements. This point is still unclear. In addition, for the correlation analysis to detect collinearity, it assumes that the continuous variables have a linear relationship. What if this assumption is wrong? The authors need to state how they checked this.

Results

What was done well:

The Results section is now more honest and informative. The authors present confusion matrices and ROC curves, assess calibration, and carefully analyze cases where results differ. They show and discuss the big drop in precision during real-world validation. Feature importance is also explained in clinical terms. This addresses earlier concerns about overfitting and selective reporting.

What remains to be done:

The almost-perfect cross-validation results on the balanced training data are still presented without sufficient caution. Overfitting is mentioned, but the manuscript should clearly say that these results do not prove the model is better. Instead, they are likely due to under-sampling and how the labels were created.

Discussion and Conclusion

What was done well:

The discussion is more balanced and improved. The authors now address the effects of low precision in resource-limited systems and give step-by-step recommendations for piloting, improving data quality, training, and policy integration. The conclusion presents the work as a proof-of-concept needing further validation.

What remains to be done:

The discussion still sometimes overstates the clinical benefits while failing to fully account for the additional burden on the system. Adding a clear statement that using the current model could greatly increase clinical workload would make the paper more credible and better aligned with implementation science.

I strongly believe addressing these concerns will improve the paper.

Thank you.

Reviewer 4: Recommendation – major revision

Summary

The authors present a retrospective study applying machine learning (ML) to antenatal care (ANC) data collected from Tanzania’s Unified Community System (UCS) to predict hypertensive disorders of pregnancy (HDP). They evaluate several classifiers, selecting XGBoost for deployment, reporting an AUC of 0.95 and 90.95% accuracy on unseen data (n >120,000). The goal is to demonstrate how digital health data can support early detection of HDP in low resource environments.

Both prior reviewers agree that the paper demonstrates relevance, ambition, and technical effort, but also exhibits substantial limitations in clinical labeling, model evaluation, temporal data usage, and real-world implementation claims. This final review consolidates those points, offering a full technical evaluation.

Major Issues

1. Use of Proxy Outcome Definition (HDP) Without Clinical Validation

The outcome label for HDP is derived solely from systolic and diastolic blood pressure thresholds (≥140/90 mmHg), without confirmation from diagnosis fields, medication data, or clinician annotations. This label construction is acknowledged as a proxy but remains fundamentally weak for building clinically trustworthy prediction models.

Impact: This creates a risk of label noise, undermines model evaluation, and raises concerns about internal validity.

Recommendation: While data limitations are acknowledged, the paper must reframe its scope from “early detection of HDP” to “classification of blood pressure risk states,” and clearly state the limits of proxy labeling in both methods and conclusions.

2. Severe Precision Deficit (14%) With Limited Operational Mitigation

Both reviews noted that the model exhibits a 14% precision—indicating that most positive predictions are false positives. The authors attempt to justify this through high recall and potential for early warning, but there is no quantitative or operational analysis of the downstream clinical impact of such a high false positive burden in resource constrained settings.

Impact: This challenges the model’s practical deployability and user trust.

Recommendation: Authors must include a risk-benefit discussion, propose specific triaging protocols, or demonstrate via simulation how false positives would be handled without overwhelming staff or infrastructure.

3. Flattening of Temporal Data is a Structural Limitation

The decision to aggregate longitudinal ANC visit data into static per-patient feature vectors eliminates critical gestational progression patterns, which are central to HDP pathophysiology. This limitation is acknowledged but not sufficiently addressed.

Impact: This structurally limits predictive accuracy and masks important trends such as rising BP trajectories or changes in proteinuria over time.

Recommendation: Authors should either experiment with temporally-aware models or provide structured temporal feature engineering (e.g., BP slopes, deltas), and fully articulate the implications of the current model’s static nature.

4. Lack of External or Prospective Validation

While the model is tested on a large test cohort (n = 120,232), no prospective, geographic, or temporal external validation is performed. Furthermore, predictions are not linked to actual delivery or maternal outcomes.

Impact: Limits generalizability and weakens claims about readiness for deployment or policy use.

Recommendation: The manuscript should temper claims regarding deployment and emphasize the need for a formal prospective validation study. A pilot phase or follow-up outcome analysis would significantly strengthen future versions.

5. Omission of Missing Data and Data Quality Protocols

There is no description of missing data handling, which is critical in LMIC digital health datasets. Features such as glucose, protein in urine, and syphilis status are often missing or inconsistently recorded.

Impact: Compromises the transparency, reproducibility, and robustness of the model.

Recommendation: Authors must report missingness rates per feature, describe imputation or exclusion methods, and assess model sensitivity to data completeness.

Minor Issues

1. Model threshold selection and calibration are not discussed. Threshold optimization (e.g., Youden Index, F1-maximization) under class imbalance should be detailed.

2. Feature definitions are ambiguous in several places (e.g., “ANC risk flag,” “partner HIV status”); authors should provide a data dictionary or glossary.

3. Use of ROC curves is insufficient under high class imbalance; precision-recall curves should be included.

4. SHAP plots are informative but need clearer axis labels and clinical interpretation guidance.

5. Terminological ambiguity between “HDP” and “gestational hypertension” must be resolved; either clarify or limit the scope of what the model is predicting.

Figures and Tables

Figures are well constructed but need improvements in labeling. A confusion matrix and precision-recall curve should be included. Tables summarizing missing data rates and class distribution across training, validation, and test sets are missing and should be provided.

Methods

1. Structurally adequate but lacking in key details:

2. No account of data cleaning or outlier handling

3. No missing data methodology

4. No description of feature engineering or variable encoding

5. Sampling strategy for balancing classes is not defined

Study Design

Appropriate as a retrospective feasibility study. However, the title and framing imply implementation readiness, which the current evidence does not support. A prospective or hybrid study design would be a more suitable next step.

1. Any Experiments Missing?

2. External or temporal validation experiment

3. Sensitivity analysis of label definitions

4. Subgroup analysis by gestational age or risk tier

5. Threshold tuning experiment

Statistics. Baseline metrics are acceptable, but calibration (e.g., Brier score, reliability diagrams) is not assessed. Confidence intervals for key metrics (AUC, precision, recall) are missing.

Results and Data: Support Conclusions? Partially. The technical performance is adequate, but the clinical conclusions and implementation claims outpace the underlying evidence.

All Data Accessible? No data or code sharing statement is present. Authors must clarify availability of de-identified data and model artifacts for reproducibility.

References. Sufficient for background and methodology. Authors may consider referencing clinical HDP prediction tools (e.g., fullPIERS, PREP-S) and ethical implications of ML in maternal health.

Title. Accurate but overstated. Given the limitations in clinical outcome validation, a more accurate title would replace “Early Detection of Hypertensive Disorders of Pregnancy” with “Machine Learning Risk Classification of Elevated Blood Pressure in ANC Records

Reviewer 5: Recommendation – reject

Recommendations:

1. The study uses blood pressure measurements to both define HDP and as the primary predictor variables, creating inherent circular reasoning. This undermines the core premise that machine learning adds value beyond simple threshold-based screening. The authors should either reframe this as a digital infrastructure feasibility study, obtain clinically confirmed HDP diagnoses for proper validation, or demonstrate true temporal prediction by using early visit data to predict HDP at later visits.

2. The title and abstract emphasize early detection, but the study design only demonstrates concurrent classification using current blood pressure readings, not prediction before clinical onset. The discordant case analysis shows that false positives have blood pressure just below diagnostic thresholds (136.5 vs 140 mmHg), which represents borderline detection rather than early warning. Authors should either demonstrate genuine early prediction using data from first trimester visits to predict later HDP, or reframe their contribution as "enhanced screening support" rather than early detection.

3. Excluding 73% of records (136,864 of 187,438) due to missing data represents a critical threat to generalizability that is inadequately discussed. Authors must provide a comparison table showing whether excluded records differ systematically from included records, analyze missingness patterns (MCAR vs MAR vs MNAR), and justify why complete-case analysis was chosen over imputation methods. This selection bias could severely limit real-world applicability and must be transparently reported with implications for model deployment.

4. While the authors justify prioritizing sensitivity, the 14% precision means 86% of flagged women do not actually have HDP, and the clinical workflow implications are unexplored. The manuscript needs to include baseline HDP prevalence in Tanzanian populations, describe the clinical pathway for managing flagged cases, estimate resource costs of investigating 12,603 alerts to find 1,725 true cases, and compare performance against existing standard-of-care detection rates. A sensitivity analysis showing model performance at different probability thresholds would also strengthen the clinical applicability discussion.

5. The decision to collapse multiple ANC visits into single records using different aggregation rules (means for BP, maximum for gestational age, most recent for categorical variables) appears arbitrary and destroys potentially valuable temporal patterns. Authors should provide explicit justification for each aggregation method, consider time-series approaches or visit-specific models as alternatives, and at minimum conduct sensitivity analyses using different aggregation strategies. This is particularly important since BP trends over pregnancy are clinically meaningful for HDP risk assessment.

6. The validation dataset (April-May 2024) comes from the same population, facilities, and data collection system as the training data (pre-April 2024), which tests temporal stability rather than true generalizability. Authors should add geographic or facility-level cross-validation, report stratified performance by region and patient demographics, and explicitly discuss how the model would perform in facilities without UCS infrastructure. Without this, claims about scalability and broader implementation remain unsubstantiated.

7. Given that BP dominates the model (SHAP values +7.94 and +7.62) and the outcome is defined by BP thresholds, the manuscript must explicitly demonstrate what additional value machine learning provides over simple clinical protocols. Authors should conduct an ablation study showing model performance when BP features are excluded entirely, compare detection rates against current Tanzanian ANC guidelines, and provide concrete examples of cases where the integration of BMI, proteinuria, and other factors identified at-risk women who would have been missed by BP screening alone. This is essential to justify the added complexity and resource investment of ML deployment.

**Reviewer Comments (if any, and for reference):**

Reviewer's Responses to Questions

**Comments to the Author**

1. If the authors have adequately addressed your comments raised in a previous round of review and you feel that this manuscript is now acceptable for publication, you may indicate that here to bypass the “Comments to the Author” section, enter your conflict of interest statement in the “Confidential to Editor” section, and submit your "Accept" recommendation.

Reviewer #2: All comments have been addressed

Reviewer #4: All comments have been addressed

**********

2. Does this manuscript meet PLOS Digital Health’s publication criteria? Is the manuscript technically sound, and do the data support the conclusions? The manuscript must describe methodologically and ethically rigorous research with conclusions that are appropriately drawn based on the data presented.

Reviewer #2: Yes

Reviewer #4: Partly

**********

3. Has the statistical analysis been performed appropriately and rigorously?

Reviewer #2: Yes

Reviewer #4: Yes

**********

4. Have the authors made all data underlying the findings in their manuscript fully available (please refer to the Data Availability Statement at the start of the manuscript PDF file)?

The PLOS Data policy requires authors to make all data underlying the findings described in their manuscript fully available without restriction, with rare exception. The data should be provided as part of the manuscript or its supporting information, or deposited to a public repository. For example, in addition to summary statistics, the data points behind means, medians and variance measures should be available. If there are restrictions on publicly sharing data—e.g. participant privacy or use of data from a third party—those must be specified.

Reviewer #2: Yes

Reviewer #4: No

**********

5. Is the manuscript presented in an intelligible fashion and written in standard English?

PLOS Digital Health does not copyedit accepted manuscripts, so the language in submitted articles must be clear, correct, and unambiguous. Any typographical or grammatical errors should be corrected at revision, so please note any specific errors here.

Reviewer #2: Yes

Reviewer #4: Yes

**********

6. Review Comments to the Author

Please use the space provided to explain your answers to the questions above. You may also include additional comments for the author, including concerns about dual publication, research ethics, or publication ethics. (Please upload your review as an attachment if it exceeds 20,000 characters)

Reviewer #2: My comments have been adequately addressed.

Reviewer #4: The reframing from "early detection" to "risk stratification," addition of calibration analysis (Brier 0.08, slope 0.94), precision-recall curve, missing data table, operational burden quantification (6.3 FP per TP), and expanded comparison with fullPIERS, PIERS-ML, Li et al., and Chen and Ma are all meaningful improvements. One Critical issue persists, two Major issues are text-level corrections, and three Moderate plus five Minor items remain. None require new analysis.

Critical

C1. The systematic comparison of excluded versus included records (73 percent listwise exclusion of 136,864 of 187,438 client records) is described in the response to Reviewer 5 with t-tests and chi-square at p < 0.001 across comparisons, but is not formally presented in the manuscript. Please add this as a main-text or supplementary table with test statistics and p-values, and add a Discussion paragraph drawing the deployment implication: training and validation occurred on the 27 percent of clients with complete records, who systematically differ from excluded clients (the latter showing undertesting consistent with urgent or late presentation, the highest-risk subgroup), and performance in the broader ANC population is therefore unknown.

Major

M1. The acknowledgement that near-perfect training metrics (accuracy 100 percent, AUC 1.00) are an artifact of random undersampling and the algorithmic outcome label currently sits in the response letter only. Add one sentence in the Results paragraph or Table 2 caption stating this, noting that validation metrics (precision 14 percent, accuracy 90.95 percent, AUC 0.95) are the operationally meaningful figures.

M2. The class balancing method is reported inconsistently. The response to Reviewer 2 cites SMOTE oversampling; the response to Reviewer 5 states random undersampling to 1,894 cases plus 1,894 controls. The training count of 3,788 is consistent with undersampling. State the method once, precisely, in the Methods section, with the resulting training composition, and remove inconsistent references.

Moderate

Mod1 (Study Design). Add one Discussion paragraph characterising the missingness as MNAR (supported by the C1 analysis), stating that deployment underperformance is expected precisely on the highest-risk women, and recommending standardised proteinuria and glucose testing at first ANC visit as a prerequisite for deployment equity.

Mod2 (External Validity). The temporal validation cohort came from the same UCS facilities as training, which establishes temporal stability rather than geographic generalizability. Limit generalizability claims to the UCS facility network and introduce geographic external validation in rural or low-connectivity facilities outside current UCS coverage as a prerequisite for Ministry-level scaling.

Mod3 (Confounding and Bias). False positives have a mean systolic BP of 136.5 mmHg, which is 3.5 mmHg below the 140 mmHg threshold. Present these BP values as a formal Results element and reframe the added clinical value as identifying women with borderline BP within a clinically relevant margin of the threshold who may benefit from repeat measurement. "Identifying at-risk cases missed by conventional screening" overstates a 3.5 mmHg gap.

Minor

mn1. Add one sentence to the abstract noting calibration was assessed (Brier 0.08), consistent with a screening tool rather than a precision diagnostic.

mn2. Report the pre-RFE importance rank of gestational age to confirm its elimination was data driven.

mn3. Add a companion table reporting mean and 95 percent CI for each model's cross-validation AUC; the CIs on Figure 3 are not legible at current scale.

mn4. Apply at least one of the added ethical references (Rajkomar, Wiens, WHO) substantively in the Discussion to the documented exclusion bias.

mn5. Add the GitHub repository URL to the Data Availability statement or Methods section.

**********

7. PLOS authors have the option to publish the peer review history of their article (what does this mean?). If published, this will include your full peer review and any attached files.

**Do you want your identity to be public for this peer review?** If you choose “no”, your identity will remain anonymous but your review may still be made public.

For information about this choice, including consent withdrawal, please see our Privacy Policy.

Reviewer #2: **Yes:**Victor Ifechukwude Agboli

Reviewer #4: **Yes:**Julian Borges

**********

Attachment Submitted filename: PDIG-D-25-00485_reviewer_JULIAN_BORGES.pdf
